# *Psc* and *Su(z)2* safeguard intestinal stem cell identity and prevent *chinmo*-dependent tumorigenesis

**DOI:** 10.1038/s44319-026-00837-x

**Published:** 2026-06-20

**Authors:** Ruxue Wei, Haimeng Yu, Qingyu Sun, Yongchao Zhang, Yaxin Yu, Rongwen Xi

**Affiliations:** 1 National Institute of Biological Sciences, No. 7 Science Park Road, Zhongguancun Life Science Park, Beijing, 102206 China; 2 School of Life Sciences, Peking University, Beijing, 100871 China; 3 Tsinghua Institute of Multidisciplinary Biomedical Research, Tsinghua University, Beijing, 102206 China

**Keywords:** Cancer, Chromatin, Transcription & Genomics, Stem Cells & Regenerative Medicine

## Abstract

Polycomb group (PcG) genes are epigenetic silencers that maintain transcriptional repression of target genes essential for normal development. However, their roles in adult multipotent stem cell lineages remain poorly understood. Here, we show that simultaneous loss of the PRC1 component Psc and its homolog Su(z)2 in intestinal stem cells (ISCs) of the adult *Drosophila* midgut leads to tumor formation composed of proliferative, undifferentiated cells. Strikingly, these tumor cells do not activate proliferation-associated pathways, including JAK/STAT, Ras/MAPK, and Wnt, nor do they activate Notch or JAK/STAT signaling, which are essential for ISC differentiation. Transcriptomic and chromatin accessibility profiling reveal widespread downregulation of ISC and progenitor cell identity genes and ectopic activation of neural lineage genes. Among these, *chinmo* is aberrantly upregulated and required for tumor overgrowth. Notably, loss of other PRC1 components does not recapitulate the tumor phenotype, suggesting that the tumor-suppressive role of Psc and Su(z)2 is independent of canonical PRC1 function. Together, our findings uncover a noncanonical, context-specific tumor-suppressive role for Psc and Su(z)2 in preserving ISC identity and restricting lineage deviation.

## Synopsis

Intestinal stem cells (ISCs) mutant for Psc and Su(z)2 lose their stem cell identity and aberrantly express *chinmo*, leading to the formation of small-cell tumors. Notably, mutations in other PRC1 components neither induce *chinmo* expression nor promote tumor development.

- *Psc-Su(z)2* mutant ISCs develop into small cell tumors that are not driven by self-renewal signaling pathways.
- Loss of *Psc-Su(z)2* leads to the loss of ISC identity and the acquisition of neural-related gene expression.
- The ectopic expression of *chinmo* is required for tumorigenesis.
- Mutations in other PRC1 genes do not lead to *chinmo* activation or tumorigenesis.

## Abstract (web summary)

Intestinal stem cells (ISCs) mutant for Psc and Su(z)2 lose their stem cell identity and aberrantly express *chinmo*, leading to the formation of small-cell tumors. Notably, mutations in other PRC1 components neither induce *chinmo* expression nor promote tumor development.

## Graphical abstract

**Figure:**
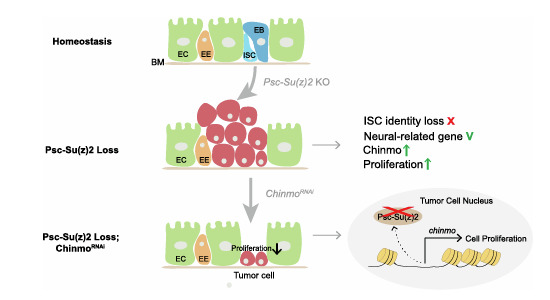

**Figure:**
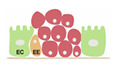

## Introduction

Polycomb group (PcG) genes, originally identified in *Drosophila*, encode epigenetic silencers that maintain the repressive state of Hox genes (Muller and Kassis, 2006). This gene-silencing function is mediated by two major protein complexes: Polycomb repressive complex 1 (PRC1) and Polycomb repressive complex 2 (PRC2). PRC2 functions as a histone methyltransferase, catalyzing the mono-, di-, and tri-methylation of histone H3 on lysine 27 (H3K27me1, H3K27me2, and H3K27me3). PRC1, which possesses E3 ubiquitin ligase activity, recognizes and binds to H3K27me3 and catalyzes mono-ubiquitination of histone H2A at lysine 118 (H2AK118ub1) in *Drosophila* or lysine 119 in mammals (Margueron and Reinberg, 2011). Beyond their developmental roles, PcG genes are also important for maintaining adult tissue homeostasis, and their dysregulation has been implicated in cancer (Piunti and Shilatifard, 2021). Their oncogenic function is primarily mediated through the transcriptional repression of the tumor suppressor locus INK4A/ARF (Bracken et al, 2007; Jacobs et al, 1999). As a result, inhibitors targeting PRC1 and PRC2 have been developed for clinical application in cancer therapy (Comet et al, 2016; Piunti and Shilatifard, 2021). However, accumulating evidence suggests that PcG genes can also function as tumor suppressors in various tissues and organs—a dual role that remains incompletely understood.

The tumor-suppressive function of PRC1 has recently been highlighted by several studies in *Drosophila*. The core components of *Drosophila* PRC1 include Polycomb (Pc), polyhomeotic (Ph), sex combs extra (Sce), and posterior sex combs (Psc)/suppressor of zeste two (Su(z)2). Mutations in these genes within the imaginal disc cells of *Drosophila* larvae result in tumorous tissue overgrowth, driven by hyperactivation of the JAK/STAT and Notch signaling pathways (Classen et al, 2009; Martinez et al, 2009). Additionally, simultaneously loss of Psc and Su(z)2 in epithelial follicle stem cells in adult *Drosophila* ovary leads to tumorigenesis, primarily through activation of both canonical and noncanonical Wnt signaling pathways (Li et al, 2010). Interestingly, loss of other PRC1 components in follicle stem cells does not result in tumor formation (Li et al, 2010). These observations raise important questions about whether PRC1’s tumor-suppressive function is broadly applicable to epithelial tissues, and whether this function is mediated by the canonical PRC1 complex or by specific PcG genes acting independently of PRC1, particularly in adult tissues and organs.

**Figure 1:**
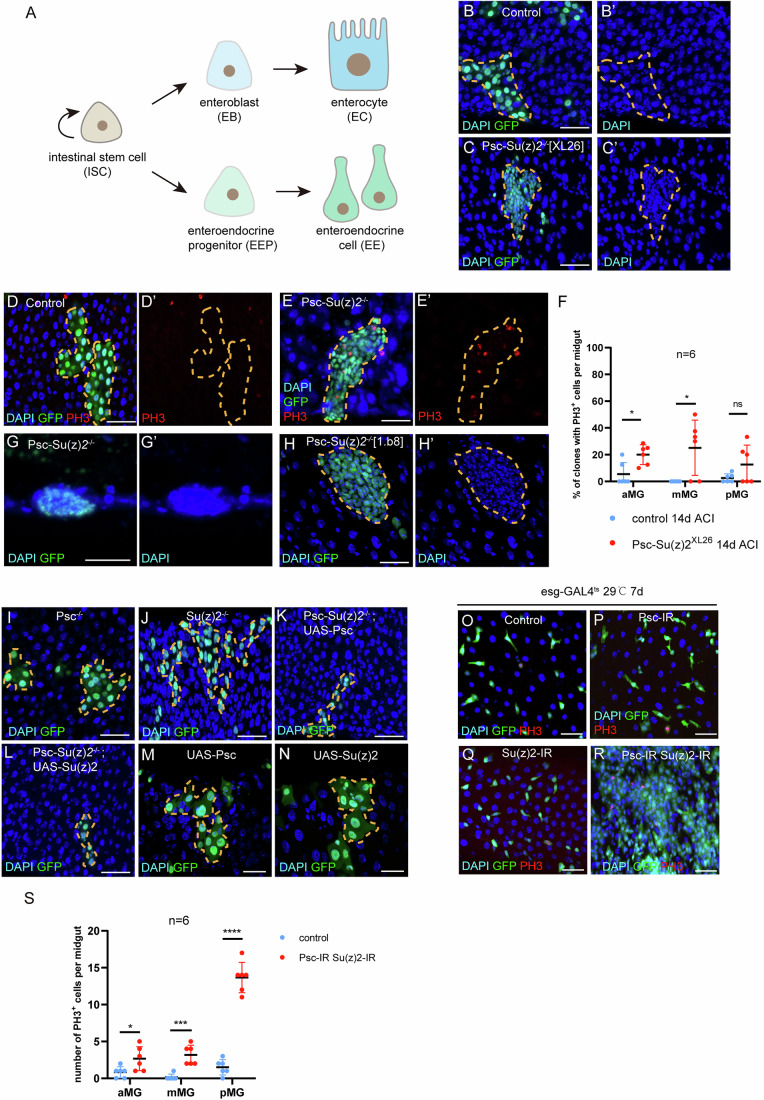
Loss of *Psc-Su(z)2* in ISCs leads to the formation of small-cell tumors. (**A**) A schematic overview of the ISC lineage in *Drosophila* midgut. (**B**,** C**) MARCM clones marked by GFP and co-stained with DAPI (blue) of wild-type *FRT 42D* (**B**) and *Psc-Su(z)2*^*XL26*^ *FRT 42D* (**C**) flies at 14 days ACI. Dashed lines depict the clone margin. ACI, after clone induction. (**D**,** E**) MARCM clones co-stained with DAPI (blue) and anti-Phospho histone3 (PH3, red) in wild-type *FRT 42D* (**D**) and *Psc-Su(z)2*^*XL26*^ *FRT 42D* (**E**) flies at 14 days ACI. Dashed lines depict the clone margin. (**F**) Quantification of the percentage of clones containing PH3^+^ cells along the anterior (aMG), middle (mMG) and posterior (pMG) regions of the midgut at 14 days ACI. Data were shown for both wild-type *FRT 42D* and *Psc-Su(z)2*^*XL26*^ *FRT 42*D clones. Data were obtained from three independent biological experiments (*N* = 3), with *n* = 6 guts analyzed per genotype. *p* values: aMG: *p* = 0.0106, mMG: *p* = 0.0137, pMG: *p* = 0.1294. ns, not significant. Note: One experimental replicate performed under stressed conditions is shown in Fig. EV1E. (**G**) Representative cross-section images showing GFP expressing MARCM clones of *Psc-Su(z)2*^*XL26*^ *FRT 42D* flies at 14 days ACI. (**H**) MARCM clones co-stained with DAPI (blue) of *Su(z)2*^*1.b8*^*FRT 42D* flies at 14 days ACI. Dashed lines depict the clone margin. (**I**–**N**) MARCM clones co-stained with DAPI (blue) at 14 days ACI of the following genotypes: *Psc*^*h27*^ *FRT 42D* (**I**), *Su(z)2*^*RNAi*^ *FRT 82B* (**J**), *Psc*-*Su(z)2*^*XL26*^*; UAS-Psc FRT 42D* (**K**), *Psc*-*Su(z)2*^*XL26*^*; UAS-Su(z)2 FRT 42D* (**L**), Control *FRT 42D; UAS-Psc* (**M**), and Control *FRT 42D; UAS-Su(z)2 FRT 42D* (**N**). Dashed lines depict the clone margin. (**O**–**R**) Representative images staining with DAPI (blue) and anti-Phospho histone3 (PH3, red) of the following genotypes: *esg*^*ts*^ > *GFP* control (**O**), *esg*^*ts*^ > *GFP; Psc RNAi* (**P**), *esg*^*ts*^ > *GFP; Su(z)2 RNAi* (**Q**) and *esg*^*ts*^ > *GFP; Psc-Su(z)2 RNAi*. Flies were dissected at 7 days after eclosion. (**S**) Quantification of the number of PH3^+^ cells along the anterior (aMG), middle (mMG) and posterior (pMG) regions of the midgut at 14 days ACI. Data are shown for both control and *Psc-Su(z)2 RNAi* flies. Data were obtained from three independent biological experiments (*N* = 3), with *n* = 6 guts analyzed per genotype. *p* values: aMG: *p* = 0.0316, mMG: *p* = 0.0004, pMG: *p* < 0.0001. ns, not significant. Significance was measured with unpaired two-tailed *t*-tests. Mean ± SEM. ^ns^*p*>0.05; ^**^p < 0.01; ^***^p < 0.001; ^****^p < 0.0001. Scale bars, 25 μm. See also Figs. EV1 and EV2. Source data are available online for this figure.

Another continuously renewing epithelial tissue in adult *Drosophila* is the midgut epithelium, which is maintained by resident intestinal stem cells (ISCs) (Micchelli and Perrimon, 2006; Ohlstein and Spradling, 2006). ISCs divide periodically to produce either enteroblasts (EBs) or enteroendocrine progenitor cells (EEPs), which subsequently differentiate into polyploid enterocytes (ECs) or diploid enteroendocrine (EE) cells, respectively (Fig. 1A). ISCs specifically express the Notch receptor ligand Delta (Dl), and by default, ISC division results in one daughter cell receiving the Dl signal and adopting an EB fate via Notch activation, eventually differentiating into an EC (Ohlstein and Spradling, 2007). Periodically, a feedback loop induces transient Scute activation in ISCs, prompting the generation of an EEP instead of an EB. The EEP then undergoes a single division prior to terminal differentiation, producing a pair of EE cells (Chen et al, 2018). ISC proliferation is tightly regulated by multiple signaling pathways, including Wnt, JAK/STAT, and EGFR/Ras/MAPK (Beebe et al, 2010; Biteau and Jasper, 2011; Jiang et al, 2011; Jiang et al, 2009; Liang et al, 2017; Lin et al, 2008, 2010; Xu et al, 2011). In response to tissue damage or infection, additional pathways such as JNK and Hippo/Yki are activated to boost ISC proliferation (Panayidou and Apidianakis, 2013). While Notch signaling is essential for EC specification, the JAK/STAT pathway is required for the differentiation of both ECs and EEs (Beebe et al, 2010; Jiang et al, 2009; Lin et al, 2010). Dysregulation of many of these signaling pathways can impair ISC lineage differentiation, ultimately leading to dysplasia or tumorigenesis (Cordero et al, 2012; Lee et al, 2009; Wang et al, 2013).

Here, we report that loss of the PRC1 component Psc and its homolog Su(z)2 in ISCs completely blocks ISC differentiation. These undifferentiated cells continue to proliferate, leading to rapid tumor formation. Notably, this tumorigenesis is not driven by aberrant activation of the Wnt, JAK/STAT, or EGFR/Ras/MAPK signaling pathways. Instead, it is critically dependent on the ectopic activation of Chinmo, a neuronal cell division regulator. Moreover, our analyses also suggest that the tumor-suppressive function of *Psc* and *Su(z)2* appears to be independent of the canonical PRC1 complex, indicating a noncanonical role for these PcG genes in maintaining epithelial tissue homeostasis.

## Results

### *Psc-Su(z)2* mutant ISCs develop into small-cell tumors

**Figure EV1:**
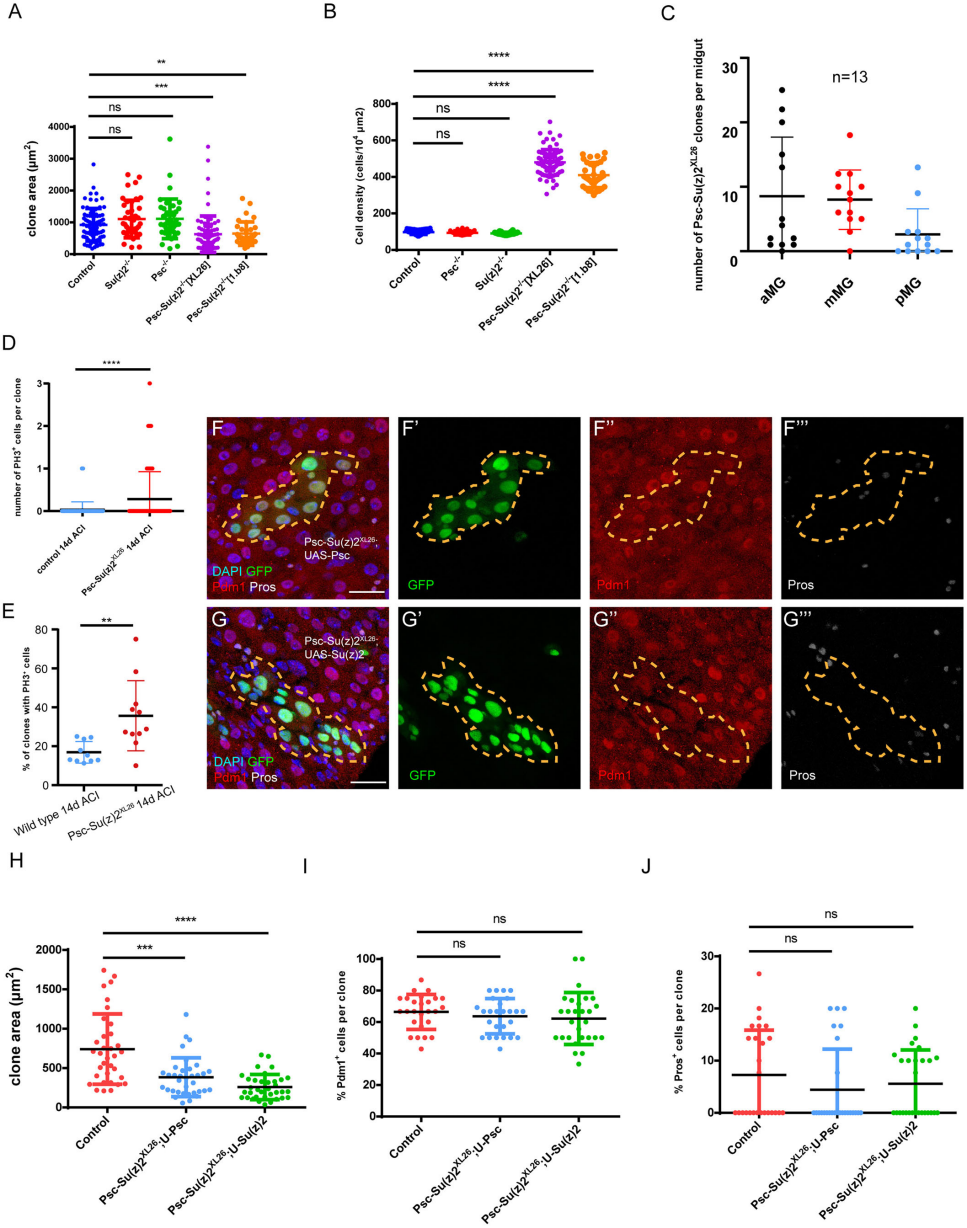
Analysis of *Psc-Su(z)2* mutant clones with or without transgene expression. (**A**) Quantification of the clone area at 14 days ACI for wild-type *FRT 42D, Su(z)2*^*RNAi*^ *FRT 82B, Psc*^*h27*^ *FRT 42D, Psc-Su(z)2*^*XL26*^ and *Su(z)2*^*1.b8*^. Data were obtained from three independent biological experiments (*N* = 3), with the following samples analyzed per genotype: wild-type *FRT 42D*: 77 clones from 5 guts; *Su(z)2*^*RNAi*^ *FRT 82B:* 40 clones from 5 guts*; psc*^*h27*^ *FRT 42D:* 41 clones from 5 guts*; Psc-Su(z)2*^*XL26*^: 79 clones from 5 guts; *Su(z)2*^*1.b8*^*:* 34 clones from 5 guts. ACI, after clone induction. *p* values: *Psc-Su(z)2*^*XL26*^: *p* = 0.0008, *Su(z)2*^*1.b8*^: p = 0.0055. (**B**) Quantification of clone cell density (number of cells per clone area) at 14 days ACI for wild-type *FRT 42D, Su(z)2*^*RNAi*^ *FRT 82B, Psc*^*h27*^ *FRT 42D, Psc-Su(z)2*^*XL26*,^ and *Su(z)2*^*1.b8*^. Data were obtained from three independent biological experiments (*N* = 3), with the following samples analyzed per genotype: wild-type *FRT 42D*: 77 clones from five guts; *Su(z)2*^*RNAi*^ *FRT 82B:* 40 clones from five guts*; Psc*^*h27*^ *FRT 42D:* 41 clones from five guts*; Psc-Su(z)2*^*XL26*^: 79 clones from five guts; *Su(z)2*^*1.b8*^*:* 34 clones from five guts. ACI, after clone induction. *p* values: *Psc-Su(z)2*^*XL26*^: *p* < 0.0001, *Su(z)2*^*1.b8*^: *p* < 0.0001. (**C**) Quantification of the number of clones at 14 days ACI along the anterior (aMG), middle (mMG) and posterior (pMG) regions of the midgut in *Psc*-*Su(z)2*^*XL26*^ flies. Data were obtained from three independent biological experiments (*N* = 3), with *n* = 13 guts analyzed per genotype. (**D**) Quantification of the PH3^+^ cell number per clone at 14 days ACI of wild-type *FRT 42D* and *Psc*-*Su(z)2*^*XL26*^ flies. Data were obtained from three independent biological experiments (*N* = 3), with the following samples analyzed per genotype: control 14 d ACI: 205 clones from six guts, *Psc-Su(z)2*^*XL26*^ 14 d ACI: 155 clones from six guts. ACI, after clone induction. *p* < 0.0001. (**E**) Quantification for the percentage of clones containing PH3^+^ cells per midgut. Data were obtained from three independent biological experiments (*N* = 3), with the following samples analyzed per genotype: wild-type 14 d ACI: ten clones from five guts; *Psc-Su(z)2*^*XL26*^ 14 d ACI: 11 clones from five guts. ACI, after clone induction. *p* = 0.0053. Note: In this set of experiments, ISCs in both control and experimental flies exhibited evidently elevated baseline activity. This is likely attributable to unintended midgut stress during the 14-day room temperature incubation after heat shock. Data obtained under physiological conditions are presented in Fig. 1F. (**F**,** G**) MARCM clones marked by GFP and co-stained with DAPI (blue), anti-Pdm1 (red) and anti-Pros (white) antibodies in *Psc*-*Su(z)2*^*XL26*^*; UAS-Su(z)2 FRT 42D* (D) and *Psc*-*Su(z)2*^*XL26*^*; UAS-Psc FRT 42D* (E) flies at 14 days ACI. Dashed lines depict the clone margin. (**H**) Quantification of the clone area in wild-type *FRT 42D, Psc*-*Su(z)2*^*XL26*^*; UAS-Psc FRT 42D* and *Psc*-*Su(z)2*^*XL26*^*; UAS-Su(z)2 FRT 42D* clones at 14 days ACI. Data were obtained from three independent biological experiments (*N* = 3), with the following samples analyzed per genotype: control: 34 clones from five guts, *Psc-Su(z)2*^*XL26*^; *UAS-Psc FRT 42D*: 35 clones from 5 guts, *Psc-Su(z)2*^*XL26*^; *UAS-Su(z)2 FRT 42D*: 34 clones from five guts. ACI after clone induction. *p* values: *Psc-Su(z)2*^*XL26*^; *UAS-Psc FRT 42D*: *p* = 0.0001; *Psc-Su(z)2*^*XL26*^; *UAS-Su(z)2 FRT 42D*: *p* < 0.0001. (**I**,** J**) Quantification of the proportion of Pdm1^+^ cells (**G**) and Pros^+^ cells (**H**) per clone in 14-day clones of wild-type *FRT 42D, Psc*-*Su(z)2*^*XL26*^*; UAS-Psc FRT 42D* and *Psc*-*Su(z)2*^*XL26*^*; UAS-Su(z)2 FRT 42D* flies. Data were obtained from three independent biological experiments (*N* = 3), with the following samples analyzed per genotype: control: 26 clones from six guts, *Psc-Su(z)2*^*XL26*^; *UAS-Psc FRT 42D*: 26 clones from 6 guts, *Psc-Su(z)2*^*XL26*^; *UAS-Su(z)2 FRT 42D*: 28 clones from five guts. Significance was measured with unpaired two-tailed *t*-tests. Mean ± SEM. ^ns^*p* > 0.05; ^**^p < 0.01; ^***^p < 0.001; ^****^p < 0.0001. Scale bars, 25 μm.

We first employed the mosaic analysis with a repressible cell marker (MARCM) system to generate green fluorescent protein (GFP)-labeled *Psc Su(z)2* double mutant ISC clones in the adult *Drosophila* midgut. *Psc* and *Su(z)2* are known to have redundant functions in adult tissues (Li et al, 2010). In contrast to control clones, which predominantly consist of polyploid ECs, *Su(z)2*^*XL26*^ mutant clones— in which the coding regions of both *Psc* and *Su(z)2* are precisely deleted—contained numerous small diploid cells (Fig. 1B,C). Of note, owing to the small cell size (approximately 20 μm², comparable to that of ISCs), the total area of each clone does not increase as markedly as the total cell number. However, cell density is significantly increased. (Fig. EV1A,B). We therefore term these clones as “small cell clusters”. These mutant cells were densely packed, multilayered, and highly proliferative (Figs. 1D–F and EV1D,E), exhibiting a tumor-like phenotype reminiscent of mammalian intestinal adenomas (Fig. 1G). Elevated proliferation was observed in *Su(z)2*^*XL26*^ mutant clones under both physiological conditions (Fig. 1F) and gut stress conditions (Fig. EV1E). The small cell clusters were found along the length of the midgut, with more prominent tumorous outgrowth in the anterior and middle midgut regions (Fig. EV1C). A similar phenotype was observed in *Su(z)2*^*1.b8*^ clones (Fig. 1H), an allele with a chromosomal deletion that removes *Psc*, *Su(z)2*, and several other genes (Adler et al, 1989). In contrast, mutation of *Psc* or *Su(z)2* alone did not produce this small-cell tumor phenotype (Fig. 1I,J). Furthermore, transgenic expression of either *Psc* or *Su(z)2* effectively restored normal proliferation and differentiation of cells within the mutant clones, fully rescuing the phenotype caused by *Su(z)2*^*XL26*^ (Figs. 1K–N and EV1F–J), These results confirm that *Psc* and *Su(z)2* function redundantly to suppress small-cell tumor formation in ISCs.

**Figure EV2:**
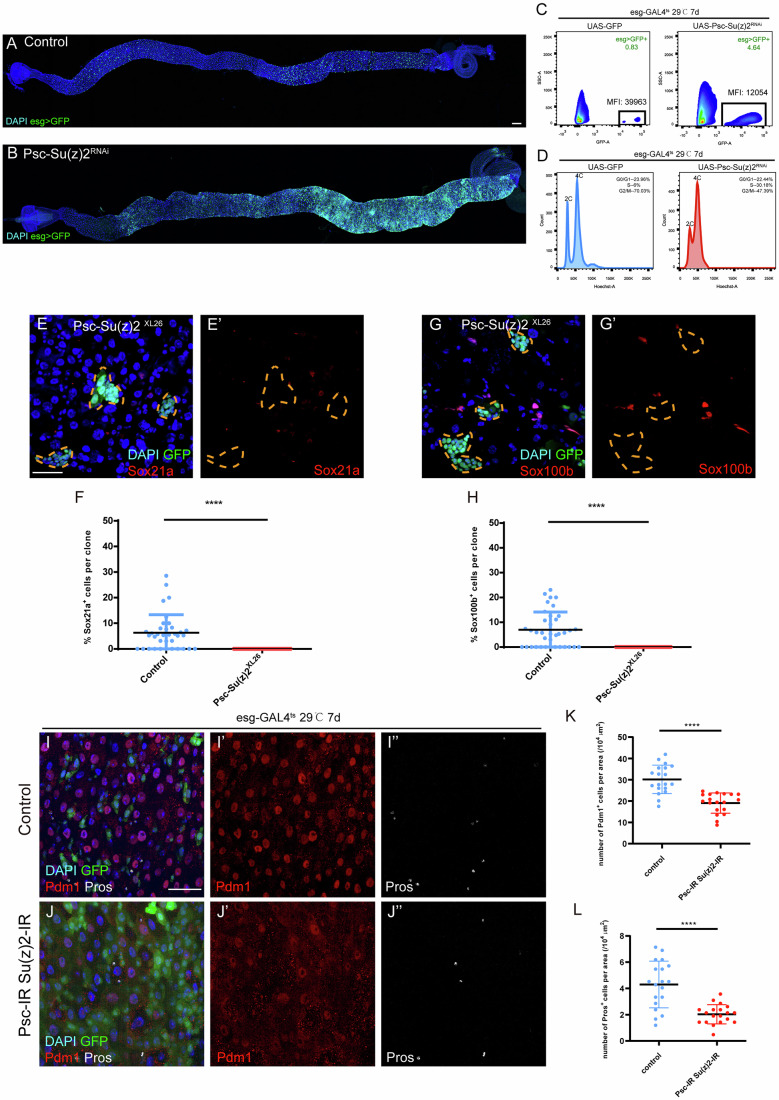
Tumor cells in *Psc*-*Su(z)2* mutants are diploid and highly proliferative. (**A**,** B**) Representative images of the whole gut of the following genotypes of flies stained with DAPI (blue): wild-type *esg*^*ts*^ > *GFP* (**A**) and *esg*^*ts*^*>Psc-Su(z)2*^*RNAi*^ (**B**). (**C**) FACS analysis of the proportion of *esg* > *GFP* cells in guts of *esg*^*ts*^ > *GFP* and *esg*^*ts*^*>Psc-Su(z)2*^*RNAi*^ flies. (**D**) FACS analysis of the cell cycle of *esg*^*ts*^ > *GFP* and *esg*^*ts*^*>Psc-Su(z)2*^*RNAi*^ cells. (**E**) MARCM clones marked by GFP co-stained with DAPI (blue) and anti-Sox21a (red) in *Psc-Su(z)2*^*XL26*^ *FRT 42D* flies at 14 days ACI. Dashed lines depict the clone margin. ACI, after clone induction. (**F**) Quantification of the percentage of Sox21a^+^ cells per clone in 14-day clones of wild-type *FRT 42D* and *Psc*-*Su(z)2*^*XL26*^ flies. Data were obtained from three independent biological experiments (*N* = 3), with the following samples analyzed per genotype: wild-type *FRT 42D*: 36 clones from six guts and *Psc-Su(z)2*^*XL26*^: 39 clones from six guts. *p* < 0.0001. (**G**) MARCM clones co-stained with DAPI (blue) and anti-Sox100b (red) in *Psc-Su(z)2*^*XL26*^ *FRT 42D* flies at 14 days ACI. Dashed lines depict the clone margin. (**H**) Quantification of the percentage of Sox100b^+^ cells per clone in 14-day clones of wild-type *FRT 42D* and *Psc*-*Su(z)2*^*XL26*^ flies. Data were obtained from three independent biological experiments (*N* = 3), with the following samples analyzed per genotype: wild-type *FRT 42D*: 37 clones from six guts and *Psc-Su(z)2*^*XL26*^: 39 clones from six guts. *p* < 0.0001. (**I**,** J**) Representative images staining with DAPI (blue), anti-Pdm1 (red) and anti-Pros (white) antibodies of *esg*^*ts*^ > *GFP* control (**I**) and *esg*^*ts*^ > *GFP; Psc-Su(z)2 RNAi* (**J**) flies. Flies were dissected at 7 days after eclosion. (**K**,** L**) Quantification of (**I**, **J**): numbers of Pdm1^+^ cells (**K**) and Pros^+^ cells (**L**) per area (10^4^ µm^2^) in the midgut at 14 days ACI. Data were obtained from three independent biological experiments (*N* = 3), with 19 areas from five guts per genotype. ACI, after clone induction. Significance was measured with unpaired two-tailed *t*-tests. *p* < 0.0001. Mean ± SEM. ^ns^*p* > 0.05; ^**^p < 0.01; ^***^p < 0.001; ^****^p < 0.0001. Scale bars, 25 μm.

Consistent with their redundant roles, conditional knockdown of both *Psc* and *Su(z)2*—but not either gene alone—in intestinal progenitor cells (ISCs and EBs), using the esg-GAL4 driver, led to marked cell overproliferation throughout the midgut (Figs. 1O–S and EV2A,B). Fluorescence-activated cell sorting (FACS) analysis of gut epithelial cells revealed a significant increase in the proportion of GFP^+^ cells, from 0.83% in controls to 4.64% following double knockdown of *Psc* and *Su(z)2* (Fig. EV2C), indicating accumulation of undifferentiated progenitor cells. Notably, the mean GFP fluorescence intensity in these mutant cells was generally reduced to one-third of the control level—from 39,963 to 12,054 (Fig. EV2C), indicating a loss of progenitor cell identity. Consistently, immunostaining for the additional progenitor cell markers Sox21a and Sox100b revealed a marked reduction of both proteins within *Psc-Su(z)2* mutant clones (Fig. EV2E–H). Additionally, cell cycle analysis of the undifferentiated progenitor cells in the double RNAi gut showed a significant increase in the proportion of cells in S phase (Fig. EV2D), indicating increased DNA synthesis and continuous cell division, which explains the tumorous outgrowth phenotype following the loss of *Psc* and *Su(z)2*.

### *Psc-Su(z)2* mutant ISCs are blocked in cell differentiation

**Figure 2:**
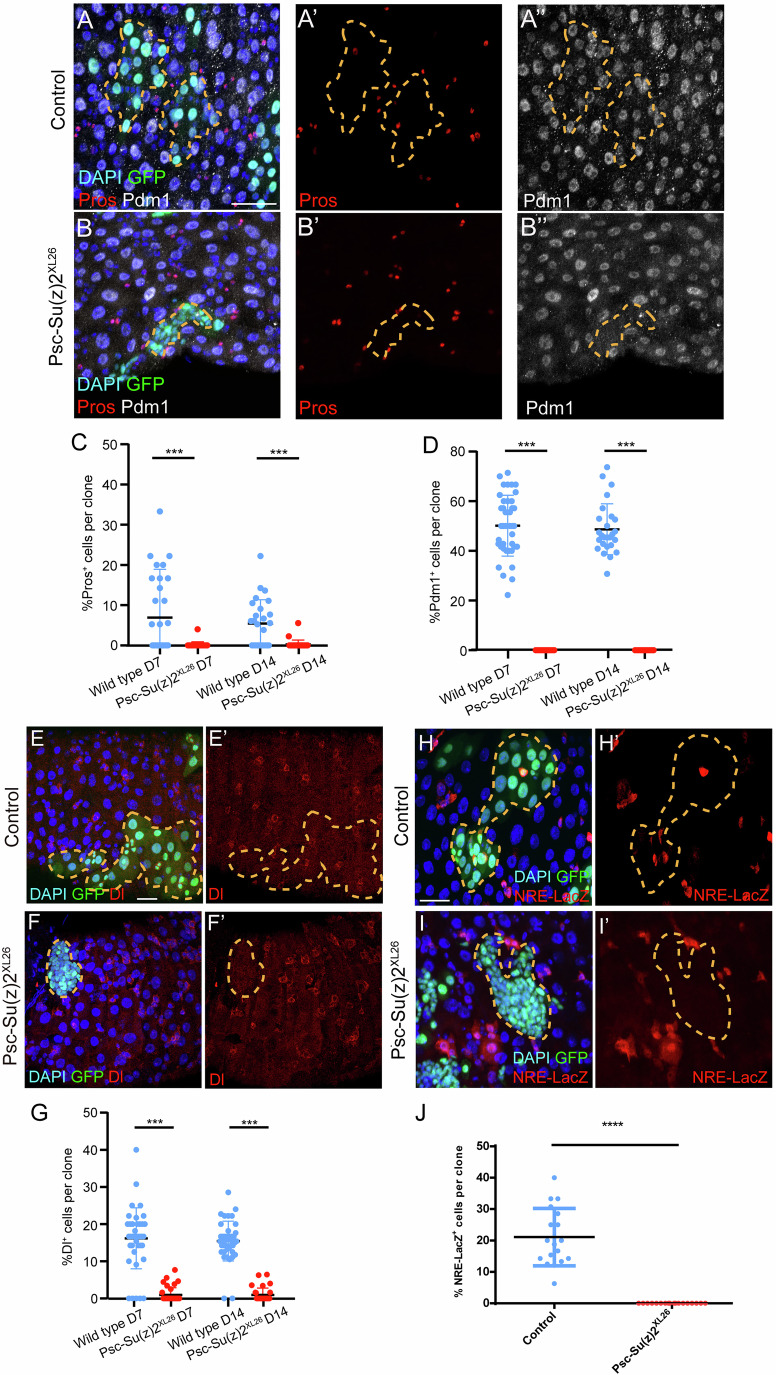
Loss of *Psc-Su(z)2* results in a block in differentiation. (**A**,** B**) MARCM clones marked by GFP co-stained with DAPI (blue), anti-Pros (red), and anti-Pdm1 (white) antibodies in wild-type *FRT 42D* (**A**) and *Psc-Su(z)2*^*XL26*^ *FRT 42D* (**B**) flies at 14 days ACI. Dashed lines depict the clone margin. ACI after clone induction. (**C**,** D**) Quantification of the proportion of Pros^+^ cells (**C**) and Pdm1^+^ cells (**D**) per clone in 7-day and 14-day clones of wild-type *FRT 42D* and *Psc-Su(z)2*^*XL26*^ *FRT 42D* flies (7D clones: wild-type *FRT 42D*: 45 clones from six guts, *Psc-Su(z)2*^*XL26*^ *FRT 42D*: 30 clones from five guts; 14D clones: wild-type *FRT 42D*: 27 clones from five guts, *Psc-Su(z)2*^*XL26*^ *FRT 42D*: 30 clones from five guts). *p* values (**C**): 7D: *p* = 0.0003, 14D: *p* = 0.0001. *p* values (**D**): *p* = 0.0002, 14D: *p* = 0.0001. (**E**,** F**) MARCM clones co-stained with DAPI (blue) and anti-Dl (red) antibody in wild-type *FRT 42D* (**E**) and *Psc-Su(z)2*^*XL26*^ *FRT 42D* (**F**) flies at 14 days ACI. Dashed lines depict the clone margin. (**G**) Quantification of the proportion of Dl^+^ cells per clone in 7-day and 14-day clones of wild-type *FRT 42D* and *Psc-Su(z)2*^*XL26*^ *FRT 42D* flies (7D clones: wild-type *FRT 42D*: 43 clones from six guts, *Psc-Su(z)2*^*XL26*^ *FRT 42D*: 35 clones from five guts; 14D clones: wild-type *FRT 42D*: 41 clones from six guts, *Psc-Su(z)2*^*XL26*^ *FRT 42D*: 31 clones from five guts). *p* values: 7D: *p* = 0.0001, 14D: *p* = 0.0001. (**H**,** I**) MARCM clones co-stained with DAPI (blue) and anti-LacZ (red) antibody in Control *FRT 42D*; NRE-LacZ (**A**) and *Psc-Su(z)2*^*XL26*^; NRE-LacZ *FRT 42D* (**I**) flies at 14 days ACI. Dashed lines depict the clone margin. (**J**) Quantification of (**H**) and (**I**): percentage of NRE-LacZ^+^ cells per clone at 14 days ACI. Data were collected from three independent biological experiments (*N* = 3), with 18 clones from eight guts per genotype. *p* < 0.0001. Significance was measured with unpaired two-tailed *t*-tests. Mean ± SEM. ^ns^*p* > 0.05; ^**^p < 0.01; ^***^p < 0.001; ^****^p < 0.0001. Scale bars, 25 μm. See also Fig. EV2. Source data are available online for this figure.

Since the tumors were composed of small, proliferating cells, we hypothesized that the differentiation capacity of *Psc-Su(z)2*-deficient ISCs might be compromised. To test this, we performed immunostaining with Prospero (Pros), a marker for EEs, and Pdm1, a marker for ECs. The mutant clones were negative for both Pros and Pdm1, indicating a complete absence of differentiated cells within the *Psc-Su(z)2* tumors (Fig. 2A–D). Consistently, Pros^+^ and Pdm1^+^ cells were also significantly reduced in esg-GAL4, UAS-*Psc-*RNAi, UAS-*Su(z)2-*RNAi intestines (Fig. EV2I–L). Thus, loss of *Psc* and *Su(z)2* impairs ISC differentiation.

One key signaling pathway required for ISC differentiation is Dl-Notch signaling. Under normal conditions, Dl is specifically expressed in ISCs and activates Notch in their immediate daughter EBs, promoting their differentiation into ECs. Immunostaining showed that tumor cells lacked Dl expression (Fig. 2E–G), and none exhibited activation of Notch signaling, as evidenced by the absence of NRE-lacZ (short for Gbe-Su(H)m8-lacZ) expression, a Notch activity reporter (Fig. 2H–J). This failure to activate Dl-Notch signaling likely accounts for the inability of *Psc-Su(z)2*-deficient ISCs to generate ECs.

However, Notch signaling is not required for EE differentiation. In fact, loss of Notch signaling typically results in ISC overproliferation and excess EE differentiation, forming clusters of both ISC- or progenitor-like cells that resemble EE precursors and Pros^+^ EEs within the intestinal epithelium (Bardin et al, 2010; Chen et al, 2018; Micchelli and Perrimon, 2006; Ohlstein and Spradling, 2006; Patel et al, 2015). Therefore, the lack of EEs in *Psc-Su(z)2* mutant tumors suggests that, in addition to disrupted Dl-Notch signaling, other mechanisms must contribute to the failure of differentiation and the resulting tumor-like overgrowth from *Psc-Su(z)2*-deficient ISCs.

### *Psc-Su(z)2* mutant cells do not show increased JAK/STAT, Wnt or EGFR/MAPK signaling activities

**Figure 3:**
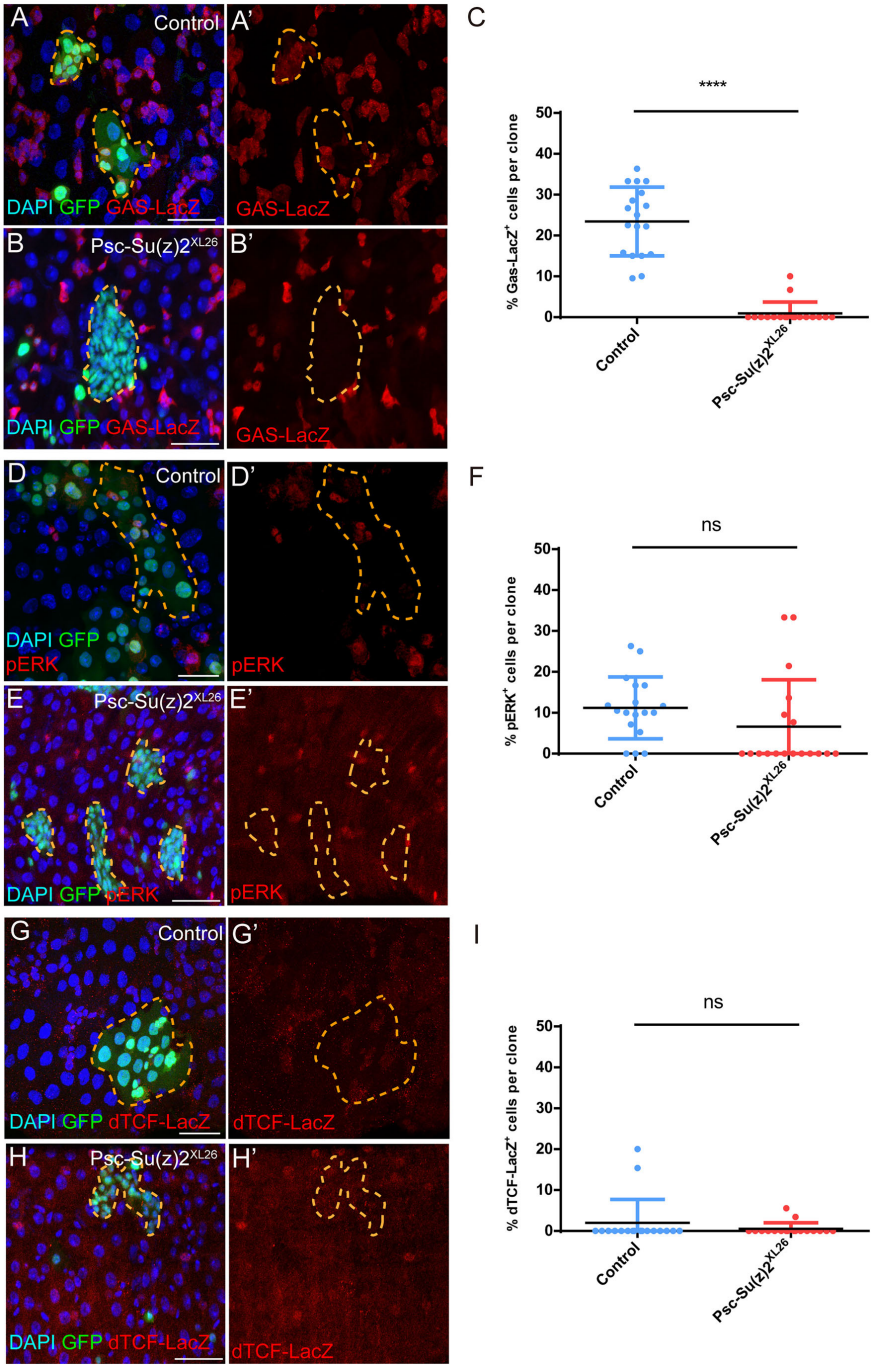
Loss of *Psc-Su(z)2* does not result in hyperactivation of JAK/STAT, EGFR /MAPK, or Wnt signaling pathways. (**A**,** B**) MARCM clones marked by GFP co-stained with DAPI (blue) and anti-LacZ (red) antibody in Control *FRT 42D*; GAS-LacZ (**A**) and *Psc-Su(z)2*^*XL26*^; GAS-LacZ *FRT 42D* (**B**) flies at 14 days ACI. Dashed lines depict the clone margin. ACI, after clone induction. (**C**) Quantification of (**A**, **B**): percentage of GAS-LacZ^+^ cells per clone at 14 days ACI. Data were collected from three independent biological experiments (*N* = 3), with 18 clones from six guts per genotype. *p* < 0.0001. (**D**,** E**) MARCM clones co-stained with DAPI (blue) and anti-Phospho ERK (pERK, red) antibody in Control *FRT 42D* (**D**) and *Psc-Su(z)2*^*XL26*^ *FRT 42D* (**E**) flies at 14 days ACI. Dashed lines depict the clone margin. (**F**) Quantification of (**D**, **E**): percentage of pERK^+^ cells per clone at 14 days ACI. Data were collected from three independent biological experiments (*N* = 3), with 18 clones from six guts per genotype. (**G**,** H**) MARCM clones co-stained with DAPI (blue) and anti-LacZ (red) antibody in Control *FRT 42D*; dTCF-LacZ (**G**) and *Psc-Su(z)2*^*XL26*^; dTCF-LacZ *FRT 42D* (**H**) flies at 14 days ACI. Dashed lines depict the clone margin. (**I**) Quantification of (**G**, **H**): percentage of dTCF-LacZ ^+^ cells per clone at 14 days ACI. Data were collected from three independent biological experiments (*N* = 3), with 18 clones from seven guts per genotype. Significance was measured with unpaired two-tailed *t*-tests. Mean ± SEM. ^ns^*p* > 0.05; ^**^p < 0.01; ^***^p < 0.001; ^****^p < 0.0001. Scale bars, 25 μm. See also Fig. EV3. Source data are available online for this figure.

Another key signaling pathway required for ISC differentiation is the JAK/STAT pathway. Disruption of this pathway impairs both EC and EE differentiation, leading to the accumulation of undifferentiated EBs. Strikingly, none of the *Psc-Su(z)2* tumor cells exhibited JAK/STAT signaling activity, as indicated by the absence of *GAS-lacZ* expression, a reporter for JAK/STAT activation (Fig. 3A–C). Thus, the simultaneous disruption of both Notch and JAK/STAT signaling pathways provides a mechanistic explanation for the differentiation blockade observed in *Psc-Su(z)2*-deficient ISCs.

Under normal conditions, JAK/STAT signaling also promotes ISC proliferation. To understand why *Psc-Su(z)2*-deficient ISCs are hyperproliferative despite lacking JAK/STAT activity, we investigated additional signaling pathways that are known to regulate ISC proliferation. These included Wnt signaling—whose hyperactivation is associated with intestinal hyperplasia—and EGFR/Ras/MAPK signaling, which is essential for ISC proliferation during both homeostasis and regenerative responses. Surprisingly, neither pathway showed evidence of strong activation in *Psc-Su(z)2* tumor cells. Compared with the wild-type control, immunostaining for phosphorylated ERK (pERK), a marker of EGFR/Ras/MAPK, revealed that Ras/MAPK signaling activity was significantly reduced, rather than increased, in *Psc-Su(z)2*-deficient clones (Fig. 3D–F). Similarly, expression of the *dTCF-lacZ* reporter, an indicator of Wnt pathway activity, remained at baseline levels both within and outside the clones (Fig. 3G–I). This raises an intriguing question: what alternative mechanisms are driving the tumor-like overgrowth in *Psc-Su(z)2*-deficient ISCs in the absence of canonical proliferative signaling pathways?

### Loss of *Psc* and *Su(z)2* reprograms ISCs into abnormal cells with neural-like gene expression

**Figure 4:**
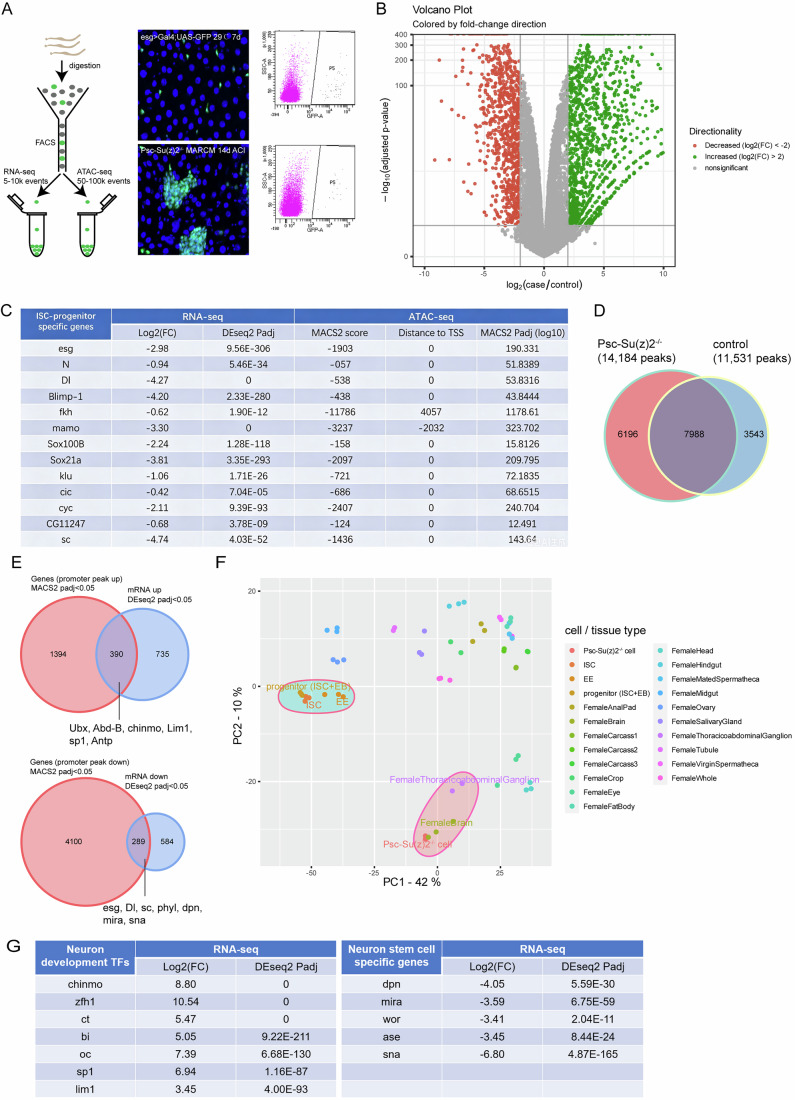
*Psc-Su(z)2* deficiency leads to a shift in cell identity from ISCs to abnormal cells exhibiting neural-like characteristics. (**A**) A schematic diagram of RNA-seq and ATAC-seq of wild-type *esg*^*ts*^ > *GFP*^*+*^ cells and *Psc-Su(z)2* deleted cells. (**B**) A volcano plot shows the comparison of gene expression profiles of wild-type esg^+^ and *Psc-Su(z)2*^*−/−*^ cells. Red dots depict decreased genes (log (FC) >2), and green dots depict increased genes (log (FC) <−2). Both WT esg^+^ and *Psc-Su(z)2*^*−/−*^ cells datasets have three biological replicates(differential expression analysis performed using DESeq2). (**C**) A table listing transcription and chromatin accessibility changes of progenitor-specific genes in *Psc-Su(z)2* deleted ISCs. (**D**) A Venn diagram showing the respective and overlapped chromatin opened peaks of wild-type esg^+^ and *Psc-Su(z)2*^*−/−*^ cells according to ATAC-seq datasets. The red circle depicts *Psc-Su(z)2*^*−/−*^ cells datasets, the blue circle depicts esg^+^ cells datasets. Both esg^+^ and *Psc-Su(z)2*^*−/−*^ cells datasets have three biological replicates. (**E**) Venn diagram (up) shows the overlap between genes with significantly opened peaks in promoter regions via ATAC-seq analysis in *Psc-Su(z)2*^*−/*^^−^ cells (red circle) and increased mRNAs (log (FC) >2) via RNA-seq analysis in *Psc-Su(z)2*^*−/−*^ cells (blue circle). Venn diagram (down) shows the overlap between genes with significant closed peaks in promoter regions via ATAC-seq analysis in *Psc-Su(z)2*^*−/−*^ cells (red circle) and decreased mRNAs (log (FC) <−2) via RNA-seq analysis in *Psc-Su(z)2*^*−/−*^ cells (blue circle). (**F**) PCA analysis of *Psc-Su(z)2*^*−/−*^ cells RNA-seq datasets with intestinal ISCs and EEs, as well as different *Drosophila* tissues datasets (from FlyAtlas 2). (**G**) Table listing expression levels of neuron development transcription factors and neuron stem cell- specific genes in *Psc-Su(z)2* mutant ISCs compared to wild-type esg^+^ cells via RNA-seq datasets. Source data are available online for this figure.

To further investigate the characteristics of the tumor cells, we employed genome-wide profiling approaches. GFP^+^ cells were FACS-sorted from wild-type *esg-GAL4, UAS-GFP* midguts and from *Psc-Su(z)2*^*−/−*^ MARCM clones, followed by bulk RNA sequencing (RNA-seq) and ATAC-seq (assay for transposase-accessible chromatin using sequencing) analyses (Fig. 4A). Compared with wild-type cells, *Psc-Su(z)2*^*−/−*^cells showed substantial transcriptomic changes, with 3437 genes significantly upregulated and 3690 genes downregulated (Dataset EV1; Fig. 4B). Notably, many key marker genes of intestinal ISC and progenitor identity—particularly transcription factors such as *esg, Sox21a, Sox100B, cic, sc*, and *klu*—were markedly downregulated in *Psc-Su(z)2*^*−/−*^ cells (Fig. 4C). These findings suggest that loss of *Psc* and *Su(z)2* leads to a loss of progenitor cell identity. The observed downregulation of esg also explains the reduced GFP fluorescence in the *esg-GAL4*^*ts*^*, UAS-GFP; UAS-psc-RNAi,* and *UAS-Su(z)2-RNAi* midguts reported earlier.

ATAC-seq analysis identified 11,531 accessible chromatin peaks in control cells and 14,184 peaks in *Psc-Su(z)2*^*−/−*^ cells (Fig. 4D). Notably, in *Psc-Su(z)2*^*−/−*^ cells, ~3543 regions of open chromatin became inaccessible, while about 6196 new regions became accessible, indicating a global shift toward a more relaxed chromatin state in the mutant cells. To assess whether the observed transcriptomic changes were associated with altered chromatin accessibility, we performed an integrative analysis of RNA-seq and ATAC-seq datasets. Approximately 34.7% of genes upregulated in *Psc-Su(z)2*^*−/−*^ cells exhibited significant promoter opening, while 33.1% of downregulated genes were associated with promoter region closure (Fig. 4E; Dataset EV2). Among the downregulated genes with closed promoter regions were key intestinal progenitor markers, such as *esg, Dl*, and *sc*. In contrast, genes with increased promoter accessibility and elevated expression included classical Polycomb targets—such as Hox genes (*Ubx*, *Abd-B*, and *Antp*)—and genes involved in neural development, including *Chinmo* and *Sp1* (Fig. 4E). Additional neural progenitor-related genes, such as *zfh1*, *ct*, *bi*, and *oc*, were also upregulated in the RNA-seq data. These findings indicate a striking possibility: loss of *Psc* and *Su(z)2* may not only lead to a loss of ISC identity but also promote a shift toward a neural progenitor-like state.

To test this hypothesis, we compared the transcriptional profile of *Psc-Su(z)2*^*−/−*^ tumor cells with those of representative intestinal epithelial cell types (*esg*^+^ progenitors, Dl^+^ ISCs, and Pros^+^ EEs), as well as cells from various other *Drosophila* tissues—including the brain and ovary—using data from FlyAtlas 2. Principal component analysis (PCA), based on the top 1000 upregulated genes, revealed that *Psc-Su(z)2*^*−/−*^ tumor cells clustered more closely with neural tissue-derived cells than with gut epithelial cells (Fig. 4F). Together, these results demonstrate that the loss of *Psc* and *Su(z)2* leads to a loss of ISC identity and the aberrant acquisition of neural progenitor-like transcriptional characteristics.

We have previously shown that loss of the transcriptional repressor Tramtrack in ISCs leads to a loss of tissue-specific identity and acquisition of neural stem cell (NSC)-like features, promoting continuous EE production and resulting in the development of neuroendocrine tumors (Li et al, 2020). During this cell identity switch, a set of neuroblast-specific genes—including *dpn, mira, wor, ase*, and *sna*—are upregulated. Among these, *dpn*, in cooperation with the pan-neural gene *seq*, plays a key role in driving tumor progression (Li et al, 2020). In contrast, these classic neuroblast-specific genes are not upregulated in *Psc-Su(z)2* mutant tumor cells. Instead, a distinct subset of genes involved in general neural development is elevated (Fig. 4G). These observations suggest that loss of *Psc* and *Su(z)2* leads to a complete loss of ISC identity without inducing a switch to an NSC-like fate. Rather, the ISCs adopt an abnormal state characterized by the ectopic expression of genes associated with neural progenitor development.

### Transcriptomic screening identifies Chinmo as a key driver of *Psc-Su(z)2* mutant tumorigenesis

**Figure 5:**
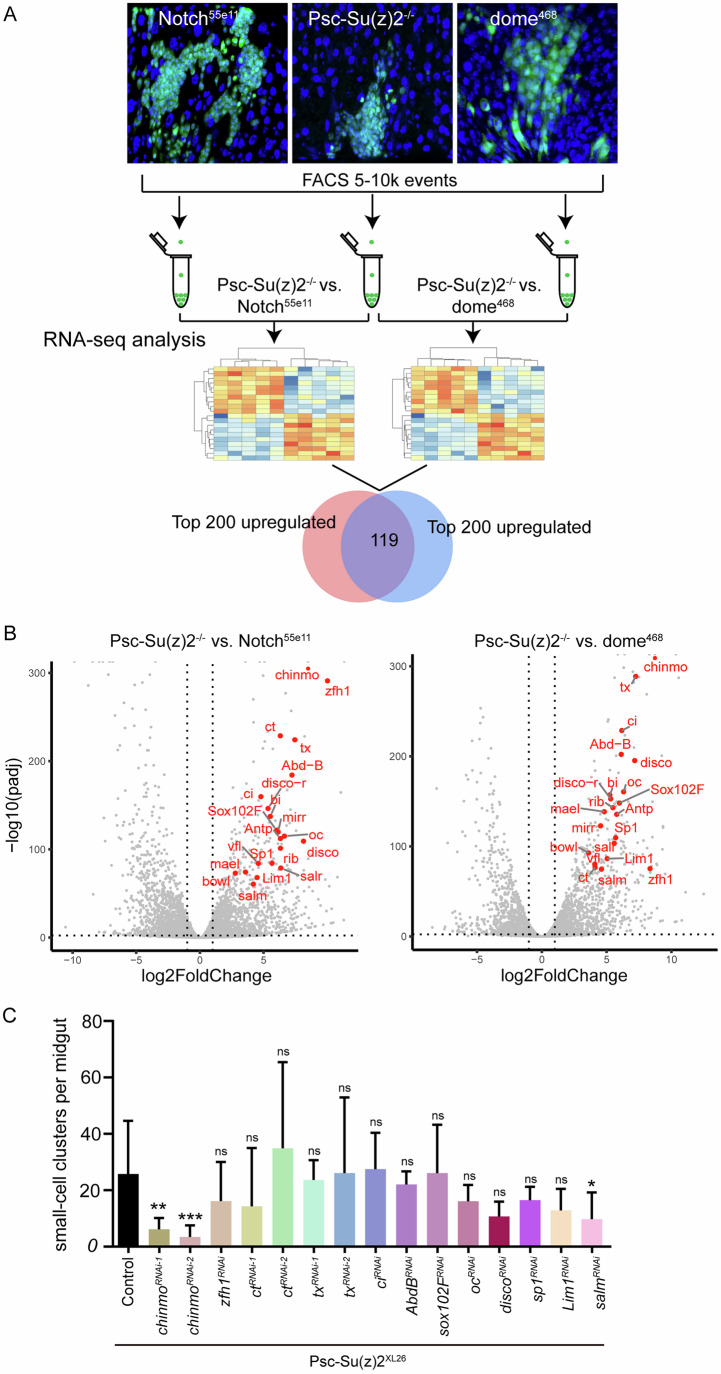
Comparative transcriptome analysis and genetic screens identify genes involved in *Psc-Su(z)2-*driven tumorigenesis. (**A**) A schematic diagram of comparative RNA-seq between *Psc-Su(z)2* deleted MARCM clone cells and two control groups: Notch or dome deleted MARCM clone cells. Both of these groups’ datasets have three biological replicates. (**B**) Volcano plots showing the comparison of gene expression profiles of *Psc-Su(z)2*^*−/−*^ and *Notch*^*55e11*^ cells (left), *Psc-Su(z)2*^*−/−*^ and *dome*^*468*^ cells (right). Red dots depict common upregulated transcription factors of these two comparative pairs in the top 200 upregulated genes. (**C**) Quantification of the number of small-cell clusters per gut in *Psc-Su(z)2*^*−/−*^ flies and *Psc-Su(z)2*^*−/−*^ flies combined with various RNAi lines at 14 days ACI. A total of 6 to 24 guts were examined for each genotype. *P* = 0.005(*chinmo*^*RNAi1*^), *P* = 0.0007(*chinmo*^*RNAi2*^), *P* = 0.0221(*salm*^*RNAi*^). Significance was measured with unpaired two-tailed *t*-tests. Mean ± SEM. ^ns^*p* > 0.05; ^**^p < 0.01; ^***^p < 0.001. Source data are available online for this figure.

To identify genes whose upregulation drives the tumorous outgrowth of *Psc-Su(z)2* mutant cells, we performed a comparative transcriptomic analysis. Since the tumor cells exhibit complete loss of both Notch and JAK/STAT signaling, we used progenitor cells mutant for Notch (*Notch*^*55e11*^) and JAK/STAT (*dome*^*468*^) as separate control groups to exclude genes affected by the loss of these pathways alone. We compared RNA-seq data from *Psc-Su(z)2* mutant tumor cells against each control and selected the top differentially upregulated genes based on statistical significance (Fig. 5A; Dataset EV3).

We then identified the intersection of these two gene sets to isolate genes uniquely upregulated in *Psc-Su(z)2* mutants. Remarkably, more than half overlapped (119 out of 200; Fig. 5A; Dataset EV3), suggesting a common core of *Psc-Su(z)2*-specific transcriptional changes. Among these 119 genes, 21 encoded transcription factors, with 16 of them linked to nervous system development—including *chinmo* and *zfh1* (Fig. 5B). These results indicate that the upregulation of neural development-related genes is specific to the loss of *Psc* and *Su(z)2*, and not a consequence of disrupted Notch or JAK/STAT signaling.

To assess the functional relevance of these transcription factors, we conducted a small-scale RNAi screen. Among the candidates, *chinmo* knockdown significantly reduced tumor incidence in *Psc-Su(z)2* mutant ISC clones (Fig. 5C). Chinmo is a known temporal factor in neural progenitor specification (Maurange, 2020), and its overexpression has been implicated in tumorigenesis (Jiang et al, 2018; Narbonne-Reveau et al, 2016). These findings indicate that Chinmo may be a key effector driving tumorous outgrowth in *Psc-Su(z)2*-deficient ISCs.

### The ectopic activation of *chinmo* mediates tumorous outgrowth of *Psc-Su(z)2* mutant ISCs

**Figure 6:**
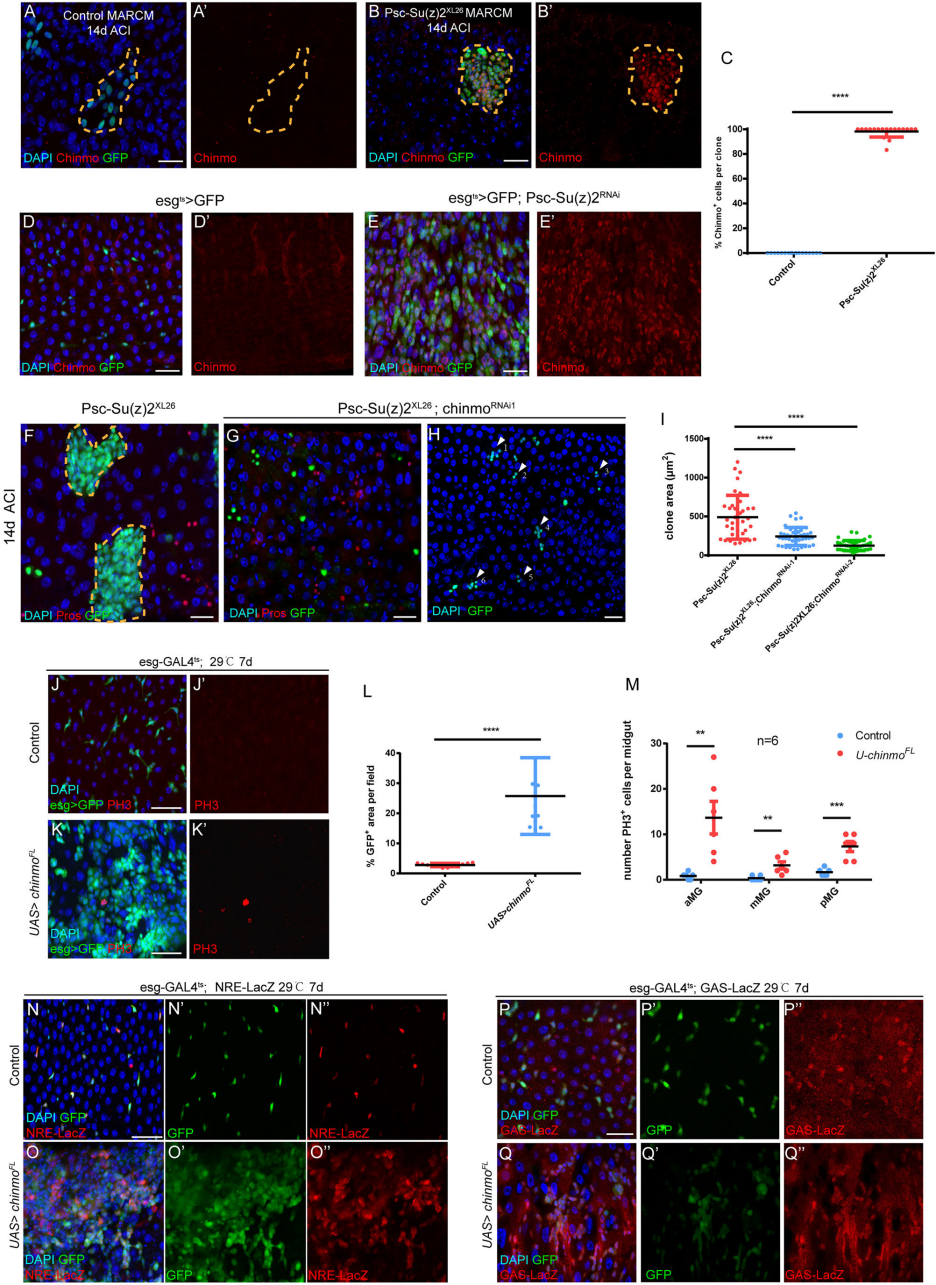
*Chinmo* is required for tumor development from *Psc-Su(z)2* mutant ISCs. (**A**,** B**) MARCM clones marked by GFP co-stained with DAPI (blue) and anti-Chinmo (red) antibody in wild-type *FRT 42D* (**A**) and *Psc-Su(z)2*^*XL26*^ *FRT 42D* (**B**) flies at 14 days ACI. Dashed lines depict the clone margin. ACI, after clone induction. (**C**) Quantification of (**A**, **B**): percentage of Chinmo^+^ cells per clone at 14 days ACI. Data were collected from three independent biological experiments (*N* = 3), with 18 clones from six guts per genotype. *p* < 0.0001. (**D**, **E**) Representative images staining with anti-Chinmo (red) antibody and DAPI (blue) of wild-type *esg*^*ts*^ > *GFP* (**D**) and *esg*^*ts*^*>Psc-Su(z)2*^*RNAi*^ (**E**). Flies were dissected at 7 days after eclosion. (**F**–**H**) MARCM clones co-stained with DAPI (blue) and anti-Pros (red) at 14 days ACI of the following genotypes of flies: *Psc-Su(z)2*^*XL26*^ (**F**), *Psc-Su(z)2*^*XL26*^; *chinmo*^*RNAi1*^ (**G**, **H**). Panel (**H**) shows representative clones of varying sizes upon *chinmo* knockdown in the *Psc-Su(z)2*^*XL26*^ mutant background (#1: 462.77 μm^2^; #2: 90.59 μm^2^; #3: 86.86 μm^2^; #4: 223.79 μm^2^; #5:90.73 μm^2^; #6:327.21 μm^2^). Dashed lines depict the clone margin. (**I**) Quantification of the clone area at 14 days ACI for *Psc-Su(z)2*^*XL26*^, *Psc-Su(z)2*^*XL26*^; *chinmo*^*RNAi1*^, and *Psc-Su(z)2*^*XL26*^; *chinmo*^*RNAi2*^ flies. Data were obtained from three independent biological experiments (*N* = 3), with the following samples analyzed per genotype: *Psc-Su(z)2*^*XL26*^: 37 clones from five guts, *Psc-Su(z)2*^*XL26*^; *chinmo*^*RNAi1*^:41 clones from six guts and *Psc-Su(z)2*^*XL26*^; *chinmo*^*RNAi2*^: 40 clones from five guts. *p* < 0.0001. (**J**,** K**) Representative images staining with anti-PH3 (red) antibody and DAPI (blue) of wild-type *esg*^*ts*^ > *GFP* (**I**) and *esg*^*ts*^*>chinmo*^*FL*^ (**J**) flies. Flies were dissected at 7 days after eclosion. (**L**) Quantification of the percentage of GFP⁺ area relative to total tissue area (% GFP⁺) in a fixed field of view within the midgut R4a region for *esg*^*ts*^>*GFP* and *esg*^*ts*^>*chinmo*^*FL*^ flies. Data were obtained from three independent biological experiments (*N* = 3), with *n* = 12 guts analyzed per genotype. *p* < 0.0001. (**M**) Quantification of the number of PH3^+^ cells along the anterior (aMG), middle (mMG) and posterior (pMG) regions of the midgut. Data were shown for both *esg*^*ts*^ > *GFP* and *esg*^*ts*^*>chinmo*^*FL*^ flies. Data were collected from three independent biological experiments (*N* = 3), with a total of six guts analyzed per genotype. *p* values: aMG: *p* = 0.0051, mMG: *p* = 0.0062, pMG: *p* = 0.0007. (**N**,** O**) Representative images of wild-type *esg*^*ts*^ > *GFP; NRE-LacZ* (**M**) and *esg*^*ts*^ > *GFP; NRE-LacZ; UAS-chinmo*^*FL*^ (**N**) flies. (**P**,** Q**) Representative images staining with LacZ (red) and DAPI (blue) of wild-type *esg*^*ts*^ > *GFP; GAS-LacZ* (**M**) and *esg*^*ts*^ > *GFP; GAS-LacZ; UAS-chinmo*^*FL*^ (**N**) flies. Significance was measured with unpaired two-tailed *t*-tests. Mean ± SEM. ^ns^*p* > 0.05; ^**^p < 0.01; ^***^p < 0.001; ^****^p < 0.0001. Scale bars, 25 μm. See also Figs. EV4 and EV5. Source data are available online for this figure.

We next confirmed the upregulation of Chinmo in *Psc-Su(z)2*^*−/−*^ tumor cells by immunostaining with anti-Chinmo antibodies. Chinmo expression was undetectable in normal *Drosophila* midgut epithelial cells, but was clearly observed in virtually all cells within *Psc-Su(z)2*^*−/−*^ clones (Fig. 6A–C). Ectopic Chinmo expression was also detected in *esg*^*+*^ cells of *esg-GAL4*^*ts*^*, UAS-GFP; UAS-Psc-RNAi, UAS-Su(z)2-RNAi* midguts (Fig. 6D,E). These results indicate that *chinmo* is normally repressed in intestinal progenitor cells but shows abundant expression upon loss of *Psc* and *Su(z)2*.

**Figure EV3:**
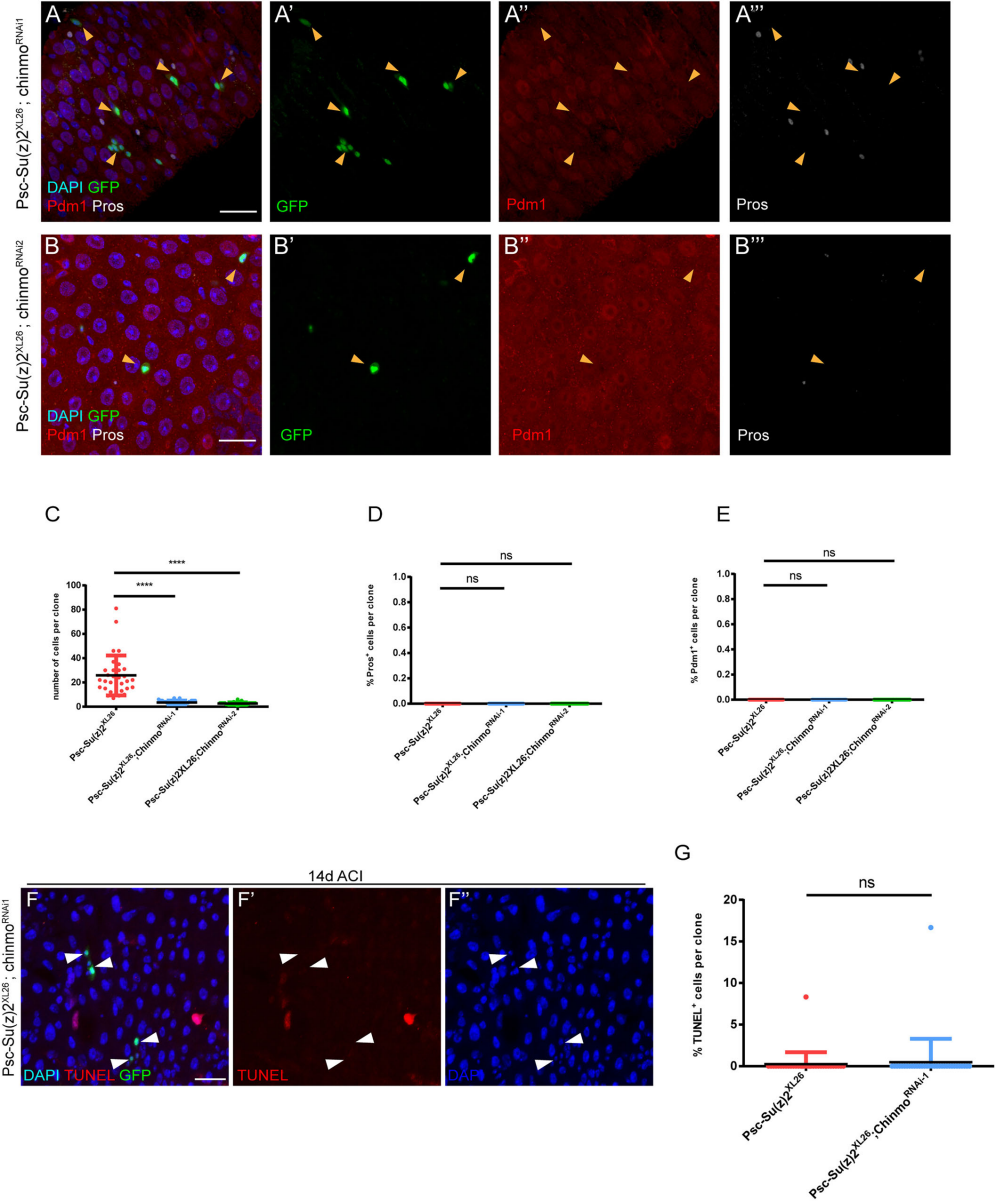
The effect of *chinmo* knockdown on Psc/Su(z)2 mutant clones. (**A**,** B**) MARCM clones marked by GFP and co-stained with DAPI (blue), anti-Pdm1 (red), and anti-Pros (white) antibodies in *Psc-Su(z)2*^*XL26*^; *chinmo*^*RNAi1*^ (A) and *Psc-Su(z)2*^*XL26*^; *chinmo*^*RNAi2*^ (**B**) flies at 14 days ACI. ACI, after clone induction. The yellow arrowheads indicate the *Psc-Su(z)2*^*XL26*^; *chinmo*^*RNAi*^ clones. Note that the mutant clones with *chinmo* RNAi are small, undifferentiated cells. (**C**) Quantification of the number of cells per clone at 14 days ACI for *Psc-Su(z)2*^*XL26*^, *Psc-Su(z)2*^*XL26*^; *chinmo*^*RNAi1*^, and *Psc-Su(z)2*^*XL26*^; *chinmo*^*RNAi2*^ flies. Data were obtained from three independent biological experiments (*N* = 3), with the following samples analyzed per genotype: *Psc-Su(z)2*^*XL26*^: 33 clones from five guts, *Psc-Su(z)2*^*XL26*^; *chinmo*^*RNAi1*^: 33 clones from six guts and *Psc-Su(z)2*^*XL26*^; *chinmo*^*RNAi2*^: 35 clones from five guts. *p* < 0.0001. (**D**,** E**) Quantification of the proportion of Pros^+^ cells (**D**) and Pdm1^+^ cells (**E**) per clone at 14 days ACI for *Psc-Su(z)2*^*XL26*^, *Psc-Su(z)2*^*XL26*^; *chinmo*^*RNAi1*^, and *Psc-Su(z)2*^*XL26*^; *chinmo*^*RNAi2*^ flies. Data were obtained from three independent biological experiments (*N* = 3), with the following samples analyzed per genotype: *Psc-Su(z)2*^*XL26*^: 33 clones from five guts, *Psc-Su(z)2*^*XL26*^; *chinmo*^*RNAi1*^: 33 clones from six guts and *Psc-Su(z)2*^*XL26*^; *chinmo*^*RNAi2*^: 35 clones from five guts. (**F**) MARCM clones marked by GFP co-stained with TUNEL (red) and DAPI (blue) of *Psc-Su(z)2*^*XL26*^; *chinmo*^*RNAi1*^ flies at 14 days ACI. The white arrowheads indicate the *Psc-Su(z)2*^*XL26*^; *chinmo*^*RNAi1*^ cells are negative for TUNEL labeling. (**G**) Quantification of the proportion of TUNEL^+^ cells per clone of *Psc-Su(z)2*^*XL26*^ and *Psc-Su(z)2*^*XL26*^; *chinmo*^*RNAi1*^ at 14 days ACI (*Psc-Su(z)*^*XL26*^: 34 clones from five guts, *Psc-Su(z)2*^*XL26*^; *chinmo*^*RNAi1*^: 34 clones from six guts). Significance was measured with unpaired two-tailed *t*-tests. Mean ± SEM. ^ns^*p* > 0.05; ^**^p < 0.01; ^***^p < 0.001; ^****^p < 0.0001. Scale bars, 25 μm.

To evaluate the functional importance of Chinmo, we expressed *chinmo* RNAi transgenes (RNAi-1: THU2405 and RNAi-2: THU0574) in *Psc-Su(z)2*^*−/−*^ MARCM clones. In both cases, the clones failed to undergo proliferative expansion; most consisted of only 1–4 small diploid cells (Figs. 6F–I and EV3C). This result indicates that Chinmo is essential for the tumorous overgrowth of *Psc-Su(z)2*^*−/−*^ cells. However, staining for Pdm1 and Pros revealed that *Psc-Su(z)2*^−/−^ *chinmo* RNAi clones remained incapable of generating differentiated ECs or EEs (Fig. EV3A,B,D,E), demonstrating that *chinmo* knockdown is insufficient to rescue the differentiation block caused by loss of *Psc* and *Su(z)2*. Importantly, the absence of outgrowth was probably not due to apoptosis, as there were no signs of cell death (Fig. EV3F,G), suggesting that cells instead entered a quiescent state. Taken together, these findings demonstrate that ectopic *chinmo* expression is required for the proliferative tumor phenotype in *Psc-Su(z)2*^*−/−*^ ISCs but is not responsible for the observed differentiation defects.

**Figure EV4:**
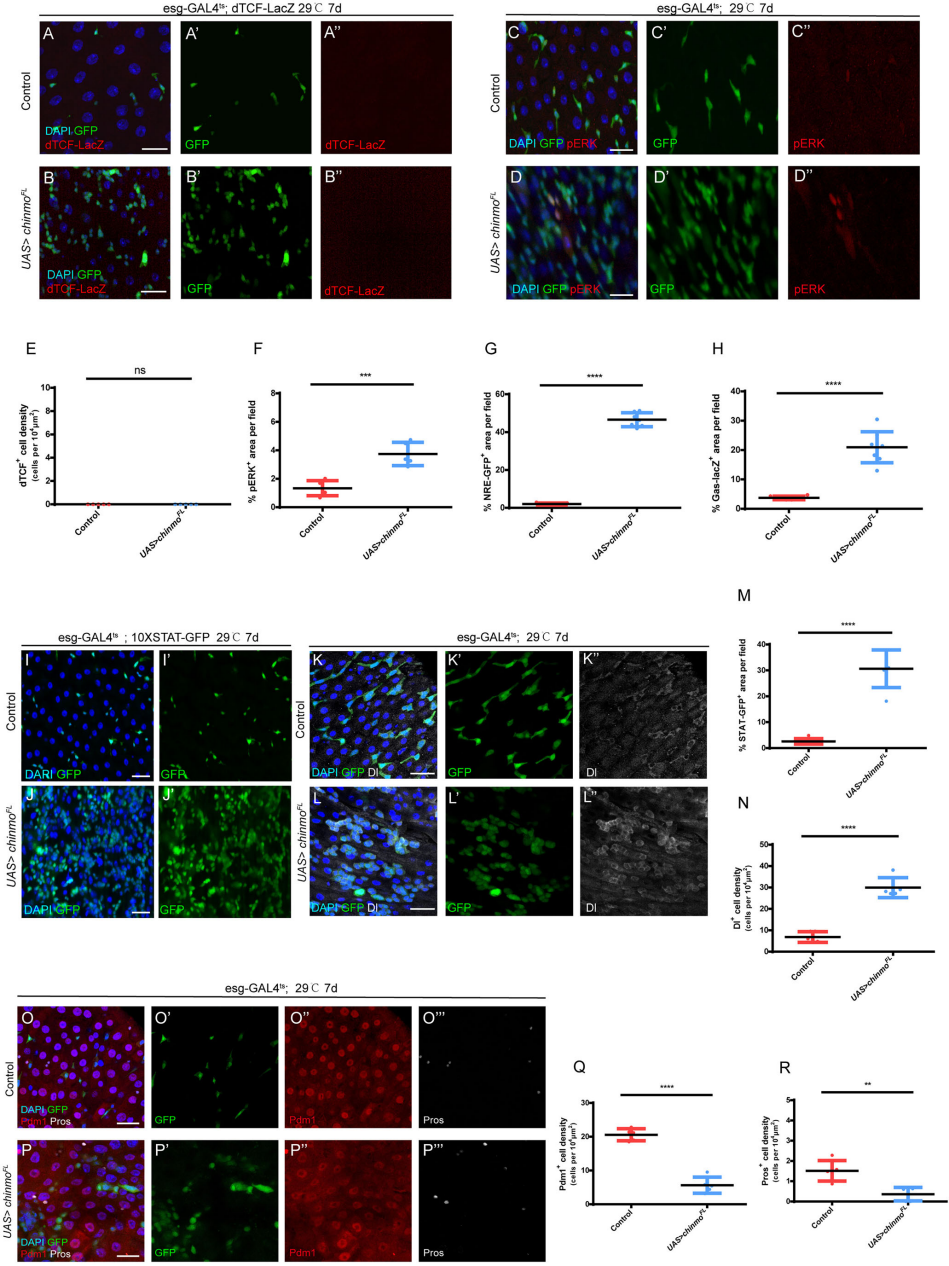
Effects of *chinmo* overexpression on ISC proliferation, differentiation, and JAK/STAT or Wnt signaling pathway activities. (**A**,** B**) Representative images staining with LacZ (red) and DAPI (blue) of wild-type *esg*^*ts*^ > *GFP; dTCF-LacZ* (**A**), and *esg*^*ts*^ > *GFP; dTCF-LacZ; UAS-chinmo*^*FL*^ (**B**) flies. (**C**,** D**) Representative images staining with pERK (red) and DAPI (blue) of wild-type *esg*^*ts*^ > *GFP* (**C**), and *esg*^*ts*^ > *GFP; UAS-chinmo*^*FL*^ (**D**) flies. (**E**) Quantification of the number of dTCF⁺ cells per 10⁴ μm² within the midgut R4a region for *esg*^*ts*^>*GFP* and *esg*^*ts*^>*chinmo*^*FL*^ flies. Data were obtained from three independent biological experiments (*N* = 3), with *n* = 6 guts analyzed per genotype. (**F**) Quantification of the percentage of pERK⁺ area relative to total tissue area (% pERK⁺) in a fixed field of view within the midgut R4a region for *esg*^*ts*^>*GFP* and *esg*^*ts*^>*chinmo*^*FL*^ flies. Data were obtained from three independent biological experiments (*N* = 3), with *n* = 6 guts analyzed per genotype. *P* = 0.0006. (**G**) Quantification of the percentage of NRE-GFP⁺ area relative to total tissue area (% NRE-GFP⁺) in a fixed field of view within the midgut R4a region for *esg*^*ts*^>*GFP* and *esg*^*ts*^>*chinmo*^*FL*^ flies. Data were obtained from three independent biological experiments (*N* = 3), with *n* = 6 guts analyzed per genotype. *p* < 0.0001. (**H**) Quantification of the percentage of Gas-LacZ⁺ area relative to total tissue area (% Gas-LacZ⁺) in a fixed field of view within the midgut R4a region for *esg*^*ts*^>*GFP* and *esg*^*ts*^>*chinmo*^*FL*^ flies. Data were obtained from three independent biological experiments (*N* = 3), with *n* = 6 guts analyzed per genotype. *p* < 0.0001. (**I**,** J**) Representative images staining with DAPI (blue) of wild-type *esg*^*ts*^*-GAL4; 10XSTAT92E-GFP* (**E**) and *esg*^*ts*^*-GAL4; 10XSTAT92E-GFP; UAS-chinmo*^*FL*^ (**F**) flies. (**K**,** L**) Representative images staining with DAPI (blue) and anti-Dl (white) of wild-type *esg*^*ts*^ > *GFP* (**G**), and *esg*^*ts*^ > *GFP; UAS-chinmo*^*FL*^ (**H**) flies. (**M**) Quantification of the percentage of STAT-GFP⁺ area relative to total tissue area (% STAT-GFP⁺) in a fixed field of view within the midgut R4a region for *esg*^*ts*^>*GFP* and *esg*^*ts*^>*chinmo*^*FL*^ flies. Data were obtained from three independent biological experiments (*N* = 3), with *n* = 6 guts analyzed per genotype. *p* < 0.0001. (**N**) Quantification of the number of Dl⁺ cells per 10⁴ μm² within the midgut R4a region for *esg*^*ts*^>*GFP* and *esg*^*ts*^>*chinmo*^*FL*^ flies. Data were obtained from three independent biological experiments (*N* = 3), with *n* = 6 guts analyzed per genotype. *p* < 0.0001. (**O**,** P**) Representative images staining with DAPI (blue), anti-Pdm1 (red) and anti-Pros (white) antibodies of wild-type *esg*^*ts*^ > *GFP* (I), and *esg*^*ts*^ > *GFP; UAS-chinmo*^*FL*^ (**J**) flies. Flies were dissected at 7 days after eclosion. Scale bars, 25 μm. (**Q**) Quantification of the number of Pdm1⁺ cells per 10⁴ μm² within the midgut R4a region for *esg*^*ts*^>*GFP* and *esg*^*ts*^>*chinmo*^*FL*^ flies. Data were obtained from three independent biological experiments (*N* = 3), with *n* = 6 guts analyzed per genotype. *p* < 0.0001. (**R**) Quantification of the number of Pros⁺ cells per 10⁴ μm² within the midgut R4a region for *esg*^*ts*^>*GFP* and *esg*^*ts*^>*chinmo*^*FL*^ flies. Data were obtained from three independent biological experiments (*N* = 3), with *n* = 6 guts analyzed per genotype. *p* = 0.0028. Significance was measured with unpaired two-tailed *t*-tests. Mean ± SEM. ^ns^*p* > 0.05; ^**^p < 0.01; ^***^p < 0.001; ^****^p < 0.0001. Scale bars, 25 μm.

To further investigate the role of *chinmo* in regulating cell proliferation, we generated a *UAS-chinmo* transgene and conditionally expressed it in intestinal progenitor cells using the *esg-GAL4*^*ts*^ driver. Ectopic expression of *chinmo* was sufficient to induce cell proliferation, as indicated by a significant increase in both PH3^+^ mitotic cells and GFP^+^ progenitor cells (Fig. 6J–M). This effect is unlikely to be mediated by EGFR or Wnt signaling, as pERK and dTCF-lacZ levels in *chinmo*-overexpressing ISCs did not show a significant increase (Fig. EV4A–F). The slight upregulation of pERK probably results from mild temperature stress, given that flies were cultured at 29 °C.

Next, we used cell markers and reporter lines to assess whether *chinmo* overexpression affects ISC differentiation by suppressing JAK/STAT and/or Notch signaling pathways. Both pathways remained active, and Dl expression was largely maintained at normal levels (Figs. 6N–Q and EV4G–N). Although the percentage of Pdm1^+^ and Pros^+^ cells appeared reduced in *chinmo*-overexpressing intestines, this decrease is likely a consequence of accelerated ISC proliferation and accumulation of progenitor cells rather than a block in differentiation, as no small cell clusters were observed (Fig. EV4O–R).

Taken together, our findings support a model in which loss of *Psc* and *Su(z)2* in ISCs leads to two parallel consequences: disruption of both Notch and JAK/STAT signaling pathways, resulting in impaired cell differentiation; and derepression of *chinmo*, which drives excessive cell proliferation. These two mechanisms act in concert to promote the tumor-like overgrowth observed in *Psc-Su(z)2*^*−/−*^ ISCs.

### *Psc-Su(z)2* mutant EBs may contribute to tumorigenesis

EBs, the immediate daughter cells of ISCs, are normally post-mitotic and primed for EC commitment (Ohlstein and Spradling, 2007; Wang et al, 2015). However, EBs and ISCs exhibit highly similar transcriptomic and chromatin accessibility landscapes (Guo et al, 2024; Hung et al, 2020). Moreover, under certain stress conditions or upon perturbation of tumor suppressor genes or oncogenes, EBs can re-enter the cell cycle, dedifferentiate into ISCs, and contribute to intestinal repair or tumorigenesis (Tian et al, 2022; Wang et al, 2015). Although our results demonstrate a tumor-suppressive role for *Psc* and *Su(z)2* in ISCs, it remains possible that these genes also function in EBs, and that *Psc-Su(z)2* mutant EBs may be capable of initiating tumor development.

**Figure EV5:**
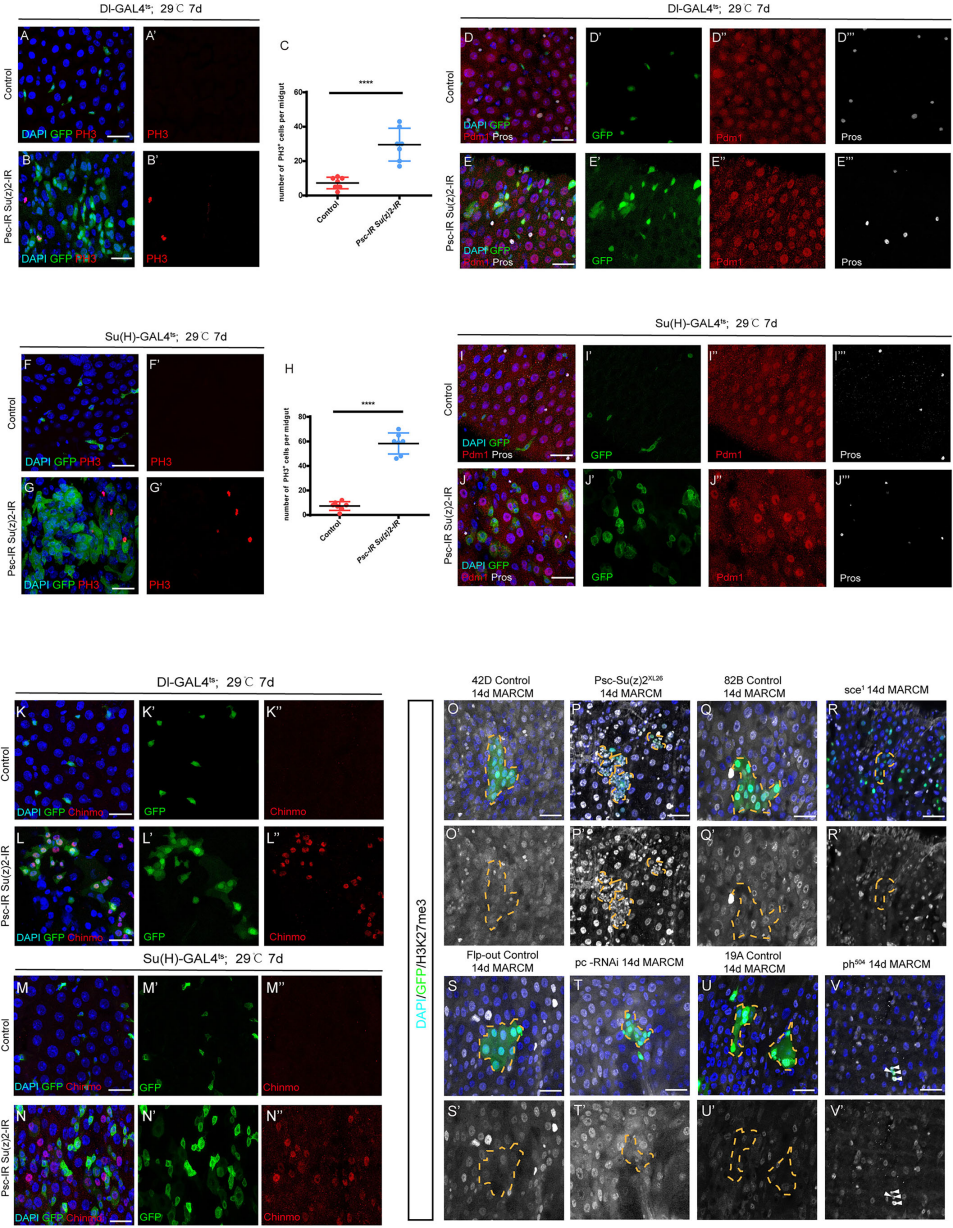
Psc-Su(z)2 may function in EBs as well as ISCs to suppress tumorigenesis, without altering global H3K27me3 levels. (**A**,** B**) Representative images staining with DAPI (blue) and anti-Phospho histone3 (PH3, red) of the following genotypes: *Dl*^*ts*^ > *GFP* control (**A**), and *Dl*^*ts*^ > *GFP; Psc-Su(z)2 RNAi* (**B**). Flies were dissected at 7 days after eclosion. (**C**) Quantification of the number of PH3^+^ cells per midgut related to Fig. EV5A, B. Data were shown for both control and *Psc-Su(z)2 RNAi* flies. Data were obtained from three independent biological experiments (*N* = 3), with *n* = 7 guts analyzed per genotype. *p* < 0.0001. (**D**,** E**) Representative images staining with DAPI (blue), anti-Pdm1 (red) and anti-Pros (white) antibodies of *Dl*^*ts*^ > *GFP* control (**E**) and *Dl*^*ts*^ > *GFP; Psc-Su(z)2 RNAi* (**F**) flies. Flies were dissected at 7 days after eclosion. (**F**,** G**) Representative images staining with DAPI (blue) and anti-Phospho histone3 (PH3, red) of the following genotypes: *Su(H)*^*ts*^ > *GFP* control (**C**), and *Su(H)*^*ts*^ > *GFP; Psc-Su(z)2 RNAi* (**D**). Flies were dissected at 7 days after eclosion. (**H**) Quantification of the number of PH3^+^ cells per midgut related to Fig. EV5F, G. Data were shown for both control and *Psc-Su(z)2 RNAi* flies. Data were obtained from three independent biological experiments (*N* = 3), with *n* = 7 guts analyzed per genotype. *p* < 0.0001. (**I**,** J**) Representative images staining with DAPI (blue), anti-Pdm1 (red) and anti-Pros (white) antibodies of *Su(H)*^*ts*^ > *GFP* control (**G**) and *Su(H)*^*ts*^ > *GFP; Psc-Su(z)2 RNAi* (**H**) flies. Flies were dissected at 7 days after eclosion. (**K**, **L**) Representative images staining with DAPI (blue) and anti-Chinmo (red) antibody of the following genotypes: *Dl*^*ts*^ > *GFP* control (**I**), and *Dl*^*ts*^ > *GFP; Psc-Su(z)2 RNAi* (**J**). Flies were dissected at 7 days after eclosion. (**M**,** N**) Representative images staining with DAPI (blue) and anti-Chinmo (red) antibody of the following genotypes: *Su(H)*^*ts*^ > *GFP* control (**K**), and *Su(H)*^*ts*^ > *GFP; Psc-Su(z)2 RNAi* (L). Flies were dissected at 7 days after eclosion. (**O**–**V**) MARCM clones marked by GFP co-stained with anti-H3K27me3 antibody (white) and DAPI (blue) of the following genotypes: wild-type *FRT 42D* (**A**); *Psc-Su(z)2*^*XL26*^ (**B**), wild-type *FRT 82B* (C), *sce*^*1*^ (**D**), control Flp-out (**E**), *pc*^*RNAi*^ (Flp-out–driven RNAi) (**F**), wild-type *FRT 19 A* (**G**), and *ph*^*504*^(**H**). Dashed lines depict the clone margin. The white arrowheads indicate the *ph*^*504*^ cells. Significance was measured with unpaired two-tailed *t*-tests. Mean ± SEM. ^ns^*p* > 0.05; ^**^p < 0.01; ^***^p < 0.001; ^****^p < 0.0001. Scale bars, 25 μm.

To test this hypothesis, we used lineage-restricted GAL4 drivers to specifically deplete *Psc* and *Su(z)2* in ISCs (Dl-Gal4) and EBs (Su(H)-GBE-Gal4), respectively. As expected, ISC-specific knockdown of *Psc* and *Su(z)2* caused ISC overproliferation and blocked differentiation into either EEs or ECs (Fig. EV5A–E). Interestingly, EB-specific knockdown of *Psc* and *Su(z)2* was sufficient to cause EBs to re-enter the cell cycle and prevent their differentiation into ECs, leading to the accumulation of progenitor cells in the epithelium (Fig. EV5F–J). Notably, Chinmo was also derepressed in EBs upon *Psc* and *Su(z)2* depletion (Fig. EV5K–N), indicating that—similar to what we observed in ISCs—loss of *Psc* and *Su(z)2* in EBs (which may have been present in the previously examined ISC clones) may also contribute to tumorigenesis arising from *Psc-Su(z)2* mutant ISC clones. Interestingly, Chinmo^+^ cells are not always GFP^+^. This indicates that these cells also quickly lose their original cell identity and Notch activity, resulting in decreased GFP expression.

### Mutations in other PRC1 genes do not fully reproduce the tumor phenotype

**Figure 7:**
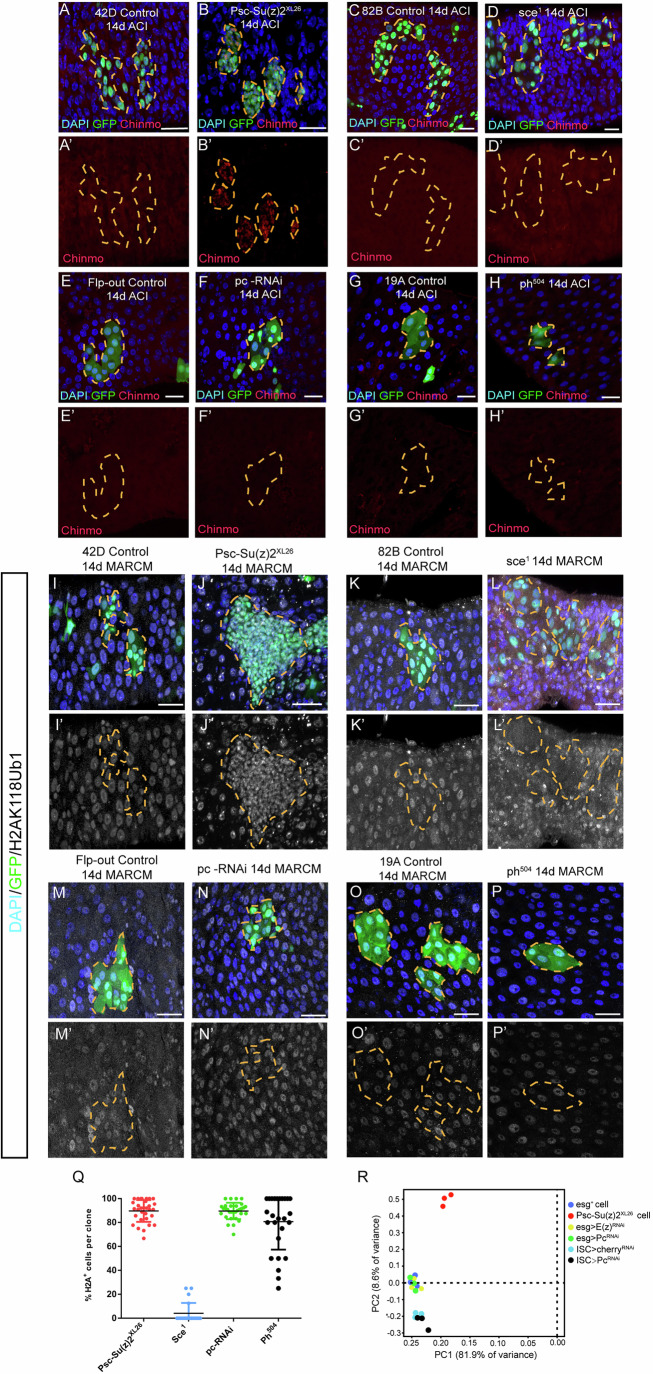
The tumor suppressor function of *Psc-Su(z)2* is independent of the canonical PRC1 function. (**A**–**H**) MARCM clones marked by GFP co-stained with anti-Chinmo antibody (red) and DAPI (blue) of the following genotypes at 14 days ACI: wild-type *FRT 42D* (**A**); *Psc-Su(z)2*^*XL26*^ (**B**), wild-type *FRT 82B* (**C**), *sce*^*1*^ (**D**), control Flp-out (w1118) (**E**), *pc*^*RNAi*^ (Flp-out–driven RNAi) (**F**), wild-type *FRT 19A* (**G**), and *ph*^*504*^(**H**). Dashed lines depict the clone margin. ACI, after clone induction. (**I**–**P**) MARCM clones co-stained with anti-H2AK118Ub1 antibody (white) and DAPI (blue) of the following genotypes: wild-type *FRT 42D* (**I**); *Psc-Su(z)2*^*XL26*^ (**J**), wild-type *FRT 82B* (**K**), *sce*^*1*^ (**L**), control Flp-out (w1118) (**M**), *pc*^*RNAi*^ (Flp-out–driven RNAi) (**N**), wild-type *FRT 19 A* (**O**), and *ph*^*504*^(**P**). Dashed lines depict the clone margin. (**Q**) Quantification of (**J**, **L**, **N**, **P**): the proportion of H2AK118Ub1 (H2A) ^+^ cells per clone in the midgut at 14 days ACI. Data were collected from three independent biological experiments (*N* = 3), with the following samples analyzed per genotype: *Psc-Su(z)2*^*XL26*^ (31 clones from five guts), *sce¹* (25 clones from five guts), *pc*^*RNAi*^ (31 clones from five guts), and *ph⁵⁰⁴* (28 clones from five guts). (**R**) PCA analysis of chromatin accessibility of the following genotypes of cells via ATAC-seq datasets: wild-type esg^+^ cells, *Psc-Su(z)2*^*XL26*^ cells, *esg*^*ts*^*>pc*^*RNAi*^ cells and *esg*^*ts*^ > *e(z)*^*RNAi*^ cells. The ATAC-seq datasets ISC>*cherry*^*RNAi*^ and ISC>*pc*^*RNAi*^ were published by Tauc et al. Significance was measured with unpaired two-tailed *t*-tests. Mean ± SEM. ^ns^*p* > 0.05; ^**^p < 0.01; ^***^p < 0.001; ^****^p < 0.0001. Scale bars, 25 μm. See also Fig. EV5. Source data are available online for this figure.

Since Psc and Su(z)2 are core components of PRC1, we next asked whether mutations in other PRC1 core subunits also lead to tumorous outgrowth in ISCs. Interestingly, mutations in *Sce*, *Pc*, or *ph*, did not produce a tumor-like phenotype. The sizes of *Sce* and *Pc* mutant ISC clones were comparable to wild-type clones, and no accumulation of small diploid cells was observed (Fig. 7A–H). One exception is that, *ph* mutant clones were notably small (Fig. 7H), suggesting that *ph* is required for the proliferation of ISCs.

Consistent with the role of Chinmo in mediating tumorigenesis in *Psc-Su(z)2*^*−/−*^ ISCs, Chinmo expression was not detected in *Sce*, *Pc*, or *ph* mutant clones (Fig. 7A–H). These differing phenotypes suggest that specific PRC1 subunits can have non-overlapping, PRC1-independent functions. In particular, the tumor-suppressive function of *Psc* and *Su(z)2* appears to be at least partially independent of canonical PRC1 activity.

PRC1 is known to catalyze H2AK118ub1, a process dependent on the catalytic core subunit Sce. As expected, loss of *Sce* abolished H2AK118ub1 expression (Fig. 7K,L). Surprisingly, loss of *Psc* and *Su(z)2*, *Pc*, or *ph* did not significantly affect H2AK118ub1 levels in cells within the clones (Fig. 7I–Q). We also examined the expression level of H3K27me3, the catalytic product of PRC2. Consistent with the notion that PRC1 is recruited to chromatin downstream of PRC2, H3K27me3 levels were largely unaffected by *Psc-Su(z)2* deletion, nor by mutations in other PRC1 subunits (Fig. EV5O–EV5V). These results support the notion that *Psc* and *Su(z)2* may function outside the canonical PRC1 or PRC2 complex and independently of H2AK118ub1 to suppress tumor formation.

To generally assess the influence of specific PRC1 components on ISC identity, we performed ATAC-seq on *esg*^*+*^ cells either wild-type or depleted for *Psc-Su(z)2*, *Pc*, or *Sce*, and compared their chromatin accessibility profiles (Fig. 7R). We also included data from *esg*^*+*^ cells with RNAi-mediated knockdown of *E(z)*, a core component of PRC2, and previously published data for *Pc*-depleted *esg*^*+*^ cells (Tauc et al, 2021). PCA analysis revealed that only the loss of *Psc-Su(z)2* led to substantial changes in chromatin accessibility. In contrast, depletion of *Pc*, *Sce*, or *E(z)* had only minimal effects on the chromatin landscape of intestinal progenitor cells (Fig. 7R).

Together, these findings strongly support the conclusion that the tumor-suppressive function of *Psc* and *Su(z)2* operates independently of H2AK118ub1 modification to suppress tumor formation, and suggest distinct, PRC1-independent roles for individual PcG genes in maintaining ISC identity and suppressing tumor formation.

## Discussion

We demonstrate that the two homologous PcG genes, *Psc* and *Su(z)2*, function as tumor suppressors in the adult *Drosophila* midgut by preserving the tissue identity of ISCs. Through transcriptomic and chromatin accessibility analyses, we found that deletion of these genes induces widespread changes in chromatin architecture and gene expression. These alterations result in the transcriptional silencing of ISC identity genes and the ectopic activation of neuronal gene expression programs, leading to tumorigenesis. Interestingly, loss of the transcriptional repressor Tramtrack in ISCs similarly causes genome-wide transcriptional reprogramming, characterized by the downregulation of ISC-specific genes and the upregulation of neuroblast-related genes, including the neuroblast factor Deadpan, which promotes the formation of neuroendocrine tumors (Li et al, 2020). These findings raise the intriguing possibility that ISCs and neural stem cells may share common features in their chromatin landscapes. Such shared features could provide ISCs with the intrinsic capacity to generate enteroendocrine cells—a cell type with neuron-like characteristics. We propose that specific chromatin regulators, such as PcG proteins, act to safeguard this chromatin state and maintain ISC tissue identity. Disruption of this regulatory mechanism may lead to a loss of stem cell identity and the initiation of tumorigenesis.

Notably, our cell-type-specific knockdown analyses further reveal that the tumor-suppressive function of Psc-Su(z)2 is not restricted to ISCs. EB-specific depletion of *Psc-Su(z)2* is sufficient to induce *chinmo* activation, cell cycle re-entry and block differentiation, leading to accumulation of undifferentiated, progenitor-like cells. Thus, both ISCs and their differentiated EB progeny contribute to tumor formation upon loss of *Psc-Su(z)2*, expanding our understanding of the cell origins underlying this tumorigenic program.

The tumor-suppressive function of PcG genes in *Drosophila* has been attributed to diverse mechanisms across different tissues, reflecting their context-dependent roles. For instance, tumorigenesis in imaginal disks has been linked to the activation of Notch and JAK/STAT signaling pathways, in ovarian follicle cells to the activation of Wnt signaling, and in testicular somatic cells to the derepression of a Hox gene. Notably, none of these mechanisms appears to mediate tumor formation in *Psc Su(z)2* mutant ISCs. Instead, the ectopic expression of *Chinmo*, a transcription factor normally undetectable in intestinal epithelial cells, plays a pivotal role in driving tumor growth. Interestingly, Chinmo is also essential for the growth of tumors arising from imaginal disc cells, where its expression is shut down by the Ecdysone–let-7 signaling axis during pupariation, thereby halting tumor progression (Marchetti and Tavosanis, 2017; Narbonne-Reveau and Maurange, 2019). These observations suggest that Chinmo may represent a common oncogenic driver activated in multiple tissues following the loss of PcG gene function. Chinmo is typically expressed in the eye-antennal imaginal disks and testicular cyst stem cells, where its transcription is regulated by JAK/STAT signaling (Flaherty et al, 2010). However, the ectopic expression of *chinmo* in *Psc Su(z)2* mutant ISCs does not depend on JAK/STAT activity, implying that *chinmo* is likely a direct target of PcG-mediated repression. Furthermore, Chinmo is sufficient to recapitulate only the hyperproliferative phenotype of the Psc/Su(z)2 tumor; the differentiation block is likely mediated by additional Psc/Su(z)2-regulated repressors that disrupt the Notch and JAK-STAT signaling.

*chinmo* encodes a BTB–zinc finger transcription factor that was originally identified in *Drosophila* neural progenitor cells, where it regulates temporal neuronal identity during postembryonic brain development (Zhu et al, 2006). Declining gradient of Chinmo protein specifies distinct, birth-order-dependent neuronal fates in extended lineages (Dillard et al, 2018; Liu et al, 2015). In the testis, Chinmo is expressed in cyst stem cells, and its loss results in a “feminization” of these supporting somatic cells, disrupting germline stem cell differentiation and causing male sterility (Flaherty et al, 2010; Grmai et al, 2018; Ma et al, 2014). Although Chinmo lacks a close ortholog in mammals, the human genome encodes approximately 50 BTB–zinc finger transcription factors, several of which are upregulated in cancers, according to the GEPIA cancer database. For example, ZBTB49 is highly expressed in thymic cancer, while ZBTB12 and ZBTB33 show elevated expression in colorectal cancer. These findings raise the intriguing possibility that mammalian BTB–zinc finger transcription factors may be functionally analogous to Chinmo, and it would be of interest to investigate whether these genes are regulated by PcG proteins and whether they contribute to cancer development.

A notable finding from this study is that the tumor-suppressive function of Psc and Su(z)2 appears to be independent of canonical PRC1 activity, including H2AK118ub1. Among the core components of PRC1, only Psc/Su(z)2 depletion in ISCs leads to tumorigenesis. Depletion of Sce, the catalytic subunit responsible for H2AK118ub1, completely abolishes this histone modification on chromatin but does not result in ISC overproliferation or differentiation defects. Similarly, loss of Pc—the chromodomain-containing reader of H3K27me3—has minimal effects in ISCs, with previously reported phenotypes limited to altered cell-fate balance between enterocytes and enteroendocrine cells (Tauc et al, 2021). Consistently, our results show that Pc depletion does not affect ISC proliferation or block differentiation. Loss of Ph seems to result in differentiation defects; however, it also causes reduced ISC proliferation, leading only to small cell clusters (3–5 cells), rather than expansive tumor growth. Importantly, derepression of *chinmo* is not observed following the depletion of Sce, Pc, or Ph, reinforcing the notion that the tumor-suppressive function of Psc and Su(z)2 operates at least partially independently of canonical PRC1. One possibility is that, in the adult stage, certain PRC1-repressed genes become more dependent on Psc/Su(z)2 for their repression, while becoming less dependent on other PRC1 components. Alternatively, Psc and Su(z)2 may function outside of the PRC1 complex—either alone or as part of other protein complexes—to regulate a distinct set of target genes.

The role of PcG genes in maintaining adult stem cell identity has also been documented in various mammalian tissues (Chiacchiera et al, 2016a; Chiacchiera et al, 2016b; Koppens et al, 2016; Pivetti et al, 2019). Moreover, emerging evidence suggests that PcG genes may act as tumor suppressors in specific adult tissues in mammals (Cai et al, 2025; Marchione et al, 2019; Suresh et al, 2016). Our findings further support the notion that specific PcG genes, such as *Psc* and *Su(z)2*, possess tumor-suppressive functions. While finalizing this manuscript, we noticed a preprint that describes a similar tumor-suppressive role for *Psc* and *Su(z)2* in ISCs in adult *Drosophila* (Joly et al, 2025). Given recent reports showing that even transient loss of PcG gene function can result in epigenetic fate reprogramming and tumorigenesis (Parreno et al, 2024), it is critical to deepen our understanding of PcG gene functions in adult tissue homeostasis, and how their dysregulation contributes to cancer development.

## Methods

**Table:** Reagents and tools table

Reagent/resource	Reference or source	Identifier or catalog number
**Experimental models**		
*Psc-Su(z)2* ^*XL26*^	(Li et al, 2010)	N/A
*Su(z)2* ^*1.b8*^	Bloomington Drosophila Stock Center	BDSC: 24467
*Psc* ^*h27*^	Bloomington Drosophila Stock Center	BDSC: 5547
*Su(z)2* ^*1.b7*^	Bloomington Drosophila Stock Center	BDSC: 5572
*ph* ^*504*^	(Beuchle et al, 2001)	N/A
*Sce* ^*1*^	Bloomington Drosophila Stock Center	BDSC: 24618
*N* ^*55e11*^	Bloomington Drosophila Stock Center	BDSC: 28813
*Dome* ^*468*^	(Bier et al, 1989)	N/A
*hs-flp,tub-Gal4, UAS-GFP; FRT 42D, tub-Gal80*	(Lee and Luo, 1999)	N/A
*hs-flp, tub-Gal4,UAS-GFP; FRT 82B, tub-Gal80*	(Lee and Luo, 1999)	N/A
*hs-flp, tub-Gal4, UAS-GFP; FRT 2 A, tub-Gal80*	(Lee and Luo, 1999)	N/A
*hs-flp, FRT 19 A, tub-Gal80; tub-Gal4, UAS-GFP*	(Lee and Luo, 1999)	N/A
*hs-flp;act<stop<Gal4, UAS-GFP*	(Struhl and Basler, 1993)	N/A
*UAS-Su(z)2-RNAi*	Tsinghua Fly Center	THFC: TH04202.N
*UAS-Psc-RNAi*	Tsinghua Fly Center	THFC: THU0177
*UAS-Psc-RNAi;UAS-Su(z)2-RNAi*	(Dietzl et al, 2007)	N/A
*UAS-chinmo-RNAi#1*	Tsinghua Fly Center	THU2405
*UAS-chinmo-RNAi#2*	Tsinghua Fly Center	THU0574
*UAS-chinmo* ^*fl*^	Bloomington Drosophila Stock Center	BDSC: 50740
*UAS-zfh1-RNAi*	Tsinghua Fly Center	THU2529
*UAS-ct-RNAi#1*	Tsinghua Fly Center	THU5930
*UAS-ct-RNAi#2*	Tsinghua Fly Center	THU1309
*UAS-tx-RNAi#1*	Tsinghua Fly Center	TH01948.N
*UAS-tx-RNAi#2*	Tsinghua Fly Center	THU0638
*UAS-ci-RNAi*	Tsinghua Fly Center	TH01945.N
*UAS-AbdB-RNAi*	Vienna Drosophila Resource Center	VDRC: V47
*UAS-Sox102F-RNAi*	Tsinghua Fly Center	TH02009.N
*UAS-oc -RNAi*	Tsinghua Fly Center	THU2524
*UAS-disco-RNAi*	Tsinghua Fly Center	THU4038
*UAS-Sp1 -RNAi*	Tsinghua Fly Center	THU1734
*UAS-Lim1-RNAi*	Tsinghua Fly Center	TH01981.N
*UAS-salm-RNAi*	Tsinghua Fly Center	THU3581
*UAS-EGFR-RNAi*	Tsinghua Fly Center	THU1863
*UAS-dome-RNAi*	Tsinghua Fly Center	THU1049
*UAS-arm-RNAi*	Tsinghua Fly Center	THU1631
*UAS-ttk-RNAi*	Vienna Drosophila Resource Center	VDRC: V10855
*UAS-phyl*	Bloomington Drosophila Stock Center	BDSC: 28102
*NRE-LacZ*	(Zeng et al, 2010)	N/A
*GAS3-LacZ* ^*35.1*^	(Gilbert et al, 2005)	N/A
*dtcf-LacZ*	(Wang et al, 2013)	N/A
*10 x STAT92E-GFP*	Bloomington Drosophila Stock Center	26198
*UAS-Pc-RNAi*	Bloomington Drosophila Stock Center	BDSC: 36070
*UAS-E(z)-RNAi*	Bloomington Drosophila Stock Center	BDSC: 33659
**Antibodies**		
Mouse monoclonal anti-Dl	The Developmental Studies Hybridoma Bank	C594.9B
Mouse monoclonal anti-Pros	The Developmental Studies Hybridoma Bank	MR1A
Rabbit polyclonal anti-Pdm1	Gift from Dr. Xiaohang Yang, Zhejiang University, China	N/A
Rabbit polyclonal anti-phospho-Histone H3	Cell Signaling Technology	9701
Rabbit polyclonal anti-β-galactosidase	MP Biomedicals	0855976
Rabbit polyclonal anti-phospho-ERK	Cell Signaling Technology	4370
Rat polyclonal anti-Chinmo	Gift from Dr. Nicholas Sokol, Indiana University Bloomington, USA	N/A
Rabbit polyclonal anti-Chinmo	(Zhang et al, 2024) Gift from Dr. Qing Ma, Shenzhen Key Laboratory of Synthetic Genomics, China	N/A
Rabbit monoclonal anti-HEAK118Ub1	Cell Signaling Technology	8240
Rabbit polyclonal anti-H3K27me3	Merck	07-449
Goat anti-mouse IgG, Alexa Fluor 568	Invitrogen	A11031
Goat anti-mouse IgG, Alexa Fluor Cy5	Invitrogen	A10524
Goat anti-rabbit IgG, Alexa Fluor 568	Invitrogen	A11036
Goat anti-rabbit IgG, Alexa Fluor Cy5	Invitrogen	A10532
Goat anti-rat IgG, Alexa Fluor 568	Invitrogen	A11077
**Chemicals, enzymes and other reagents**		
Tryptone	Oxoid	LP0042
Yeast extract	Oxoid	LP0021
Agar	Lablead	9002-18-0
DPBS	Gibco	14190250
Triton X-100	Sigma	T9248
Formaldehyde	Sigma	F1635
Methyl alcohol	Sigma	061495
NGS	CST	5425
DAPI	Thermo Fisher	D1306
Hoechst 33342	Invitrogen	H3570
**Software**		
Photoshop 2025	Adobe	
Adobe Illustrator 2025	Adobe	
ImageJ 1.52e	National Institutes of Health	
FlowJo 10	BD Bioscience	
ModfitLT 5	Verity Software House	
Graphpad Prism 6.01	GraphPad Software	
R 3.6.1	https://www.r-project.org/	
Python 3.7	https://www.python.org/	
**Other**		
Leica SP8	Leica	
NIKON A1	Nikon	
ANDOR Dragonfly 200	ANDOR	
BD FACS Aria III-1	BD Biosciences	

### Mosaic analysis

The MARCM system (Lee and Luo, 1999) was frequently used to induce mutations in *Psc-Su(z)2* or other PcG genes in ISCs. Flies were crossed and cultivated at 25 °C. Female flies aged 4–6 days with the desired genotype were collected and heat-shocked in a 37 °C-water bath for 1 h. Following heat shock, the flies were cultivated for another 2 weeks at 25 °C before dissection. The Flp/Out system (Struhl and Basler, 1993) was also used for *Pc*-RNAi clone analysis with clone induction methods similar to those used in the MARCM system.

### GAL4/UAS/GAL80^ts^-mediated gene depletion

The GAL4/UAS binary system and the temperature-sensitive GAL80^ts^ were commonly used to regulate gene expression in specific cell types and developmental stages (Brand and Perrimon, 1993; McGuire et al, 2004). Flies carrying the GAL4/UAS system were crossed and cultivated at 25 °C, and female flies of the desired phenotype aged 7–10 days were dissected for further analysis. Additionally, Flies carrying the temperature-sensitive GAL80^ts^ were crossed and cultivated at 18 °C, and female flies aged 4–6 days with the desired genotype were collected and shifted to 29 °C for 7 days. After that, their midguts were collected and analyzed.

### Genetic screen

The RNAi-based genes knockdown *Drosophila* strains were obtained from the Tsinghua Fly Center and the Vienna Drosophila Resource Center (VDRC), with 2–3 RNAi strains selected for each gene. These RNAi strains were used to construct stocks with *Psc-Su(z)2*^*XL26*^ *FRT 42D* and then crossed with *hs-flp, tubulin* > *GFP; FRT 42D, tublin>GAL80*^*ts*^ strains at 25 °C. The MARCM clones were inducted as described above. After 2 weeks, midguts were collected, and the number of GFP-positive clones per gut was quantified.

### Immunofluorescence staining

The immunofluorescence staining of the fly midgut was performed as previously described (Lin et al, 2008). Briefly, midguts were fixed in 4% formaldehyde for 30 min, and then dehydrated twice in methyl alcohol. After three washes in PBT buffer (PBS with 0.1% Triton X-100), midguts were blocked with 5% NGS solution for 1 h at room temperature. Thereafter, primary antibodies were added to the blocking buffer and incubated overnight at 4 °C. After washing off the nonspecifically bound primary antibodies with PBT buffer three times, the secondary antibodies were added to the PBT buffer and incubated for 2 h at room temperature. Primary and secondary antibodies in this study are listed in the table of antibody resources. Finally, the DAPI staining was used to label the nucleus. Guts were mounted by 70% glycerol and imaged by Nikon A1-R confocal microscope. Images were processed by ImageJ, Adobe Photoshop and compiled in Adobe Illustrator.

### TUNEL labeling

Cell death was detected following the manufacturer’s protocol (In Situ Cell Death Detection Kit, Roche, 1684795). Briefly, midguts were fixed and dehydrated as described above and then incubated in TUNEL reaction mix (enzyme solution: label solution = 1:9) for 1 h at 37 °C. The reaction was stopped by washing the samples with PBT three times.

### FACS

FACS experiments were conducted according to previously published methods (Jin et al, 2020). Female flies with desired genotypes were collected and cultivated at 25 °C. After 4–6 days, the flies were placed in empty tubes (~10 per tube) and treated with 37 °C heat shock for 1 h. The flies were then cultivated at 25 °C for an additional 2 weeks before being dissected. Female flies with the esg^ts^ > GFP genotype were collected and cultivated at 18 °C for 4–6 days year old, and then shifted to 29 °C for 7 days before dissecting. The female flies were placed in pre-cooled DPBS in groups of 100 and dissected within 20 min. Midguts were placed in an EP tube and digested with 1 mg/ml elastase for 1 h at room temperature. The tissue digest was centrifuged at 300×*g* for 10 min, then resuspended in 1 ml DPBS, and the cell suspension was filtered with a 40-μm filter (BD Falcon). After that, 1 mg/ml PI was added to label dead cells. These samples were sorted using FACS Aria II or Aria III (BD Biosciences). The wild-type W1118 midgut cells without any fluorescent labels were used to set the GFP-negative and positive gates. PI^-^GFP^+^ live cells were sorted into an EP tube with 300 μl cold DPBS. Cells were pelleted at 500×*g* for 10 min and processed for subsequent treatments.

Specifically, within ModFit, raw FCS files were imported, DNA content and cell cycle distribution were assessed based on Hoechest staining of GFP+ cells. The “AutoFit” was then applied using the built-in diploid model to deconvolute the cell cycle phases. The software’s automatic calculation of the G0/G1, S, and G2/M percentages was recorded. The fit was accepted only when the reduced chi-square value was below 3, and the visual fit corresponded well with the histogram.

### RNA-seq

Female flies with *hs-flp, tubulin* > *GFP; Psc-Su(z)2*^*XL26*^ *FRT 42D, tublin* > *GAL80*, and *hs-flp, N*^*55e11*^ *FRT 19A, tubulin* > *GAL80; actin* > *GFP*, and *hs-flp, dome*^*468*^ *FRT 19A, tubulin* > *GAL80; actin* > *GFP* and *esg*^*ts*^ > *GFP* genotypes were cultivated and treated following the above measurements. For each sample, ~10,000 midgut epithelial cells were collected using FACS. Subsequently, a series of RNA isolation and amplification kits were employed to obtain the requisite amount of mRNA for RNA-seq. RNA isolation was performed according to the manufacturer’s instructions using the Arcturus PicoPure TM RNA isolation kit (Thermofisher, 12204-1). The RNA was then amplified using the Arcturus RiboAmp PLUS RNA Amplification kit (Thermo Fisher, RA7016). About 10 ng mRNA was enough for library construction. The following steps included reverse transcription of RNA into cDNA, synthesis of cDNA complementary strands, double-stranded DNA purification, in vitro transcription of DNA into aRNA, and aRNA purification, which were performed by the Arcturus cDNA Purification kit (Thermo Fisher, RA7011) and In Vitro Transcription kit (Thermo Fisher, RA7009), respectively. RNA samples were subsequently sequenced by the Illumina HiSeq-2500 system with 50 bp read length. Each genotype has three biological replicates.

### ATAC-seq

Female flies with genotypes of *hs-flp, tubulin* > *GFP; Psc-Su(z)2*^*XL26*^ *FRT 42D, tublin* > *GAL80*, and *esg*^*ts*^ > *GFP*, *esg*^*ts*^ > *Pc*^*RNAi*^, *esg*^*ts*^ > *E(z)*^*RNAi*^ were cultivated and treated following the above measurements. The ATAC-seq was performed using ATAC-Seq Kit (Active Motif, 53150) according to the manufacturer’s instructions. Briefly, approximately 50,000 midgut epithelial cells were collected by FACS. After centrifugation, the pelleted cells were treated with ATAC lysis buffer and subjected to further centrifugation to isolate the nucleus. Then the transposase Tn5 was used to cleave accessible regions on the chromosome and add defined adapters to these DNA fragments. These tagmented DNA were then purified and amplified by PCR to construct a library for next-generation sequencing. The samples were subsequently sequenced by the Illumina HiSeq-2500 system with paired-end 150 bp read length.

### Bioinformatics analysis for RNA-seq data

Quality control for the RNA-seq data were performed using FastQC (v0.11.9) to assess the sequencing quality of both single-end and paired-end reads. The sequencing adapters were removed by Trim galore (0.6.7) and trimmed reads with a length less than 25 bp were also discarded. Quality-controlled reads were then aligned to the Fly genome BDGP6 downloaded from Ensembl with STAR-aligner (2.7.10a). Gene counts were summarized with featureCounts (v2.0.1), and an analysis report was generated with MultiQC (v1.12). DESeq2 (1.26.0) was used to normalize and perform the statistical analysis between sample groups.

### Bioinformatics analysis for ATAC-seq data

The Tn5 adapter was trimmed from both ends of the paired-end reads (2 × 150 bp). Clean reads were mapped to the reference genome by Bowtie2 (2.3.5.1) aligner with the ‘--very-sensitive’ parameter. Duplicate reads were removed using the Picard MarkDuplicates function. Multi-mapped reads and reads in blacklist regions were discarded separately using samtools (v1.7) and bedtools (v2.30.0). Bam files were imported into iDEAR (https://bitbucket.org/juanlmateo/idear/src/master/) to identify positively and negatively enriched regions relative to the control sample. Significantly changed regions were annotated using annotatePeaks.pl function from Homer.

### Quantification and statistical analysis

For counting assays of enterocytes, ImageJ was used to count these polyploid cells according to the significantly larger diameters of their nucleus. Statistical analysis and graphs construction were performed using Graphpad Prism6. Each experimental dataset included more than three biological replicates for analysis. All significance tests were analyzed by *t*-tests, mean ± SEM. Significance *P* values: ^ns^*p* > 0.05, ^∗^*p* < 0.05, ^∗∗^*p* < 0.01, ^∗∗∗^*p* < 0.001, and ^∗∗∗∗^*p* < 0.0001.

## Supplementary information

Peer Review File

Dataset EV1

Dataset EV2

Dataset EV3

Source data Fig. 1

Source data Fig. 2

Source data Fig. 3

Source data Fig. 4

Source data Fig. 5

Source data Fig. 6

Source data Fig. 7

Expanded View Figures

## Data Availability

The datasets produced in this study are available at the Gene Expression Omnibus with the accession code GSE299390: https://www.ncbi.nlm.nih.gov/geo/query/acc.cgi?acc=GSE299390. The source data of this paper are collected in the following database record: biostudies:S-SCDT-10_1038-S44319-026-00837-x.
