# Supplementary material for: Psc and Su(z)2 safeguard intestinal stem cell identity and prevent chinmo-dependent tumorigenesis
Source: EMBO Rep. 2026 Jun 20;27(15):4433–61. doi: 10.1038/s44319-026-00837-x (PMC13458361; doi:10.1038/s44319-026-00837-x)
Supplement: Supplementary file 1 [file 44319_2026_837_MOESM12_ESM.pdf]

Expanded View Figures

Figure EV1. Analysis of *Psc-Su(z)2* mutant clones with or without transgene expression.

(**A**) Quantification of the clone area at 14 days ACI for wild-type *FRT 42D*, $Su(z)2^{RNAi}$ *FRT 82B*, Psc^{h27} *FRT 42D*, $Psc\text{-}Su(z)2^{XL26}$ and $Su(z)2^{1.b8}$. Data were obtained from three independent biological experiments ($N = 3$), with the following samples analyzed per genotype: wild-type *FRT 42D*: 77 clones from 5 guts; $Su(z)2^{RNAi}$ *FRT 82B:* 40 clones from 5 guts; psc^{h27} *FRT 42D:* 41 clones from 5 guts; $Psc\text{-}Su(z)2^{XL26}$: 79 clones from 5 guts; $Su(z)2^{1.b8}$: 34 clones from 5 guts. ACI, after clone induction. *p* values: $Psc\text{-}Su(z)2^{XL26}$: $p = 0.0008$, $Su(z)2^{1.b8}$: $p = 0.0055$. (**B**) Quantification of clone cell density (number of cells per clone area) at 14 days ACI for wild-type *FRT 42D*, $Su(z)2^{RNAi}$ *FRT 82B*, Psc^{h27} *FRT 42D*, $Psc\text{-}Su(z)2^{XL26,}$ and $Su(z)2^{1.b8}$. Data were obtained from three independent biological experiments ($N = 3$), with the following samples analyzed per genotype: wild-type *FRT 42D*: 77 clones from five guts; $Su(z)2^{RNAi}$ *FRT 82B:* 40 clones from five guts; Psc^{h27} *FRT 42D:* 41 clones from five guts; $Psc\text{-}Su(z)2^{XL26}$: 79 clones from five guts; $Su(z)2^{1.b8}$: 34 clones from five guts. ACI, after clone induction. *p* values: $Psc\text{-}Su(z)2^{XL26}$: $p < 0.0001$, $Su(z)2^{1.b8}$: $p < 0.0001$. (**C**) Quantification of the number of clones at 14 days ACI along the anterior (aMG), middle (mMG) and posterior (pMG) regions of the midgut in $Psc\text{-}Su(z)2^{XL26}$ flies. Data were obtained from three independent biological experiments ($N = 3$), with $n = 13$ guts analyzed per genotype. (**D**) Quantification of the $PH3^+$ cell number per clone at 14 days ACI of wild-type *FRT 42D* and $Psc\text{-}Su(z)2^{XL26}$ flies. Data were obtained from three independent biological experiments ($N = 3$), with the following samples analyzed per genotype: control 14 d ACI: 205 clones from six guts, $Psc\text{-}Su(z)2^{XL26}$ 14 d ACI: 155 clones from six guts. ACI, after clone induction. $p < 0.0001$. (**E**) Quantification for the percentage of clones containing $PH3^+$ cells per midgut. Data were obtained from three independent biological experiments ($N = 3$), with the following samples analyzed per genotype: wild-type 14 d ACI: ten clones from five guts; $Psc\text{-}Su(z)2^{XL26}$ 14 d ACI: 11 clones from five guts. ACI, after clone induction. $p = 0.0053$. Note: In this set of experiments, ISCs in both control and experimental flies exhibited evidently elevated baseline activity. This is likely attributable to unintended midgut stress during the 14-day room temperature incubation after heat shock. Data obtained under physiological conditions are presented in Fig. 1F. (**F**, **G**) MARCM clones marked by GFP and co-stained with DAPI (blue), anti-Pdm1 (red) and anti-Pros (white) antibodies in $Psc\text{-}Su(z)2^{XL26}$; *UAS-Su(z)2 FRT 42D* (D) and $Psc\text{-}Su(z)2^{XL26}$; *UAS-Psc FRT 42D* (E) flies at 14 days ACI. Dashed lines depict the clone margin. (**H**) Quantification of the clone area in wild-type *FRT 42D*, $Psc\text{-}Su(z)2^{XL26}$; *UAS-Psc FRT 42D* and $Psc\text{-}Su(z)2^{XL26}$; *UAS-Su(z)2 FRT 42D* clones at 14 days ACI. Data were obtained from three independent biological experiments ($N = 3$), with the following samples analyzed per genotype: control: 34 clones from five guts, $Psc\text{-}Su(z)2^{XL26}$; *UAS-Psc FRT 42D*: 35 clones from 5 guts, $Psc\text{-}Su(z)2^{XL26}$; *UAS-Su(z)2 FRT 42D*: 34 clones from five guts. ACI after clone induction. *p* values: $Psc\text{-}Su(z)2^{XL26}$; *UAS-Psc FRT 42D*: $p = 0.0001$; $Psc\text{-}Su(z)2^{XL26}$; *UAS-Su(z)2 FRT 42D*: $p < 0.0001$. (**I**, **J**) Quantification of the proportion of $Pdm1^+$ cells (**G**) and $Pros^+$ cells (**H**) per clone in 14-day clones of wild-type *FRT 42D*, $Psc\text{-}Su(z)2^{XL26}$; *UAS-Psc FRT 42D* and $Psc\text{-}Su(z)2^{XL26}$; *UAS-Su(z)2 FRT 42D* flies. Data were obtained from three independent biological experiments ($N = 3$), with the following samples analyzed per genotype: control: 26 clones from six guts, $Psc\text{-}Su(z)2^{XL26}$; *UAS-Psc FRT 42D*: 26 clones from 6 guts, $Psc\text{-}Su(z)2^{XL26}$; *UAS-Su(z)2 FRT 42D*: 28 clones from five guts. Significance was measured with unpaired two-tailed *t*-tests. Mean ± SEM. $^{ns}p > 0.05$; $^{**}p < 0.01$; $^{***}p < 0.001$; $^{****}p < 0.0001$. Scale bars, 25 µm.

 © The Author(s)

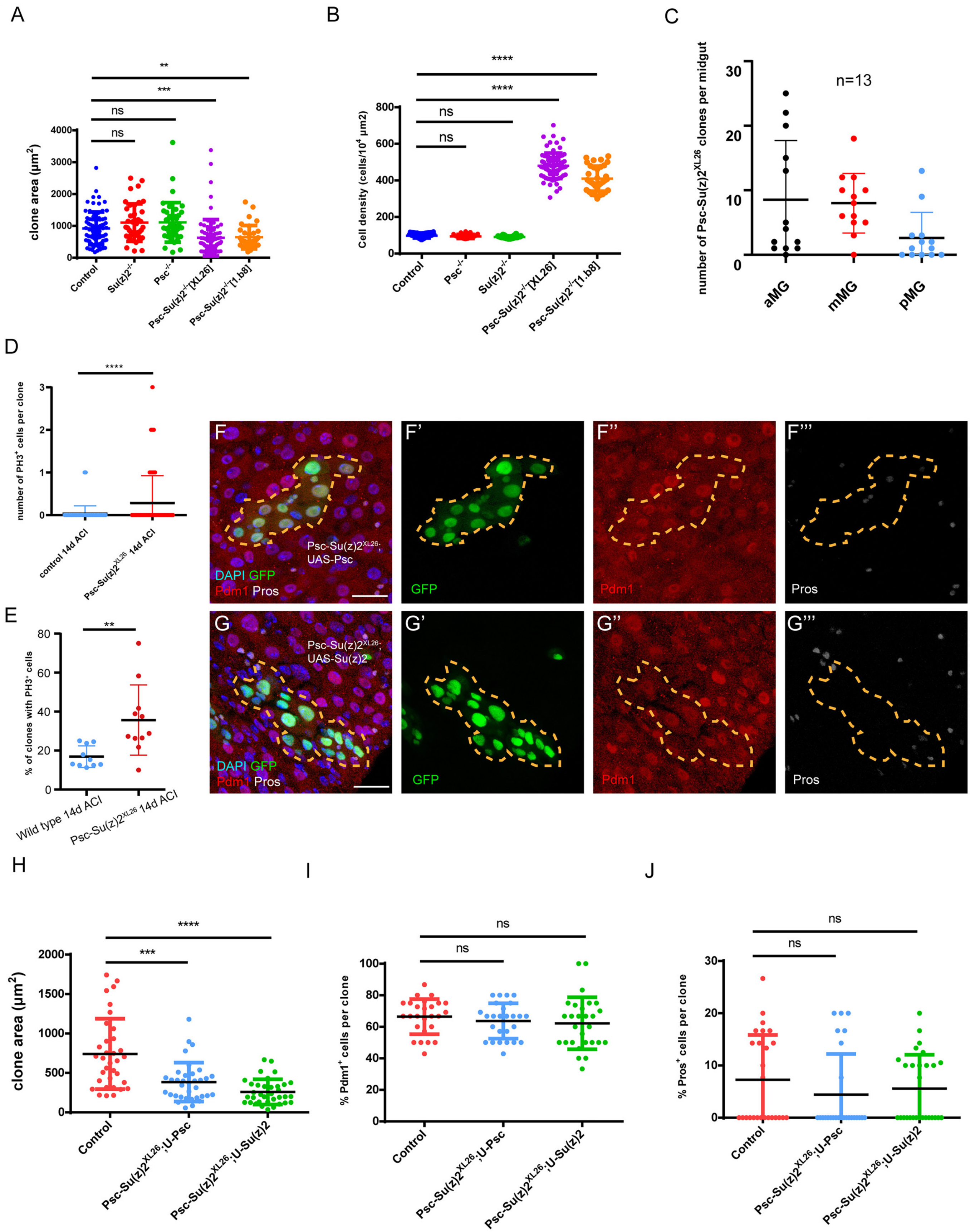
A
clone area (μm²)
Control
Su(z)2-/-
Psc-/-
Psc-Su(z)2-/-[XL26]
Psc-Su(z)2-/-[1.b8]
ns
ns
***
**
B
Cell density (cells/10⁴ μm2)
Control
Psc-/-
Su(z)2-/-
Psc-Su(z)2-/-[XL26]
Psc-Su(z)2-/-[1.b8]
ns
ns
****
****
C
number of Psc-Su(z)2XL26 clones per midgut
n=13
aMG
mMG
pMG
D
number of PH3+ cells per clone
****
control 14d ACI
Psc-Su(z)2XL26 14d ACI
E
% of clones with PH3+ cells
**
Wild type 14d ACI
Psc-Su(z)2XL26 14d ACI
F
Psc-Su(z)2XL26; UAS-Psc
DAPI GFP
Pdm1 Pros
F'
GFP
F''
Pdm1
F'''
Pros
G
Psc-Su(z)2XL26; UAS-Su(z)2
DAPI GFP
Pdm1 Pros
G'
GFP
G''
Pdm1
G'''
Pros
H
clone area (μm²)
***
****
Control
Psc-Su(z)2XL26;U-Psc
Psc-Su(z)2XL26;U-Su(z)2
I
% Pdm1+ cells per clone
ns
ns
Control
Psc-Su(z)2XL26;U-Psc
Psc-Su(z)2XL26;U-Su(z)2
J
% Pros+ cells per clone
ns
ns
Control
Psc-Su(z)2XL26;U-Psc
Psc-Su(z)2XL26;U-Su(z)2

© The Author(s)

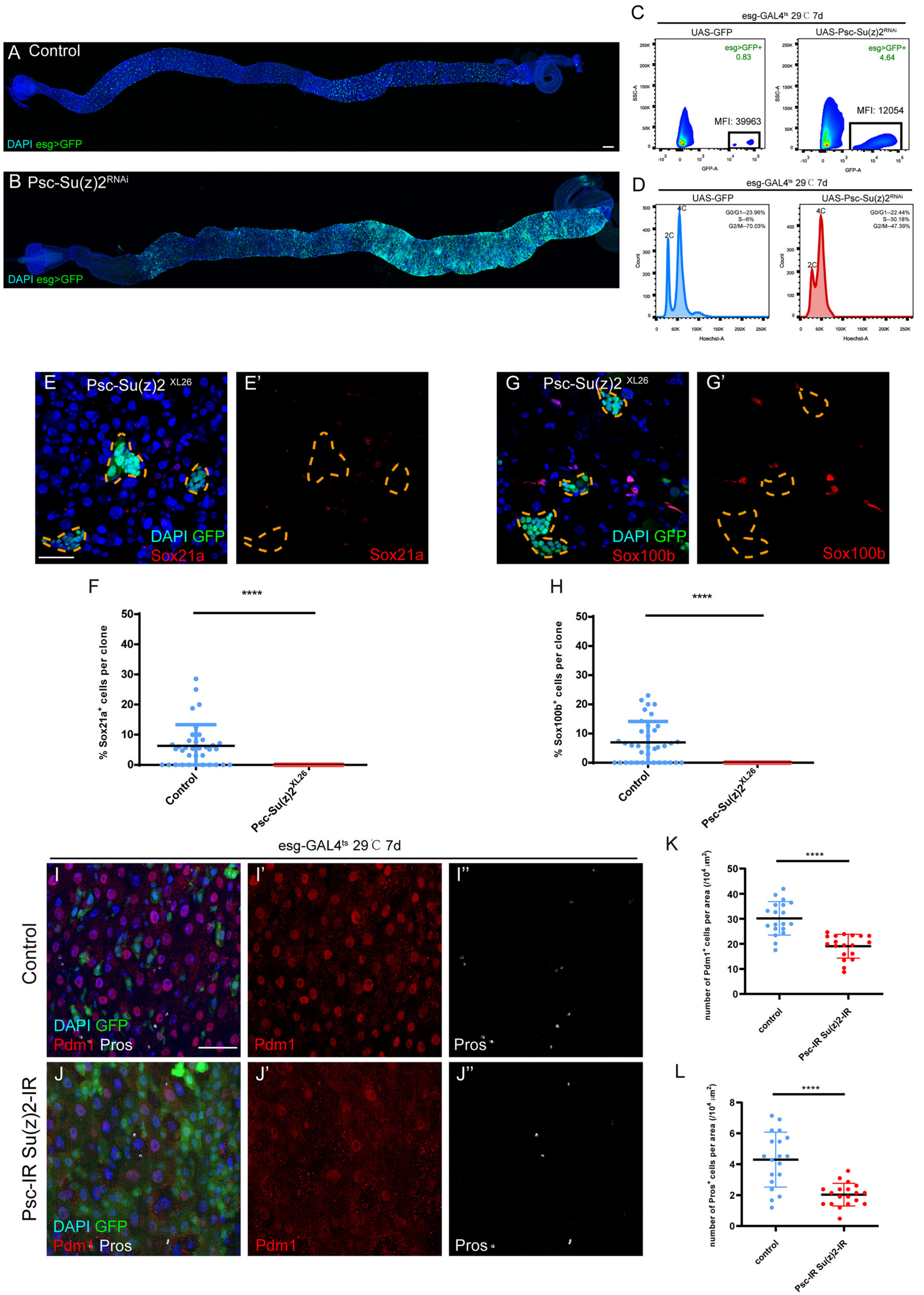
A Control
DAPI esg>GFP
B Psc-Su(z)2RNAi
DAPI esg>GFP
C
esg-GAL4ts 29℃ 7d
UAS-GFP
UAS-Psc-Su(z)2RNAi
esg>GFP+ 0.83
esg>GFP+ 4.64
MFI: 39963
MFI: 12054
SSC-A
GFP-A
D
esg-GAL4ts 29℃ 7d
UAS-GFP
UAS-Psc-Su(z)2RNAi
G0/G1--23.96% S--6% G2/M--70.03%
G0/G1--22.44% S--30.18% G2/M--47.39%
2C
4C
Count
Hoechst-A
E Psc-Su(z)2 XL26
DAPI GFP
Sox21a
E'
Sox21a
G Psc-Su(z)2 XL26
DAPI GFP
Sox100b
G'
Sox100b
F
****
% Sox21a+ cells per clone
Control
Psc-Su(z)2XL26
H
****
% Sox100b+ cells per clone
Control
Psc-Su(z)2XL26
esg-GAL4ts 29℃ 7d
Control
Psc-IR Su(z)2-IR
I
I'
I''
DAPI GFP
Pdm1 Pros
Pdm1
Pros
J
J'
J''
DAPI GFP
Pdm1 Pros
Pdm1
Pros
K
****
number of Pdm1+ cells per area (/10⁴ μm²)
control
Psc-IR Su(z)2-IR
L
****
number of Pros+ cells per area (/10⁴ μm²)
control
Psc-IR Su(z)2-IR

 © The Author(s)

Figure EV2. Tumor cells in *Psc-Su(z)2* mutants are diploid and highly proliferative.

(**A**, **B**) Representative images of the whole gut of the following genotypes of flies stained with DAPI (blue): wild-type *esg*^{ts} > *GFP* (**A**) and *esg*^{ts}>*Psc-Su(z)2*^{RNAi} (**B**). (**C**) FACS analysis of the proportion of *esg* > *GFP* cells in guts of *esg*^{ts} > *GFP* and *esg*^{ts}>*Psc-Su(z)2*^{RNAi} flies. (**D**) FACS analysis of the cell cycle of *esg*^{ts} > *GFP* and *esg*^{ts}>*Psc-Su(z)2*^{RNAi} cells. (**E**) MARCM clones marked by GFP co-stained with DAPI (blue) and anti-Sox21a (red) in *Psc-Su(z)2*^{XL26} *FRT 42D* flies at 14 days ACI. Dashed lines depict the clone margin. ACI, after clone induction. (**F**) Quantification of the percentage of Sox21a$^+$ cells per clone in 14-day clones of wild-type *FRT 42D* and *Psc-Su(z)2*^{XL26} flies. Data were obtained from three independent biological experiments ($N = 3$), with the following samples analyzed per genotype: wild-type *FRT 42D*: 36 clones from six guts and *Psc-Su(z)2*^{XL26}: 39 clones from six guts. $p < 0.0001$. (**G**) MARCM clones co-stained with DAPI (blue) and anti-Sox100b (red) in *Psc-Su(z)2*^{XL26} *FRT 42D* flies at 14 days ACI. Dashed lines depict the clone margin. (**H**) Quantification of the percentage of Sox100b$^+$ cells per clone in 14-day clones of wild-type *FRT 42D* and *Psc-Su(z)2*^{XL26} flies. Data were obtained from three independent biological experiments ($N = 3$), with the following samples analyzed per genotype: wild-type *FRT 42D*: 37 clones from six guts and *Psc-Su(z)2*^{XL26}: 39 clones from six guts. $p < 0.0001$. (**I**, **J**) Representative images staining with DAPI (blue), anti-Pdm1 (red) and anti-Pros (white) antibodies of *esg*^{ts} > *GFP* control (**I**) and *esg*^{ts} > *GFP; Psc-Su(z)2 RNAi* (**J**) flies. Flies were dissected at 7 days after eclosion. (**K**, **L**) Quantification of (**I**, **J**): numbers of Pdm1$^+$ cells (**K**) and Pros$^+$ cells (**L**) per area (10^4 μm^2) in the midgut at 14 days ACI. Data were obtained from three independent biological experiments ($N = 3$), with 19 areas from five guts per genotype. ACI, after clone induction. Significance was measured with unpaired two-tailed *t*-tests. $p < 0.0001$. Mean ± SEM. $^{ns}p > 0.05$; $^{**}p < 0.01$; $^{***}p < 0.001$; $^{****}p < 0.0001$. Scale bars, 25 μm.

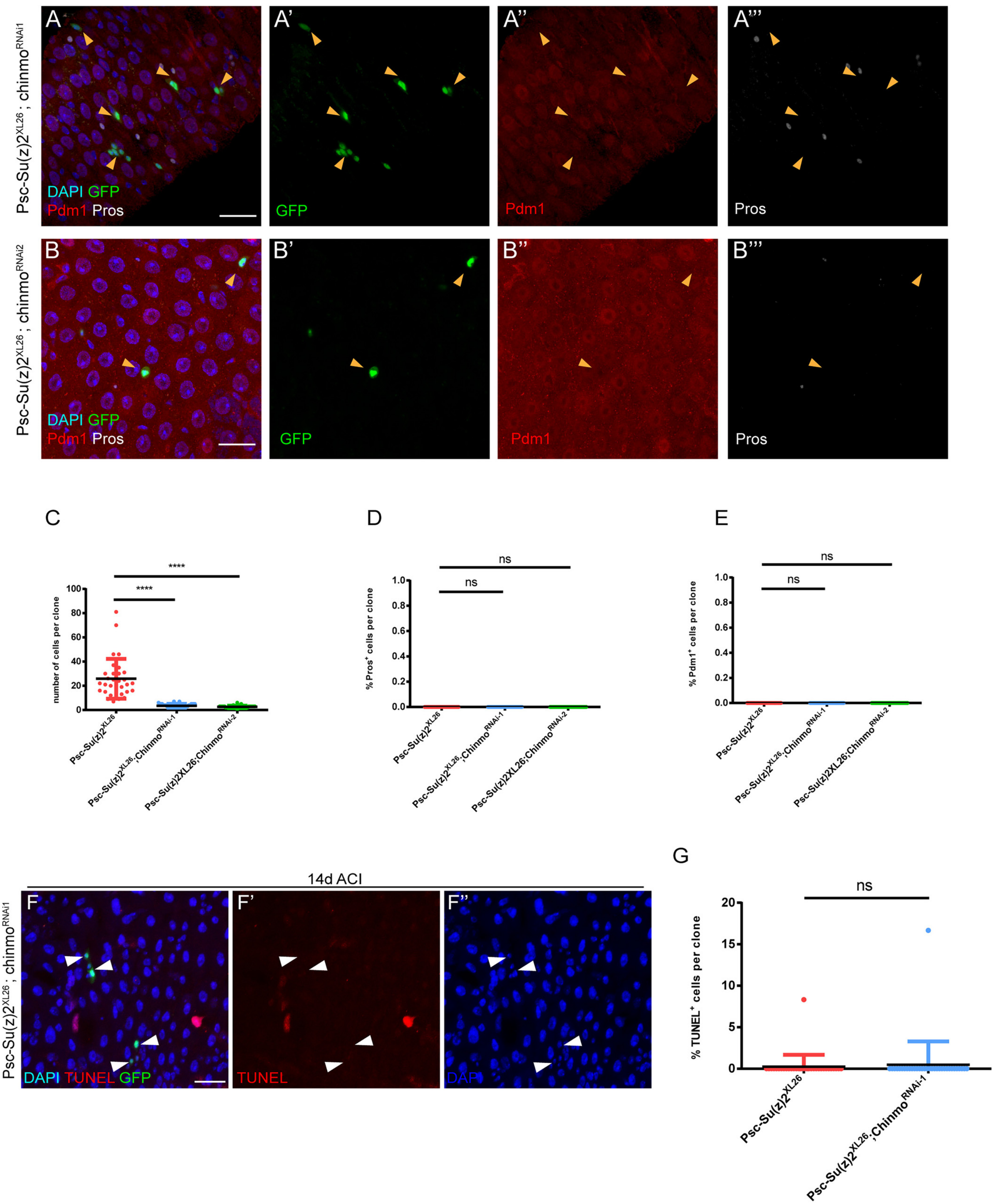
Psc-Su(z)2XL26 ; chinmoRNAi1
A
DAPI GFP
Pdm1 Pros
A'
GFP
A''
Pdm1
A'''
Pros
Psc-Su(z)2XL26 ; chinmoRNAi2
B
DAPI GFP
Pdm1 Pros
B'
GFP
B''
Pdm1
B'''
Pros
C
****
****
number of cells per clone
100
80
60
40
20
0
Psc-Su(z)2XL26
Psc-Su(z)2XL26;ChinmoRNAi-1
Psc-Su(z)2XL26;ChinmoRNAi-2
D
ns
ns
% Pros+ cells per clone
1.0
0.8
0.6
0.4
0.2
0.0
Psc-Su(z)2XL26
Psc-Su(z)2XL26;ChinmoRNAi-1
Psc-Su(z)2XL26;ChinmoRNAi-2
E
ns
ns
% Pdm1+ cells per clone
1.0
0.8
0.6
0.4
0.2
0.0
Psc-Su(z)2XL26
Psc-Su(z)2XL26;ChinmoRNAi-1
Psc-Su(z)2XL26;ChinmoRNAi-2
14d ACI
Psc-Su(z)2XL26 ; chinmoRNAi1
F
DAPI TUNEL GFP
F'
TUNEL
F''
DAPI
G
ns
% TUNEL+ cells per clone
20
15
10
5
0
Psc-Su(z)2XL26
Psc-Su(z)2XL26;ChinmoRNAi-1

 © The Author(s)

Figure EV3. The effect of *chinmo* knockdown on Psc/Su(z)2 mutant clones.

(**A**, **B**) MARCM clones marked by GFP and co-stained with DAPI (blue), anti-Pdm1 (red), and anti-Pros (white) antibodies in *Psc-Su(z)2^{XL26}*; *chinmo^{RNAi1}* (A) and *Psc-Su(z)2^{XL26}*; *chinmo^{RNAi2}* (**B**) flies at 14 days ACI. ACI, after clone induction. The yellow arrowheads indicate the *Psc-Su(z)2^{XL26}*; *chinmo^{RNAi}* clones. Note that the mutant clones with *chinmo* RNAi are small, undifferentiated cells. (**C**) Quantification of the number of cells per clone at 14 days ACI for *Psc-Su(z)2^{XL26}*, *Psc-Su(z)2^{XL26}*; *chinmo^{RNAi1}*, and *Psc-Su(z)2^{XL26}*; *chinmo^{RNAi2}* flies. Data were obtained from three independent biological experiments ($N = 3$), with the following samples analyzed per genotype: *Psc-Su(z)2^{XL26}*: 33 clones from five guts, *Psc-Su(z)2^{XL26}*; *chinmo^{RNAi1}*: 33 clones from six guts and *Psc-Su(z)2^{XL26}*; *chinmo^{RNAi2}*: 35 clones from five guts. $p < 0.0001$. (**D**, **E**) Quantification of the proportion of Pros$^+$ cells (**D**) and Pdm1$^+$ cells (**E**) per clone at 14 days ACI for *Psc-Su(z)2^{XL26}*, *Psc-Su(z)2^{XL26}*; *chinmo^{RNAi1}*, and *Psc-Su(z)2^{XL26}*; *chinmo^{RNAi2}* flies. Data were obtained from three independent biological experiments ($N = 3$), with the following samples analyzed per genotype: *Psc-Su(z)2^{XL26}*: 33 clones from five guts, *Psc-Su(z)2^{XL26}*; *chinmo^{RNAi1}*: 33 clones from six guts and *Psc-Su(z)2^{XL26}*; *chinmo^{RNAi2}*: 35 clones from five guts. (**F**) MARCM clones marked by GFP co-stained with TUNEL (red) and DAPI (blue) of *Psc-Su(z)2^{XL26}*; *chinmo^{RNAi1}* flies at 14 days ACI. The white arrowheads indicate the *Psc-Su(z)2^{XL26}*; *chinmo^{RNAi1}* cells are negative for TUNEL labeling. (**G**) Quantification of the proportion of TUNEL$^+$ cells per clone of *Psc-Su(z)2^{XL26}* and *Psc-Su(z)2^{XL26}*; *chinmo^{RNAi1}* at 14 days ACI (*Psc-Su(z)^{XL26}*: 34 clones from five guts, *Psc-Su(z)2^{XL26}*; *chinmo^{RNAi1}*: 34 clones from six guts). Significance was measured with unpaired two-tailed *t*-tests. Mean ± SEM. $^{ns}p > 0.05$; $^{**}p < 0.01$; $^{***}p < 0.001$; $^{****}p < 0.0001$. Scale bars, 25 µm.

© The Author(s)

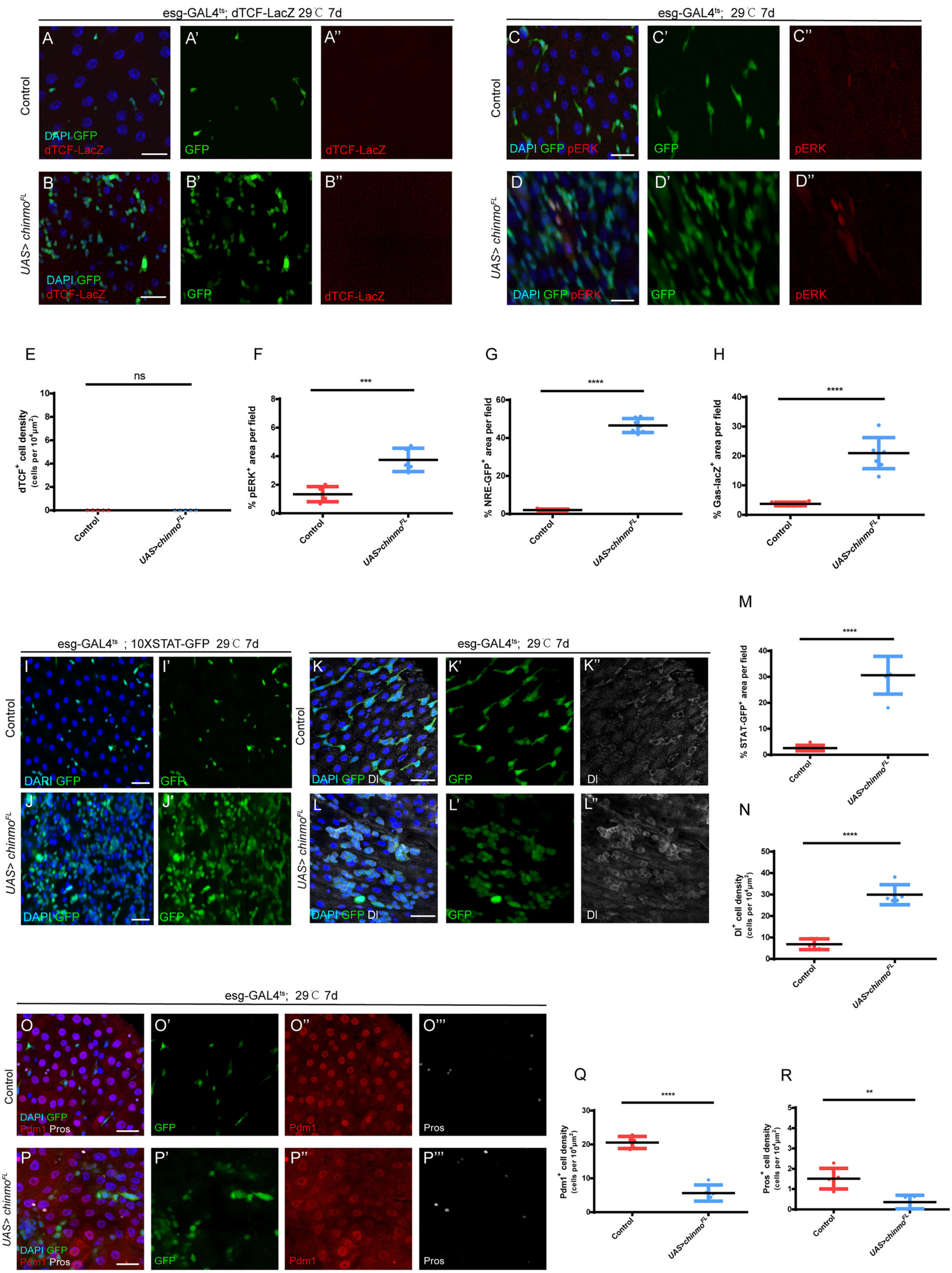
esg-GAL4ts; dTCF-LacZ 29 ℃ 7d
esg-GAL4ts; 29 ℃ 7d
Control
UAS> chinmoFL
A A' A'' B B' B'' C C' C'' D D' D''
DAPI GFP dTCF-LacZ
GFP
dTCF-LacZ
DAPI GFP pERK
pERK
E F G H
ns
***
****
dTCF+ cell density (cells per 10⁴μm²)
% pERK+ area per field
% NRE-GFP+ area per field
% Gas-lacZ+ area per field
Control
UAS>chinmoFL
esg-GAL4ts ; 10XSTAT-GFP 29 ℃ 7d
esg-GAL4ts; 29 ℃ 7d
I I' J J' K K' K'' L L' L''
DAPI GFP
DAPI GFP Dl
Dl
M N
% STAT-GFP+ area per field
Dl+ cell density (cells per 10⁴μm²)
esg-GAL4ts; 29 ℃ 7d
O O' O'' O''' P P' P'' P'''
DAPI GFP Pdm1 Pros
Pdm1
Pros
Q R
**
Pdm1+ cell density (cells per 10⁴μm²)
Pros+ cell density (cells per 10⁴μm²)

 © The Author(s)

Figure EV4. Effects of *chinmo* overexpression on ISC proliferation, differentiation, and JAK/STAT or Wnt signaling pathway activities.

(**A**, **B**) Representative images staining with LacZ (red) and DAPI (blue) of wild-type *esg^{ts} > GFP; dTCF-LacZ* (**A**), and *esg^{ts} > GFP; dTCF-LacZ; UAS-chinmo^{FL}* (**B**) flies. (**C**, **D**) Representative images staining with pERK (red) and DAPI (blue) of wild-type *esg^{ts} > GFP* (**C**), and *esg^{ts} > GFP; UAS-chinmo^{FL}* (**D**) flies. (**E**) Quantification of the number of dTCF$^+$ cells per 10^4 µm² within the midgut R4a region for *esg^{ts}>GFP* and *esg^{ts}>chinmo^{FL}* flies. Data were obtained from three independent biological experiments ($N = 3$), with $n = 6$ guts analyzed per genotype. (**F**) Quantification of the percentage of pERK$^+$ area relative to total tissue area (% pERK$^+$) in a fixed field of view within the midgut R4a region for *esg^{ts}>GFP* and *esg^{ts}>chinmo^{FL}* flies. Data were obtained from three independent biological experiments ($N = 3$), with $n = 6$ guts analyzed per genotype. $P = 0.0006$. (**G**) Quantification of the percentage of NRE-GFP$^+$ area relative to total tissue area (% NRE-GFP$^+$) in a fixed field of view within the midgut R4a region for *esg^{ts}>GFP* and *esg^{ts}>chinmo^{FL}* flies. Data were obtained from three independent biological experiments ($N = 3$), with $n = 6$ guts analyzed per genotype. $p < 0.0001$. (**H**) Quantification of the percentage of Gas-LacZ$^+$ area relative to total tissue area (% Gas-LacZ$^+$) in a fixed field of view within the midgut R4a region for *esg^{ts}>GFP* and *esg^{ts}>chinmo^{FL}* flies. Data were obtained from three independent biological experiments ($N = 3$), with $n = 6$ guts analyzed per genotype. $p < 0.0001$. (**I**, **J**) Representative images staining with DAPI (blue) of wild-type *esg^{ts}-GAL4; 10XSTAT92E-GFP* (**E**) and *esg^{ts}-GAL4; 10XSTAT92E-GFP; UAS-chinmo^{FL}* (**F**) flies. (**K**, **L**) Representative images staining with DAPI (blue) and anti-Dl (white) of wild-type *esg^{ts} > GFP* (**G**), and *esg^{ts} > GFP; UAS-chinmo^{FL}* (**H**) flies. (**M**) Quantification of the percentage of STAT-GFP$^+$ area relative to total tissue area (% STAT-GFP$^+$) in a fixed field of view within the midgut R4a region for *esg^{ts}>GFP* and *esg^{ts}>chinmo^{FL}* flies. Data were obtained from three independent biological experiments ($N = 3$), with $n = 6$ guts analyzed per genotype. $p < 0.0001$. (**N**) Quantification of the number of Dl$^+$ cells per 10^4 µm² within the midgut R4a region for *esg^{ts}>GFP* and *esg^{ts}>chinmo^{FL}* flies. Data were obtained from three independent biological experiments ($N = 3$), with $n = 6$ guts analyzed per genotype. $p < 0.0001$. (**O**, **P**) Representative images staining with DAPI (blue), anti-Pdm1 (red) and anti-Pros (white) antibodies of wild-type *esg^{ts} > GFP* (**I**), and *esg^{ts} > GFP; UAS-chinmo^{FL}* (**J**) flies. Flies were dissected at 7 days after eclosion. Scale bars, 25 µm. (**Q**) Quantification of the number of Pdm1$^+$ cells per 10^4 µm² within the midgut R4a region for *esg^{ts}>GFP* and *esg^{ts}>chinmo^{FL}* flies. Data were obtained from three independent biological experiments ($N = 3$), with $n = 6$ guts analyzed per genotype. $p < 0.0001$. (**R**) Quantification of the number of Pros$^+$ cells per 10^4 µm² within the midgut R4a region for *esg^{ts}>GFP* and *esg^{ts}>chinmo^{FL}* flies. Data were obtained from three independent biological experiments ($N = 3$), with $n = 6$ guts analyzed per genotype. $p = 0.0028$. Significance was measured with unpaired two-tailed *t*-tests. Mean ± SEM. $^{ns}p > 0.05$; $^{**}p < 0.01$; $^{***}p < 0.001$; $^{****}p < 0.0001$. Scale bars, 25 µm.

© The Author(s)

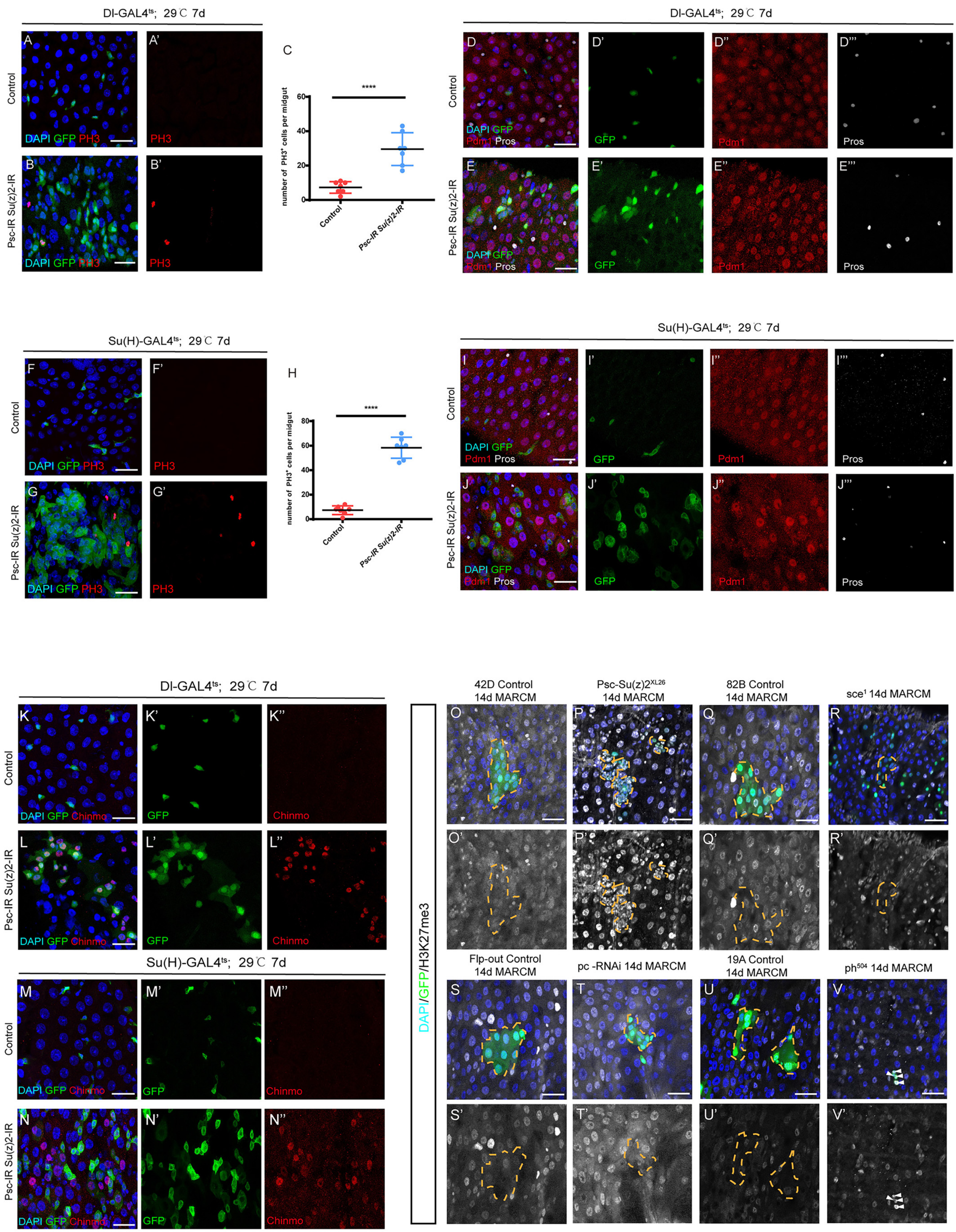

Dl-GAL4ts; 29℃ 7d
A
A'
B
B'
Control
Psc-IR Su(z)2-IR
DAPI GFP PH3
PH3
C
number of PH3+ cells per midgut
****
0
20
40
60
Control
Psc-IR Su(z)2-IR
Dl-GAL4ts; 29℃ 7d
D
D'
D''
D'''
E
E'
E''
E'''
DAPI GFP
Pdm1 Pros
GFP
Pdm1
Pros
Su(H)-GAL4ts; 29℃ 7d
F
F'
G
G'
H
0
20
40
60
80
Su(H)-GAL4ts; 29℃ 7d
I
I'
I''
I'''
J
J'
J''
J'''
Dl-GAL4ts; 29℃ 7d
K
K'
K''
L
L'
L''
DAPI GFP Chinmo
GFP
Chinmo
Su(H)-GAL4ts; 29℃ 7d
M
M'
M''
N
N'
N''
DAPI/GFP/H3K27me3
42D Control 14d MARCM
Psc-Su(z)2XL26 14d MARCM
82B Control 14d MARCM
sce1 14d MARCM
O
P
Q
R
O'
P'
Q'
R'
Flp-out Control 14d MARCM
pc -RNAi 14d MARCM
19A Control 14d MARCM
ph504 14d MARCM
S
T
U
V
S'
T'
U'
V'

 © The Author(s)

Figure EV5. Psc-Su(z)2 may function in EBs as well as ISCs to suppress tumorigenesis, without altering global H3K27me3 levels.

(**A**, **B**) Representative images staining with DAPI (blue) and anti-Phospho histone3 (PH3, red) of the following genotypes: Dl^{ts} > *GFP* control (**A**), and Dl^{ts} > *GFP; Psc-Su(z)2 RNAi* (**B**). Flies were dissected at 7 days after eclosion. (**C**) Quantification of the number of $PH3^{+}$ cells per midgut related to Fig. EV5A, B. Data were shown for both control and *Psc-Su(z)2 RNAi* flies. Data were obtained from three independent biological experiments ($N = 3$), with $n = 7$ guts analyzed per genotype. $p < 0.0001$. (**D**, **E**) Representative images staining with DAPI (blue), anti-Pdm1 (red) and anti-Pros (white) antibodies of Dl^{ts} > *GFP* control (**E**) and Dl^{ts} > *GFP; Psc-Su(z)2 RNAi* (**F**) flies. Flies were dissected at 7 days after eclosion. (**F**, **G**) Representative images staining with DAPI (blue) and anti-Phospho histone3 (PH3, red) of the following genotypes: $Su(H)^{ts}$ > *GFP* control (**C**), and $Su(H)^{ts}$ > *GFP; Psc-Su(z)2 RNAi* (**D**). Flies were dissected at 7 days after eclosion. (**H**) Quantification of the number of $PH3^{+}$ cells per midgut related to Fig. EV5F, G. Data were shown for both control and *Psc-Su(z)2 RNAi* flies. Data were obtained from three independent biological experiments ($N = 3$), with $n = 7$ guts analyzed per genotype. $p < 0.0001$. (**I**, **J**) Representative images staining with DAPI (blue), anti-Pdm1 (red) and anti-Pros (white) antibodies of $Su(H)^{ts}$ > *GFP* control (**G**) and $Su(H)^{ts}$ > *GFP; Psc-Su(z)2 RNAi* (**H**) flies. Flies were dissected at 7 days after eclosion. (**K**, **L**) Representative images staining with DAPI (blue) and anti-Chinmo (red) antibody of the following genotypes: Dl^{ts} > *GFP* control (**I**), and Dl^{ts} > *GFP; Psc-Su(z)2 RNAi* (**J**). Flies were dissected at 7 days after eclosion. (**M**, **N**) Representative images staining with DAPI (blue) and anti-Chinmo (red) antibody of the following genotypes: $Su(H)^{ts}$ > *GFP* control (**K**), and $Su(H)^{ts}$ > *GFP; Psc-Su(z)2 RNAi* (L). Flies were dissected at 7 days after eclosion. (**O–V**) MARCM clones marked by GFP co-stained with anti-H3K27me3 antibody (white) and DAPI (blue) of the following genotypes: wild-type *FRT 42D* (**A**); $Psc\text{-}Su(z)2^{XL26}$ (**B**), wild-type *FRT 82B* (C), sce^{1} (**D**), control Flp-out (**E**), pc^{RNAi} (Flp-out–driven RNAi) (**F**), wild-type *FRT 19 A* (**G**), and ph^{504}(**H**). Dashed lines depict the clone margin. The white arrowheads indicate the ph^{504} cells. Significance was measured with unpaired two-tailed *t*-tests. Mean ± SEM. $^{ns}p > 0.05$; $^{**}p < 0.01$; $^{***}p < 0.001$; $^{****}p < 0.0001$. Scale bars, 25 µm.

© The Author(s)
