# Supplementary material for: Psc and Su(z)2 safeguard intestinal stem cell identity and prevent chinmo-dependent tumorigenesis
Source: EMBO Rep. 2026 Jun 20;27(15):4433–61. doi: 10.1038/s44319-026-00837-x (PMC13458361; doi:10.1038/s44319-026-00837-x)
Supplement: Supplementary file 2 [file 44319_2026_837_MOESM1_ESM.pdf]

EMBO reports

Psc and Su(z)2 safeguard intestinal stem cell identity and prevent chinmo-dependent tumorigenesis

Ruxue Wei, Haimeng Yu, Qingyu Sun, Yongchao Zhang, Yaxin Yu, and Rongwen Xi

Corresponding author(s): Rongwen Xi (xirongwen@nibs.ac.cn)

Review Timeline:

Submission Date:	11th Jun 25
Editorial Decision:	31st Jul 25
Revision Received:	3rd Dec 25
Editorial Decision:	20th Jan 26
Revision Received:	20th Apr 26
Editorial Decision:	20th May 26
Revision Received:	26th May 26
Accepted:	3rd Jun 26

Editor: Achim Breiling

Transaction Report:

(Note: With the exception of the correction of typographical or spelling errors that could be a source of ambiguity, letters and reports are not edited. Depending on transfer agreements, referee reports obtained elsewhere may or may not be included in this compilation. Referee reports are anonymous unless the Referee chooses to sign their reports.)

1st Editorial Decision **31st Jul 2025**

Dear Dr. Xi

Thank you for the submission of your manuscript to EMBO reports. I have now received reports from the three referees that were asked to evaluate your study, which can be found at the end of this email. As you will see, the referees think that these findings are of high interest. However, they have several comments, concerns, and suggestions, indicating that a major revision of the manuscript is necessary to allow publication of the study in EMBO reports. As the reports are below, and all the referee concerns need to be addressed, I will not detail them here.

Given the constructive referee comments, I would thus like to invite you to revise your manuscript with the understanding that the concerns of the referees must be addressed in the revised manuscript and/or in a detailed point-by-point response, as indicated in your revision plan. Acceptance of your manuscript will depend on a positive outcome of a second round of review. It is EMBO reports policy to allow a single round of revision only and acceptance of the manuscript will therefore depend on the completeness of your responses included in the next, final version of the manuscript.

Revised manuscripts should be submitted within three months of a request for revision. Please contact me to discuss the revision (also by video chat) if you have questions or comments regarding the revision, or should you need additional time.

When submitting your revised manuscript, please also carefully review the instructions that follow below.

PLEASE NOTE THAT upon resubmission revised manuscripts are subjected to an initial quality control prior to exposition to re-review. Upon failure in the initial quality control, the manuscripts are sent back to the authors, which may lead to delays. Frequent reasons for such a failure are the lack of the data availability section (please see below) and the presence of statistics based on n=2 (the authors are then asked to present scatter plots or provide more data points).

When submitting your revised manuscript, we will require:

1) a .docx formatted version of the final manuscript text (including legends for main figures, EV figures and tables), but without the figures included. Figure legends should be compiled at the end of the manuscript text.

2) individual production quality figure files as .eps, .tif, .jpg (one file per figure), of main figures and EV figures. Please upload these as separate, individual files upon re-submission.

The Expanded View format, which will be displayed in the main HTML of the paper in a collapsible format, has replaced the Supplementary information. You can submit up to 5 images as Expanded View. Please follow the nomenclature Figure EV1, Figure EV2 etc. The figure legend for these should be included in the main manuscript document file in a section called Expanded View Figure Legends after the main Figure Legends section. Additional Supplementary material should be supplied as a single pdf file labeled Appendix. The Appendix should have page numbers and needs to include a table of content on the first page (with page numbers) and legends for all content. Please follow the nomenclature Appendix Figure Sx, Appendix Table Sx etc. throughout the text, and also label the figures and tables according to this nomenclature.

For more details, please refer to our guide to authors:
http://www.embopress.org/page/journal/14693178/authorguide#manuscriptpreparation

Please consult our guide for figure preparation:
http://wol-prod-cdn.literatumonline.com/pb-assets/embo-site/EMBOPress_Figure_Guidelines_061115-1561436025777.pdf

See also the guidelines for figure legend preparation:
https://www.embopress.org/page/journal/14693178/authorguide#figureformat

3) a .docx formatted letter INCLUDING the reviewers' reports and your detailed point-by-point responses to their comments. As part of the EMBO Press transparent editorial process, the point-by-point response is part of the Review Process File (RPF), which will be published alongside your paper.

4) a complete author checklist, which you can download from our author guidelines (https://www.embopress.org/page/journal/14693178/authorguide). Please insert page numbers in the checklist to indicate where the requested information can be found in the manuscript. The completed author checklist will also be part of the RPF.

Please also follow our guidelines for the use of living organisms, and the respective reporting guidelines:
http://www.embopress.org/page/journal/14693178/authorguide#livingorganisms

5) that primary datasets produced in this study (e.g. RNA-seq, ChIP-seq, structural and array data) are deposited in an appropriate public database. If no primary datasets have been deposited, please also state this in a dedicated section (e.g. 'No

primary datasets have been generated and deposited'), see below.

See also: http://embor.embopress.org/authorguide#datadeposition

Please remember to provide a reviewer password if the datasets are not yet public.

The accession numbers and database should be listed in a formal "Data Availability" section that follows the model below. This is now mandatory (like the COI statement). Please note that the Data Availability Section is restricted to new primary data that are part of this study. This section is mandatory. As indicated above, if no primary datasets have been deposited, please state this in this section

Data availability

The datasets produced in this study are available in the following databases:

- RNA-Seq data: Gene Expression Omnibus GSE46843 (https://www.ncbi.nlm.nih.gov/geo/query/acc.cgi?acc=GSE46843)
- [data type]: [name of the resource] [accession number/identifier/doi] ([URL or identifiers.org/DATABASE:ACCESSION])

*** Note - All links should resolve to a page where the data can be accessed. ***

Moreover, I have these editorial requests:

6) We now request the publication of original source data with the aim of making primary data more accessible and transparent to the reader. You will receive a separate email with instructions for providing source data with your revised manuscript, including information how to upload and organize the files.

7) Our journal encourages inclusion of *data citations in the reference list* to directly cite datasets that were re-used and obtained from public databases. Data citations in the article text are distinct from normal bibliographical citations and should directly link to the database records from which the data can be accessed. In the main text, data citations are formatted as follows: "Data ref: Smith et al, 2001" or "Data ref: NCBI Sequence Read Archive PRJNA342805, 2017". In the Reference list, data citations must be labeled with "[DATASET]". A data reference must provide the database name, accession number/identifiers and a resolvable link to the landing page from which the data can be accessed at the end of the reference. Further instructions are available at: http://www.embopress.org/page/journal/14693178/authorguide#referencesformat

8) Regarding data quantification and statistics, please make sure that the number "n" for how many independent experiments were performed, their nature (biological versus technical replicates), the bars and error bars (e.g. SEM, SD) and the test used to calculate p-values is indicated in the respective figure legends (also for EV and Appendix figures). Please also check that all the p-values are explained in the legend, and that these fit to those shown in the figure. Please provide statistical testing where applicable. Please avoid the phrase 'independent experiment', but clearly state if these were biological or technical replicates. Please also indicate (e.g. with n.s.) if testing was performed, but the differences are not significant. In case n=2, please show the data as separate datapoints without error bars and statistics. See also:
http://www.embopress.org/page/journal/14693178/authorguide#statisticalanalysis

If n<5, please show single datapoints for diagrams.

9) Please add scale bars of similar style and thickness to all microscopic images, using clearly visible black or white bars (depending on the background). Please place these in the lower right corner of the images themselves. Please do not write on or near the bars in the image but define the size in the respective figure legend.

10) Please also note our reference format:
http://www.embopress.org/page/journal/14693178/authorguide#referencesformat

11) We updated our journal's competing interests policy in January 2022 and request authors to consider both actual and perceived competing interests. Please review the policy https://www.embopress.org/competing-interests and update your competing interests if necessary. Please name this section 'Disclosure and Competing Interests Statement' and put it after the Acknowledgements section.

12) We now use CRediT to specify the contributions of each author in the journal submission system. CRediT replaces the author contribution section. Please use the free text box to provide more detailed descriptions and do NOT provide your final manuscript text file with an author contributions section. See also our guide to authors:
https://www.embopress.org/page/journal/14693178/authorguide#authorshipguidelines

13) All Materials and Methods need to be described in the main text using our 'Structured Methods' format, which is required for all research articles. According to this format, the Methods section should include a Reagents and Tools Table (listing key

reagents, experimental models, software, and relevant equipment and including their sources and relevant identifiers), uploaded as separate file, and a Methods section in which we encourage the authors to describe their methods using a step-by-step protocol format with bullet points, to facilitate the adoption of the methodologies across labs. More information on how to adhere to this format as well as downloadable templates (.doc) for the Reagents and Tools Table can be found in our author guidelines (section 'Structured Methods'):

https://www.embopress.org/page/journal/14693178/authorguide#manuscriptpreparation

14) Please add up to five keywords to the manuscript and order the sections like this, using only these names:
Title page - Abstract - Keywords - Introduction - Results - Discussion - Methods - Data availability section - Acknowledgements (please put here all the funding information) - Disclosure and Competing Interests Statement - References - Figure legends - Expanded View Figure legends

15) Please make sure that all the funding information is also entered into the online submission system and that it is complete and similar to the one in the acknowledgement section of the manuscript text file.

We would also welcome the submission of cover suggestions, or motifs to be used by our Graphics Illustrator in designing a cover.

As part of the EMBO publication's Transparent Editorial Process, EMBO reports publishes online a Review Process File (RPF) to accompany accepted manuscripts. This File will be published in conjunction with your paper and will include the referee reports, your point-by-point response and all pertinent correspondence relating to the manuscript.

You are able to opt out of this by letting the editorial office know (emboreports@embo.org). If you do opt out, the Review Process File link will point to the following statement: "No Review Process File is available with this article, as the authors have chosen not to make the review process public in this case."

I look forward to seeing a revised form of your manuscript when it is ready.

Yours sincerely,

Achim Breiling
Senior Editor
EMBO Reports

Referee #1:

Wei et al explore the phenotype of the inactivation of the genes Psc and Su(z)2 in the Drosophila intestine. They find that loss of both genes results in the formation of clusters of proliferative cells that lack the stem cell marker, Delta, and fail to produce cells with differentiated cell markers. Psc and Su(z)2 can participate in the PRC1 complex, but their loss of function phenotype is in apparent contrast to the inactivation of other components of this complex, including sce, pc, and ph. Consistent with Psc and Su(z)2 acting separately that PRC1 in the intestine, the Histone H2AK118Ub1 mark deposited by PRC1 in a sce-dependent manner, is not affected in Psc, Su(z)2 mutant clones. Transcriptomic analysis identified the chinmo as upregulated within Psc, Su(z)2 clones and is necessary and sufficient for some aspects of the phenotype, including excessive proliferation. Overall, this manuscript presents interesting data on the role of Psc and Su(z)2 in the intestine, however, many aspects need to be improved prior to publication including more robust quantification of many of the experiments. In addition, the comparison of RNAseq and ATACseq datasets in Figure 4 are not appropriate. The current manuscript seems hastily assembled.

Major points:
1. Many experiments lack appropriate controls and quantification essential to interpret the phenotype. I would imagine that these data have been acquired, but were not included in the manuscript, as to me, they are very essential for the interpretation of the data. These include:
Figure 1: Please quantify the size distribution of the clones in control and mutant contexts. It is not clear to me if all clones show this phenotype or only a fraction of them. For 1M-P, only P has the PH3 channel shown. This is very odd, please show it for all images. For 1I-L, wt and UAS-Psc, UAS-Su(z)2 alone clones are lacking. This is important to properly interpret the effect on the mutant clone. If there is a dominant negative effect on clone growth, for example, then the interpretation in the context of the double mutant changes.
Figure 2H and Figure 3: Control wildtypes clones with each of these markers (NRE-LacZ, GAS-LacZ, pERK, dTCF-LacZ) and quantification of clone sizes should be provided.
Figure 5C: All of the knockdowns were done in a Psc-Su(z)2 mutant context- this should be made clear on the graph.
Figure 6: Control wildtype clones for A-B' and some quantification of % chinmo positive clones or cells per clone are needed.

Figure 6: chinmo RNAi 1, and 2 KD alone controls are needed to interpret the reduction in E-H of clone size upon inactivation of chinmo. It is not clear to me from this figure whether chinmo is expressed at low levels and required for all ISC proliferation or whether it is very specific to the mutant context.
Figure 7: It is impossible to compare the various clones shown as they are all on different chromosomes and have different controls. Depending on the MARCM line used, there can be different proliferation rates of the clones, so it is important to present the controls for each MARCM line (19A or 42B, etc) as well, at least for 7A-D.
Figure 7: some quantification of the H2AK118Ub1 marks per cell should be shown.

**the images shown in Figure 6B,B', are duplicated in Figure 7A,A'. Minimally, this should be stated as such, but surely the authors have a second image at 14d. In addition, what is the scale? In Figure 6B,B', we are led to believe it is as 6A,A', however, this is not the same scale as seen in Figure 7. Please verify that there are no other cases of duplicated images in different figures.

2. In the "Statistics" section, it is mentioned that "Each experimental dataset included more than three biological replicates for analysis. All significance tests were analyzed by t tests, mean{plus minus}SEM. Significance P values: nsp > 0.05, ＊p < 0.05, ＊＊p < 0.01, ＊＊＊p < 0.001, ＊＊＊＊p < 0.0001." The authors need to give more detailed information per figure on what was done and what data is shown. For example, the n= for each quantification should be indicated on the graph. Many points are on top of each other and we cannot see simply from the graph. If we look at Figure 1F, the control has 10 data points and the mutant 11. Was this the pooled data of the 3 biological replicates? Is this just one replicate? If so, were are the other data?

3. As far as I could see, the tables with the RNAseq and ATACseq data are not available to reviewers.

4. What is being quantified exactly in Figure 1F? What does each point represent? A gut? I find it surprising that 20% of control clones have a Ph3+ cell.

5. It is confusing that 10XSTAT is being used to quantify STAT signaling upon overexpression of chinmo whereas GAS-LacZ was used in Figure 3. For consistency and comparison, it would be helpful to see the GAS-LacZ in the overexpression of chinmo.

6. The RNAseq comparision in Figure 4, will give many genes that are not of interest due the rather odd control used for comparison to the mutant condition. These samples differ in genotype, age and temperature. The control are FACS sorted esgGal4, tub-Gal80ts expression of GFP done for 7 days at 29{degree sign}c whereas the mutant are Marcm clones that have been induced by heatshock at 37{degree sign}C followed by 14d growth at 25{degree sign}C. A better experiment would have been to do both control and knockdown with esg, like in Figure 1P, which would have limited genetic and temperature variation. Also, it seems very odd to me that they show a venn diagrams with only the top 200 genes? ATACseq and RNAseq data from here are in my opinion, not very easy to interpret and I would favor removing this figure from the manuscript. Figure 5 has data that is somewhat easier to compare between samples.

7. There are inconsistencies throughout the manuscript on outlining clones or not- please be consistent.

8. The authors are likely aware that a relevant manuscript with similar finding has been posted on bioRxiv (https://doi.org/10.1101/2025.03.18.643963). This manuscript should be cited in this study so that readers can consider both works together. It does not distract from this study, but rather provides some supporting evidence for many of the conclusions.

9. In the manuscript on biorxiv, https://doi.org/10.1101/2025.03.18.643963, the authors suggest that the knockdown of Psc/Su(z)2 by esg causes some cells to lose esg and GFP expression. Do these authors find that? How might this change your interpretation of some of the data?

10. More details on how the clones and phenotypes were quantified is necessary, particularly with respect to "regionalization" of the phenotype that was mentioned.

11. If the authors could comment as to whether there are there any changes to K27me3, it would be helpful for the reader.

Referee #2:

Wei et al. describe a role of Polycomb group proteins, PSc and Su(z)2, in the maintenance of ISC fate and quiescence. The authors report that the loss of these two proteins causes 1) overproliferation and 2) loss of ISC identity without 3) differentiation, and that these phenotypes are contributed, at least in part, by upregulated Chinmo. Interestingly, these ISC defects are independent of commonly characterized regulatory pathways, including JAK/STAT, Wnt, and Notch. While the potential role of PSC and Su(z)2 in maintaining ISC identity is of significant importance and quite novel, the impact of this study is reduced by incomplete supporting evidence (Point 1 and 2) and limited mechanistic insight (Point 3 and 4). The details about the significant concerns are listed below:

Major:

1) Virtually no quantification and therefore sample sizes and statistics were provided for the vast majority of experiments. Experiments include a) clone size (or #GFP+ cells/area for RNAi) and cell size, b) readouts for MAPK, JAK/STAT, JNK, and Notch signaling, c) FACs experiments, d) #PH3+ cells/area after RNAi, e) apoptosis readout, and f) Chinmo and H2AK immunostaining.

2a) A major claim by the authors is that loss of function mutations for PSc and Su(z)2 cause loss of ISC identity, but there are issues concerning their support of this claim. The authors claim that GFP intensity is decreased after RNAi and while their FACs analyses reveal that some GFP+ cells from PSC/Su(z)2-depleted ISCs are indeed dimmer, there are many cells that seem just as bright as control ISCs. Quantification/statistical analyses of average intensity should be performed to more accurately demonstrate a potential decrease in esg activity. A significant difference would support their finding, and they should move this to the main text.

2b) The authors also compare Delta immunostaining, but their representative images do not demonstrate convincing staining especially when compared to what is commonly published in the field (including previous work from the lab - Li et al. 2020 Cell Rep). Delta should decorate the ISC cortex, which is not evident in E', and it is difficult to differentiate background versus cellular staining. Furthermore, there are very few Delta+ cells outside the clone in F', especially in comparison to the areas outside the clones in E' (which should be similar in staining profile). The potential role of PSC/Su(z)2 mutants in ISC identity loss in proliferating cells is an important finding, but the discrepancies in the images limit interpretation. Giving the crux of this finding to the study, the authors should provide additional examples of their images, include quantifications of Dl+ cells outside of clones per condition, and/or utilize additional strategies such as Delta-LacZ.

3a) It is not clear to me how relevant Chinmo is in the phenotypes caused by PSc/Su(z)2 loss of function (overproliferation, loss of ISC identity, and failure to differentiate). The authors identified Chinmo as a potential mechanism, but Chinmo manipulation seemed to only affect the overproliferation phenotype. In fact, the authors demonstrate that Chinmo overexpression still activated Notch and JAK/STAT, which are not activated after PSc/Su(z)2 disruption. Does Chinmo also activate ERK or Wnt? If so, these discrepancies imply that the mechanism in which PSc/Su(z)2 promotes division is different from Chinmo. ERK, JAK/STAT, and Wnt are not activated in PSc/Su(z)2 mutants, so how does PSc/Su(z)2 regulate mitosis independently of these pathways then? Finally, the authors should also strengthen their argument that PSc/Su(z)2 regulates proliferation independent of the above pathways by disrupting the pathways (i.e. with RNAi) in PSc/Su(z)2 mutant clones.

3b) Does manipulating Chinmo affect cell fate (i.e. affect Delta, Pdm1, Prospero numbers similar to PSc/Su(z)2 mutants)? If not through Chinmo, how does PSc/Su(z)2 loss of function cause loss of ISC identity and the inability to differentiate despite promoting division? Can the authors phenocopy or rescue these phenotypes by modulating other genes identified from their RNAseq data? Addressing these questions will be crucial for a proper understanding on how Psc/Su(z)2 maintains tissue homeostasis, as Chinmo seems to be only a part of the picture.

4) The potential function of PSc/Su(z)2 independent of PRC1 is interesting, but could be expanded a bit more. More insight should be provided on how PSc/Su(z)2 affects ISC processes independent of its 'canonical' role as part of PRC1. Alternatively, the authors could provide additional insight on how the other PRC1 components affects ISC processes differently than PSc/Su(z)2.

Minor:

5) Control RNAi should be used as a genetic control for RNAi experiments (such as RNAi against mCherry or Luciferase)

6) Controls need to be provided for Figure 7 (for clone size comparisons).

7) What is the timing regarding induction of MARCM clones and Gal4? Were flies heatshocked at 37º, and then immediately moved to 29º? Please clarify in the Methods.

8) It isn't clear exactly how FACs analysis was used to determine G1 vs S vs G2 cells. More details should be provided in the Methods.

9) The number of guts quantified should be noted in Figure Legends when using clones for sample sizes (i.e. n = X clones from Y flies).

10) ACI was defined in Figure 1, but should be defined in the first applicable subfigure of each other relevant Figure.

Referee #3:

Wei et al. describe how the loss of both Polycomb group genes, Psc and Su(z)2, in adult Drosophila midgut progenitors results in tumors consisting of cells that fail to differentiate and continue to proliferate. Indeed, Psc Su(z)2 mutant cells seem to lose their ISC identity and acquire an identity closer to developing neural tissue. Further, the authors find that tumor growth is not due to increased ERK activation or Wg/Wnt signaling. Rather, the authors report that Psc Su(z)2 tumor proliferation is due to Chinmo expression. They also show that chinmo does not block differentiation by inhibiting Notch and Jak-STAT signaling. Lastly, the authors show that loss of other PRC1 genes do not form tumors. Unfortunately, the data do not describe how Psc Su(z)2 mutant cells remain undifferentiated.

These findings are interesting, and likely important for human cancer. However, additional work needs to be performed to support some of the conclusions. See my concerns outlined below. Additionally, my concerns are further supported by the findings in a similar preprinted study Joly et al. (doi: https://doi.org/10.1101/2025.03.18.643963) about Psc Su(z)2 tumors. This study finds Psc Su(z)2 tumors to be not highly proliferative and require Stat92E, and not chinmo, for their proliferation. Moreover, the authors must determine which cell type gives rise to these tumors, ISCs or progenitors (EBs or EEps).

MAJOR concerns:

1. The title does not reflect the data provided. There is no data provided that suggests Chinmo expression alone can promote tumorigenesis by increasing both proliferation and blocking differentiation. Chinmo overexpression may cause hyperplasia though. Furthermore, it is not clear whether Psc Su(z)2 loss is important in ISCs or in a progenitor (EB, EEp) for tumorigenesis.
2. The authors never report whether the loss of Psc Su(z)2 affects the ISC or a progenitor (EB or EEp). MARCM analysis allows the labeling of one of two lineages- the ISC lineage or the progenitor. Are only ISC clones presented here? What happens to the progenitor (EB, EEp) clone? Images showing both types of clones and their differentiation should be provided. This will reveal whether the Psc Su(z)2 loss is required in ISCs or in a progenitor (EB, EEp) for tumorigenesis. This can be further supported by knockdown of Psc Su(z)2 with RNAi using ISC- or progenitor-specific GAL4 drivers.
3. The text states "prominent tumorous outgrowth in the anterior and middle midgut regions" due to loss of PSc Su(z)2. However, in Supplementary Fig. S1B, the overgrowth due to PSc Su(z)2 loss is more prominent in the posterior midgut. How do the authors explain this? The authors should quantify the number of Psc Su(z)2 clones by region (R1-R5) or in the anterior vs posterior midgut.
4. Do all of the Psc Su(z)2 mutant cells have the capacity to divide? This is not clear from the data. See Perdigoto et al., 2011.
5. To show that Psc Su(z)2 loss results in increased mitotic (PH3+) cells in the midgut, the authors analyzed the % of clones with PH3+ cells. The authors should plot the number of PH3+ cells per clone. This will help demonstrate whether most clones have only one PH3+ cell or do most clones contain multiple PH3+ cells. In Figure 1Q, please report the total PH3+ cell number per midgut rather than per view. This will help the reader compare the increased proliferation due to Psc Su(z)2 loss with other published work to assess its magnitude. As the authors have stated that the effects of Psc Su(z)2 loss are regional, the PH3+ cells should be quantified and reported in the anterior and posterior separately. This will clarify in which region increased proliferation is observed.
6. On page 5, the authors state "transgenic expression of either Psc or Su(z)2 was sufficient to rescue the phenotype caused by Su(z)2XL26". It is not clear how this was determined. Do these cells differentiate properly? The authors should examine whether these rescued clones grow to a similar size (cell number) as control clones and show that they can properly differentiate (Pdm1+, Pros+).
7. Does Psc Su(z)2 RNAi expression cause a decrease in Pdm1+ or Pros+ cells? The rapid accumulation of GFP is not sufficient to examine the failure of differentiation as the GFP can be inherited by progeny due to rapid proliferation. See Figure 1P.
8. It is not clear why the authors used GAS-lacZ to examine STAT activity in the midgut epithelium but later use the 10X STAT-DGFP reporter. The latter is more often used in the field. Do the levels of GAS-lacZ represent STAT activity in the adult midgut? The levels of ß-gal (GAS-lacZ) are very high in unchallenged midguts (Figure 3A) where Upd1-3 levels are normally expressed at low levels (Jiang et al. 2009; Buchon et al., 2009). Are these levels increased in the surrounding wild-type tissue due to the tumors? Since Upd1-3 levels increase with midgut damage, does GAS-LacZ increase with midgut damage? What does GAS-lacZ look like in tissue without tumors? Quantitation of the ß-gal (GAS-lacZ) signal is necessary to compare these levels. The authors should also repeat this experiment using 10X STAT-DGFP. A similar preprinted study about PSc Su(z)2 loss in progenitors found an increase in 10X STAT-DGFP within tumors. Moreover, this study reports that Stat92E is required for tumor growth.
9. The data in Figure 2F suggesting that ISCs or ISC identity are/is lost is not convincing as the Delta staining is not evident in the surrounding wild-type tissue. Further, the authors could use more than one ISC or progenitor marker, e.g., esg or mira. There are reagents available to look at a variety of ISC and progenitor markers, many of which are shown to decrease in Figure 4.
10. It's not clear why NRE-GFP was used to examine Notch signaling when the field mostly uses Su(H)-lacZ (Zeng et al., 2010).
11. In Figure 6I-J, the authors claim "significant increase in both phospho-Histone 3 mitotic cells and GFP+ progenitor cells", but only one PH3+ cell is observed in Figure 6J. The total number of PH3+ cells in chinmo expressing midguts should be determined as well as the number of PH3+ cells in the anterior vs posterior midgut.
12. Figure 6E-H: Do Psc Su(z)2 mutant clones expressing chinmo RNAi differentiate? Please determine if these clones contain Pdm1+ or Pros+ cells.
13. The knockdown efficiency for the chinmo RNAis must be determined by qRT-PCR and provided.

14. The preprint Joly et al (doi: https://doi.org/10.1101/2025.03.18.643963) should be cited.

MINOR concerns:

1. Abstract: the authors state that JAK-STAT signaling promotes ISC proliferation during homeostasis. However, Stat92E mutant clones grow to a similar cell number as control clones. See Jiang et al., 2009 and Beebe et al., 2010.
2. Abstract: the authors assume that all tumor cells are proliferative and diploid. There is no data provided that suggests this. See Perdigoto et al., 2011.
3. On Page 5, the authors state "loss of Notch signaling typically results in ISC proliferation and excess EE differentiation, forming clusters of both ISCs and EEs". The loss of Notch forms clusters of ISC- or progenitor-like cells that resemble EE precursors and Pros+ EEs (see Bardin et al, 2010; Patel et al., 2015).
4. In Figure 4C, SC should be sc for scute
5. On Page 8, "robustly activated" should say robustly expressed
6. While the reduction in Pdm1+ ECs is evident in Psc Su(z)2 double mutant clones, it is not clear why the control clones are not composed of nearly 70-90% Pdm1+ ECs? Does Pdm1 not label all ECs?
7. RNAis used in Figure 5C are not reported in the Materials and Methods.

We thank the reviewers for their careful reading of our manuscript and their constructive comments. We have now thoroughly addressed all the points raised. As a result, the revised manuscript is strengthened in both methodology and conclusions. Please find our point-by-point responses below.

Point-by-point responses

Reviewers' comments:

Reviewer #1:
Major points:
1. Many experiments lack appropriate controls and quantification essential to interpret the phenotype. I would imagine that these data have been acquired, but were not included in the manuscript, as to me, they are very essential for the interpretation of the data. These include:
Figure 1: Please quantify the size distribution of the clones in control and mutant contexts. It is not clear to me if all clones show this phenotype or only a fraction of them. For 1M-P, only P has the PH3 channel shown. This is very odd, please show it for all images. For 1I-L, wt and UAS-Psc, UAS-Su(z)2 alone clones are lacking. This is important to properly interpret the effect on the mutant clone. If there is a dominant negative effect on clone growth, for example, then the interpretation in the context of the double mutant changes.

We appreciate the reviewer's suggestions. We have now added the appropriate control experiments and statistical plots to the revised manuscript.

We quantified clone sizes in both control and mutant contexts. The mutant clones were marginally smaller because they produce small-cell cluster tumors (Fig EV1A). Furthermore, we have now included control clones with UAS-Psc or UAS-Su(z)2 alone, and these clones grow to comparable sizes with control wild-type clones (Fig 1M-1N).

Figure 2H and Figure 3: Control wildtypes clones with each of these markers (NRE-LacZ, GAS-LacZ, pERK, dTCF-LacZ) and quantification of clone sizes should be provided.

As requested, we have now included control clones carrying the same markers (NRE-LacZ, GAS-LacZ, pERK, dTCF-LacZ) and determined the percentage of marker-positive clones per gut (Fig 2H, 2J, and Fig 3).

Figure 5C: All of the knockdowns were done in a Psc-Su(z)2 mutant context- this should be made clear on the graph.

We have updated revised figures to convey the message with maximal clarity.

Figure 6: Control wildtype clones for A-B' and some quantification of % chinmo positive clones or cells per clone are needed.

We consistently observed Chinmo expression only in Psc/Su(z)2 mutant clones, but wild-type control clones showed no detectable Chinmo signal. And as requested, representative images of control wild-type clones and the corresponding quantitative analyses have been added to the revised Fig 6A and 6C.

Figure 6: chinmo RNAi 1, and 2 KD alone controls are needed to interpret the reduction in E-H of clone size upon inactivation of chinmo. It is not clear to me from this figure whether chinmo is expressed at low levels and required for all ISC proliferation or whether it is very specific to the mutant context.

To exclude the possibility that Chinmo knock-down per se affects ISC proliferation, here we include control clones with chinmo RNAi. These clones exhibit normal size and morphology, confirming that Chinmo depletion exerts a phenotype exclusively in the mutant background.

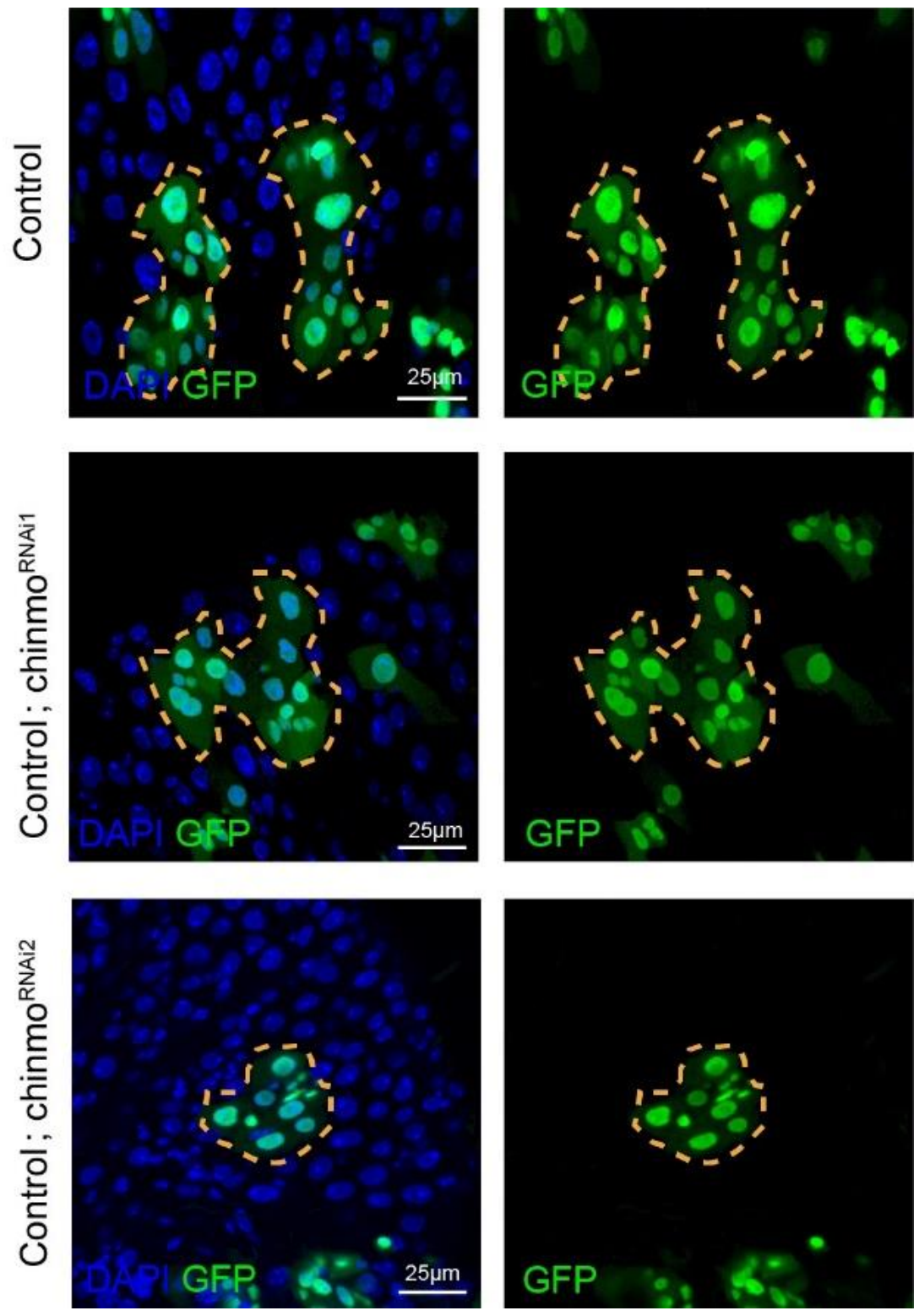

Figure 7: It is impossible to compare the various clones shown as they are all on different chromosomes and have different controls. Depending on the MARCM line used, there can be different proliferation rates of the clones, so it is important to present the controls for each MARCM line (19A or 42B, etc) as well, at least for

7A-D.

Thank you for this important request for controls. We have now generated matched controls for every MARCM line used in Fig 7 (see the updated figure).

Figure 7: some quantification of the H2AK118Ub1 marks per cell should be shown.

We have now quantified the proportion of H2AK118Ub1-positive cells per clone; the new data are presented in revised Fig 7Q.

**the images shown in Figure 6B,B', are duplicated in Figure 7A,A'. Minimally, this should be stated as such, but surely the authors have a second image at 14d. In addition, what is the scale? In Figure 6B,B', we are led to believe it is as 6A,A', however, this is not the same scale as seen in Figure 7. Please verify that there are no other cases of duplicated images in different figures.

Thank you for detecting the inadvertent duplication of the 14-day panel between Fig 6B and 7A. We have now replaced the repeated image in Fig 7 with an independent 14-day sample from the same genotype.

2. In the "Statistics" section, it is mentioned that "Each experimental dataset included more than three biological replicates for analysis. All significance tests were analyzed by t tests, mean{plus minus}SEM. Significance P values: nsp > 0.05, ∗p < 0.05, ∗∗p < 0.01, ∗∗∗p < 0.001, ∗∗∗∗p < 0.0001." The authors need to give more detailed information per figure on what was done and what data is shown. For example, the n= for each quantification should be indicated on the graph. Many points are on top of each other and we cannot see simply from the graph. If we look at Figure 1F, the control has 10 data points and the mutant 11. Was this the pooled data of the 3 biological replicates? Is this just one replicate? If so, were are the other data?

In the revised manuscript, the statistical plots now explicitly indicate the number of biological replicates, and all additional experimental details have been incorporated into the corresponding figure legends.

3. As far as I could see, the tables with the RNAseq and ATACseq data are not available to reviewers.

We sincerely apologize for this oversight. The RNA-seq and ATAC-seq data tables have now been re-uploaded.

4. What is being quantified exactly in Figure 1F? What does each point represent? A gut? I find it surprising that 20% of control clones have a Ph3+ cell.

In Fig 1F, each dot represents one gut, and the plotted value is the proportion of

PH3+ clones among all marked clones in that gut. In this experiment, the unexpectedly high baseline is most likely attributable to the stress condition of the flies. We have repeated the entire experiment under strictly controlled conditions and replaced the original quantification with the new, validated results (Fig 1F).

5. It is confusing that 10XSTAT is being used to quantify STAT signaling upon overexpression of chinmo whereas GAS-LacZ was used in Figure 3. For consistency and comparison, it would be helpful to see the GAS-LacZ in the overexpression of chinmo.

To maintain internal consistency, we have now included GAS-LacZ staining images upon Chinmo overexpression (Fig 6O-6P), and the results are consistent with the 10×STAT-GFP data previously shown.

6. The RNAseq comparision in Figure 4, will give many genes that are not of interest due the rather odd control used for comparison to the mutant condition. These samples differ in genotype, age and temperature. The control are FACS sorted esgGal4, tub-Gal80ts expression of GFP done for 7 days at 29{degree sign}c whereas the mutant are Marcm clones that have been induced by heatshock at 37{degree sign}C followed by 14d growth at 25{degree sign}C. A better experiment would have been to do both control and knockdown with esg, like in Figure 1P, which would have limited genetic and temperature variation. Also, it seems very odd to me that they show a venn diagrams with only the top 200 genes? ATACseq and RNAseq data from here are in my opinion, not very easy to interpret and I would favor removing this figure from the manuscript. Figure 5 has data that is somewhat easier to compare between samples.

We appreciate the reviewer's insightful comment. In the MARCM system, wild-type clones contain all types of cells including differentiated cells, therefore, we used the esg-Gal4, tub-Gal80ts, UAS-GFP as the baseline control. For the experimental group, Psc-Su(z)2 RNAi is effective but the phenotype is relatively mild compared to that caused by mutant clones. Consequently, Psc/Su(z)2 mutant clones provide a cleaner system for detecting transcriptional changes in ISCs.

In Fig 5A, we focused on the top 200 upregulated genes to capture those with the most robust expression changes. Meanwhile, there are 119 shared genes, providing a sufficient gene set for screening.

In Fig 4E, we intersected the ATAC-seq peaks with the RNA-seq transcriptome. By integrating chromatin accessibility and gene expression changes, we distilled a concise list of significantly up- and down-regulated genes. These data not only corroborate the loss of ISC identity, but also indicated a cell fate shift toward a

neural progenitor-like state.

7. There are inconsistencies throughout the manuscript on outlining clones or not-please be consistent.

We thank the reviewer for this suggestion and have outlined all clones in the revised figures.

8. The authors are likely aware that a relevant manuscript with similar finding has been posted on bioRxiv (https://doi.org/10.1101/2025.03.18.643963). This manuscript should be cited in this study so that readers can consider both works together. It does not distract from this study, but rather provides some supporting evidence for many of the conclusions.

We appreciate the reviewer's suggestion, and the manuscript has now been cited the revised version.

9. In the manuscript on biorxiv, https://doi.org/10.1101/2025.03.18.643963, the authors suggest that the knockdown of Psc/Su(z)2 by esg causes some cells to lose esg and GFP expression. Do these authors find that? How might this change your interpretation of some of the data?

We also observed that Psc/Su(z)2 knock-down leads to a marked reduction in GFP intensity (Fig EV2C). We interpret the decreased GFP intensity as indicative of loss of ISC identity, consistent with the down-regulation of Sox100B and Sox21a in Psc/Su(z)2 mutant clones (Fig EV2E-EV2F).

10. More details on how the clones and phenotypes were quantified is necessary, particularly with respect to "regionalization" of the phenotype that was mentioned.

As noted, the tumorous overgrowth is most pronounced in the anterior and middle midgut. To illustrate this spatial heterogeneity, we re-quantified (i) clone numbers, (ii) $PH3^+$ cell counts, and (iii) the percentage of $PH3^+$ clones per gut by region. These revised data are presented in the new Fig 1F, 1S and Fig EV1B.

11. If the authors could comment as to whether there are there any changes to K27me3, it would be helpful for the reader.

We have now examined H3K27me3 levels in control and mutant clones. The representative images are provided in new Fig EV5. No appreciable changes in global H3K27me3 were detected in any of the mutant conditions.

Reviewer #2:
Major:

1) Virtually no quantification and therefore sample sizes and statistics were provided for the vast majority of experiments. Experiments include a) clone size (or #GFP+ cells/area for RNAi) and cell size, b) readouts for MAPK, JAK/STAT, JNK, and Notch signaling, c) FACs experiments, d) #PH3+ cells/area after RNAi, e) apoptosis readout, and f) Chinmo and H2AK immunostaining.

We appreciate the reviewer's suggestions. In response, we have now added the following quantitative analyses to the revised manuscript:

a) Clone size: We measured the area of individual clones in both control and mutant guts. Consistent with the formation of small-cell tumors, mutant clones were slightly smaller than controls (Fig EV1A).
b) Readouts for signaling pathways: For MAPK, JAK/STAT, JNK and Notch signaling, we determined the percentage of marker-positive clones per gut, and the quantified data are consistent with our previous description (Fig 2J, 3C, 3F and 3I).
c) FACs experiments: The average GFP fluorescence intensity is now stated in the updated Fig EV2C.
d) $PH3^+$ cell after RNAi: We quantified $PH3^+$ cells per gut by regions (anterior, middle, and posterior midgut) after Psc-Su(z)2 RNAi. Knockdown of Psc and Su(z)2 significantly increased the number of $PH3^+$ cells in every region compared with control (Fig 1S).
e) Apoptosis readout: Quantitative analyses for TUNEL immunostaining are now provided in Fig EV3D.
f) Chinmo and H2AK immunostaining: We have quantified the percentage of Chinmo-positive clones per gut and the percentage of H2A-positive cells per clone, and those data are presented in revised Fig 6C and 7Q.

2a) A major claim by the authors is that loss of function mutations for PSc and Su(z)2 cause loss of ISC identity, but there are issues concerning their support of this claim. The authors claim that GFP intensity is decreased after RNAi and while their FACs analyses reveal that some GFP+ cells from PSC/Su(z)2-depleted ISCs are indeed dimmer, there are many cells that seem just as bright as control ISCs. Quantification/statistical analyses of average intensity should be performed to more accurately demonstrate a potential decrease in esg activity. A significant difference would support their finding, and they should move this to the main text.

To accurately illustrate the loss of ISC identity upon Psc-Su(z)2 RNAi, we analyzed the FACS data and have included the GFP mean fluorescence intensity (MFI) in the updated Fig EV2C. Additionally, we examined the expression of two other ISC markers, Sox100B and Sox21. The results showed that the expression of these ISC markers was significantly reduced in mutant clones (Fig EV2E-EV2F). These findings collectively support that mutations for Psc and

Su(z)2 cause loss of ISC identity.

2b) The authors also compare Delta immunostaining, but their representative images do not demonstrate convincing staining especially when compared to what is commonly published in the field (including previous work from the lab - Li et al. 2020 Cell Rep). Delta should decorate the ISC cortex, which is not evident in E', and it is difficult to differentiate background versus cellular staining. Furthermore, there are very few Delta+ cells outside the clone in F', especially in comparison to the areas outside the clones in E' (which should be similar in staining profile). The potential role of PSC/Su(z)2 mutants in ISC identity loss in proliferating cells is an important finding, but the discrepancies in the images limit interpretation. Giving the crux of this finding to the study, the authors should provide additional examples of their images, include quantifications of DI+ cells outside of clones per condition, and/or utilize additional strategies such as Delta-LacZ.

We appreciate the reviewer's helpful suggestions. Accordingly, we have replaced the original images with more representative examples (Fig 2E-2F) and quantified the number of DI^{+} cells outside clones per area. As shown here, no significant difference was detected between control and mutant conditions.

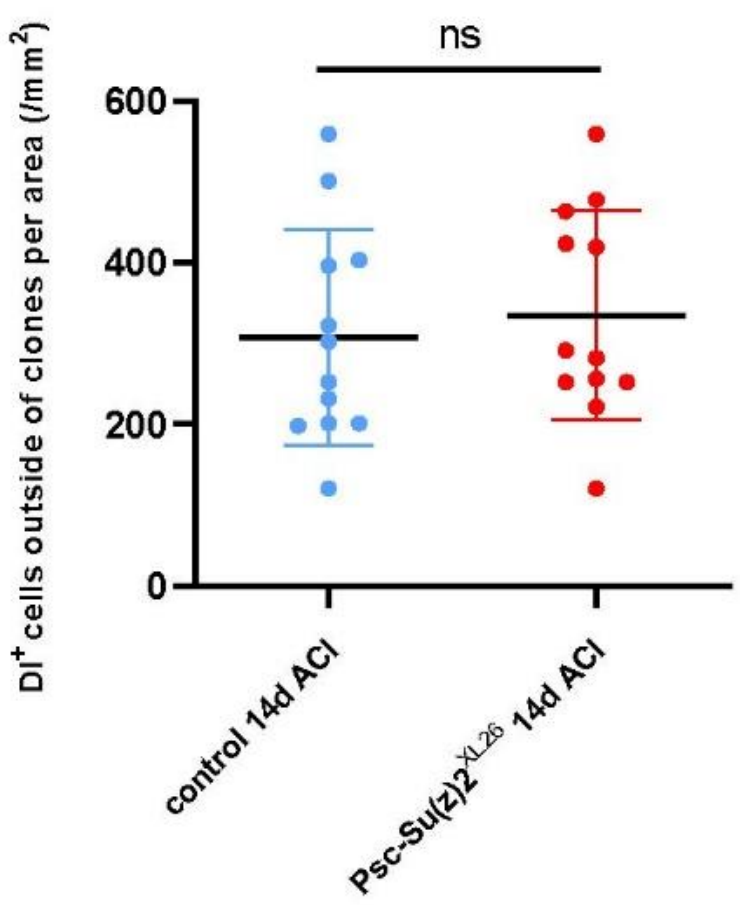

3a) It is not clear to me how relevant Chinmo is in the phenotypes caused by PSc/Su(z)2 loss of function (overproliferation, loss of ISC identity, and failure to differentiate). The authors identified Chinmo as a potential mechanism, but Chinmo manipulation seemed to only affect the overproliferation phenotype. In fact, the authors demonstrate that Chinmo overexpression still activated Notch and JAK/STAT, which are not activated after PSc/Su(z)2 disruption. Does Chinmo also activate ERK or Wnt? If so, these discrepancies imply that the mechanism in which PSc/Su(z)2 promotes division is different from Chinmo. ERK, JAK/STAT, and Wnt are not activated in PSc/Su(z)2 mutants, so how does PSc/Su(z)2 regulate mitosis independently of these pathways then? Finally, the authors should also strengthen their argument that PSc/Su(z)2 regulates proliferation independent of the above pathways by disrupting the pathways (i.e. with RNAi) in PSc/Su(z)2 mutant clones.

Thank you for raising this critical point. We found that Chinmo overexpression recapitulates only the over-proliferation phenotype of Psc/Su(z)2 mutants. To clarify this, we examined ERK and Wnt signaling in Chinmo-overexpressing clones (Fig EV4A-EV4D). The pERK and TCF-lacZ levels in these clones were similar to those in Psc/Su(z)2 mutants. However, unlike in Psc/Su(z)2 mutant clones, Notch or JAK/STAT activities were not suppressed in Chinmo-overexpressing cells (Fig 6M-6P). This suggests that Chinmo overexpression is not sufficient to alter MAPK signaling or to block Notch or JAK/STAT activities and thereby inhibit cell differentiation. Thus, Chinmo mediates the over proliferative phenotype in Psc/Su(z)2 mutants, but the differentiation block phenotype is mediated by other unknown mechanisms.

We additionally conducted experiments by knocking down the indicated pathway components within Psc/Su(z)2 mutant clones. Disruption of these pathways (EGFR, JAK/STAT and Wnt) did not alleviate the small-cell tumor phenotype, confirming that the phenotype of Psc/Su(z)2 mutants is independent of the tested signaling pathways.

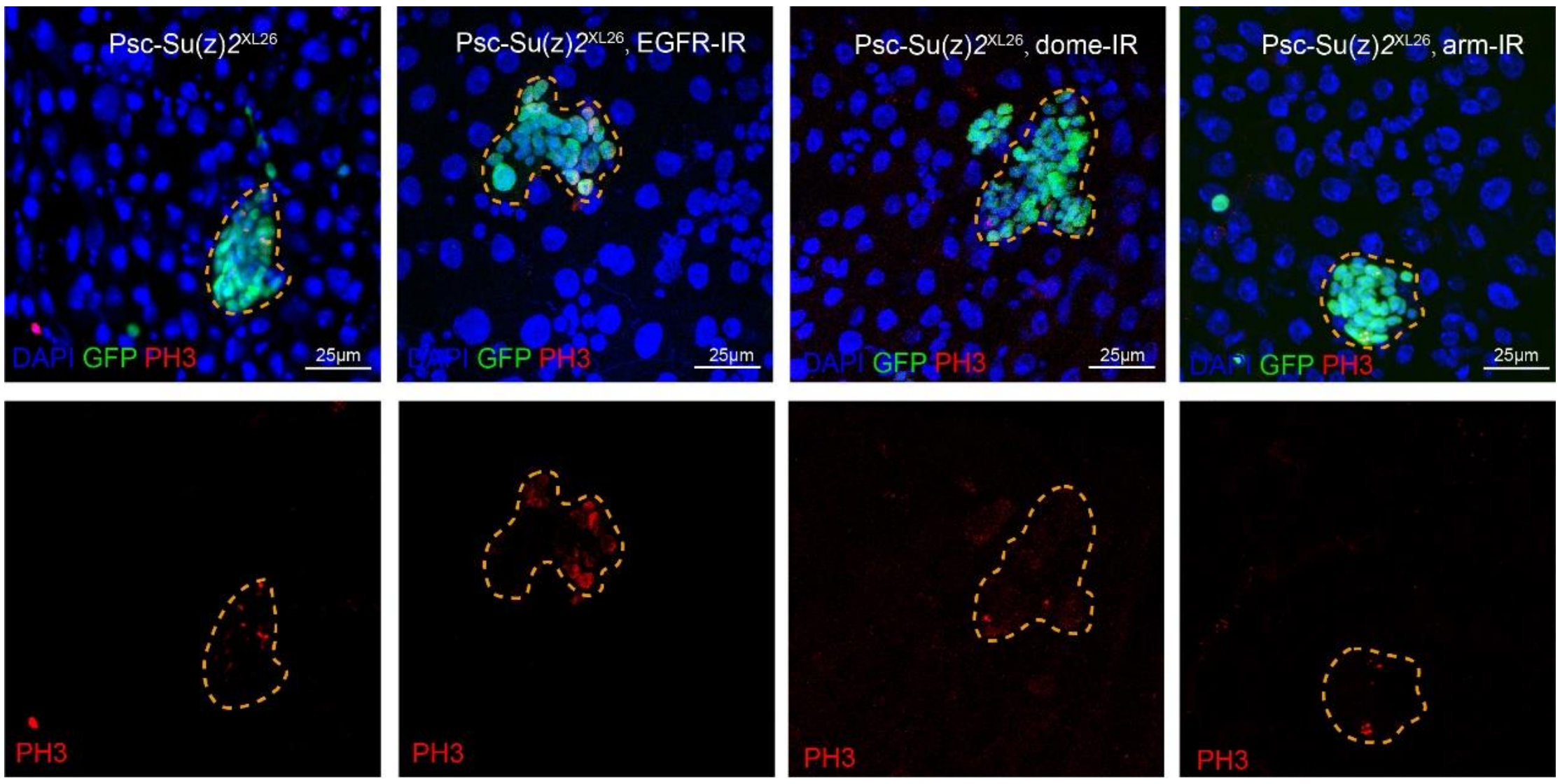

3b) Does manipulating Chinmo affect cell fate (i.e. affect Delta, Pdm1, Prospero numbers similar to PSc/Su(z)2 mutants)? If not through Chinmo, how does PSc/Su(z)2 loss of function cause loss of ISC identity and the inability to differentiate despite promoting division? Can the authors phenocopy or rescue these phenotypes by modulating other genes identified from their RNAseq data? Addressing these questions will be crucial for a proper understanding on how Psc/Su(z)2 maintains tissue homeostasis, as Chinmo seems to be only a part of the picture.

Chinmo overexpression induces ISC over-proliferation. This increases the number of undifferentiated progenitor cells, which in turn secondarily reduces the proportion of differentiated cells. However, as mentioned earlier, Chinmo

does not actively block differentiation. Therefore, we conclude that Chinmo mediates only the hyper-proliferation phenotype of Psc/Su(z)2 loss, while the differentiation arrest must be imposed by Chinmo-independent mechanisms. Our screen failed to identify any additional genes whose knockdown could rescue the Psc/Su(z)2 differentiation block; therefore, the Chinmo-independent mechanisms responsible for this differentiation phenotype remain to be determined.

4) The potential function of PSc/Su(z)2 independent of PRC1 is interesting, but could be expanded a bit more. More insight should be provided on how PSc/Su(z)2 affects ISC processes independent of its 'canonical' role as part of PRC1. Alternatively, the authors could provide additional insight on how the other PRC1 components affects ISC processes differently than PSc/Su(z)2.

Several speculative models could explain this specificity. One possibility is that during adult stages, the roles of other PRC1 components are diminished, positioning Psc/Su(z)2 as the central executor of PRC1-mediated transcriptional repression. A non-mutually exclusive model is that Psc/Su(z)2 represses a distinct set of targets through mechanisms independent of the canonical PRC1 complex.

Minor:

5) Control RNAi should be used as a genetic control for RNAi experiments (such as RNAi against mCherry or Luciferase)

To exclude off-target effects, we expressed luciferase-RNAi in Psc/Su(z)2 mutant clones. As shown here, these clones display the same phenotype as non-RNAi mutant controls, supporting the notion that the observed defects are specific to the target gene knock-down.

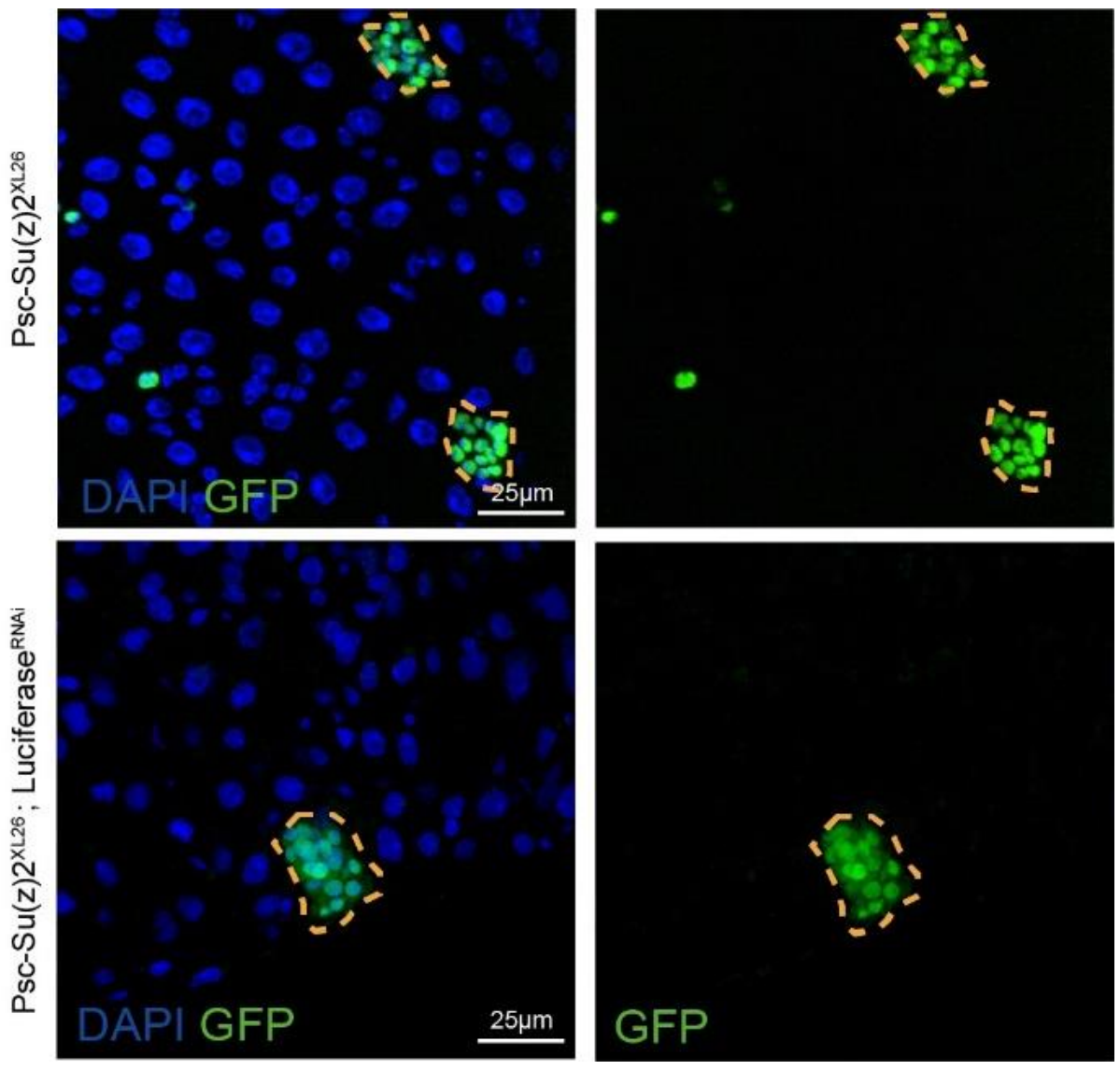

6) Controls need to be provided for Figure 7 (for clone size comparisons).

We have now added controls, and the revised data are presented in the updated Fig 7.

7) What is the timing regarding induction of MARCM clones and Gal4? Were flies heatshocked at 37º, and then immediately moved to 29º? Please clarify in the Methods.

For mosaic analysis, flies aged 4-6 days were collected and heat-shocked in 37℃ for 1 hour. Following heat shock, the flies were cultivated for another 1-2 weeks at 25℃. For GAL4 induction, flies aged 4-6 days were collected and shifted to 29℃ for 7 days. We have updated this information in the Materials and Methods section.

8) It isn't clear exactly how FACs analysis was used to determine G1 vs S vs G2 cells. More details should be provided in the Methods.

We have updated this information in the Methods of the revised manuscript.

9) The number of guts quantified should be noted in Figure Legends when using clones for sample sizes (i.e. n = X clones from Y flies).

The number of guts analyzed is now explicitly stated in every relevant graph (denoted as "n = X"), and the figure legends have been expanded to include a detailed description of the number of quantified clones.

10) ACI was defined in Figure 1, but should be defined in the first applicable subfigure of each other relevant Figure.

We have added the definition of ACI in each figure legend.

Reviewer #3:
MAJOR concerns:

1. The title does not reflect the data provided. There is no data provided that suggests Chinmo expression alone can promote tumorigenesis by increasing both proliferation and blocking differentiation. Chinmo overexpression may cause hyperplasia though. Furthermore, it is not clear whether Psc Su(z)2 loss is important in ISCs or in a progenitor (EB, EEp) for tumorigenesis.

Agreed. Because Chinmo is necessary but not sufficient to recapitulate the full Psc/Su(z)2 tumor phenotype, we have revised the manuscript title from "Polycomb group genes Psc and Su(z)2 safeguard intestinal stem cell identity

and prevent chinmo-mediated tumorigenesis" to "Polycomb group genes Psc and Su(z)2 safeguard intestinal stem cell identity and prevent chinmo-dependent tumorigenesis".

2. The authors never report whether the loss of Psc Su(z)2 affects the ISC or a progenitor (EB or EEp). MARCM analysis allows the labeling of one of two lineages- the ISC lineage or the progenitor. Are only ISC clones presented here? What happens to the progenitor (EB, EEp) clone? Images showing both types of clones and their differentiation should be provided. This will reveal whether the Psc Su(z)2 loss is required in ISCs or in a progenitor (EB, EEp) for tumorigenesis. This can be further supported by knockdown of Psc Su(z)2 with RNAi using ISC- or progenitor-specific GAL4 drivers.

We did observe occasional single-cell clones: the majority remained as undifferentiated cells, while single EC clone could also be detected. We therefore infer that Psc-Su(z)2 activity is required in EBs and EEPs for their differentiation, and the single EC clones may be due to the perdurance of Psc-Su(z)2 protein products.

3. The text states "prominent tumorous outgrowth in the anterior and middle midgut regions" due to loss of PSc Su(z)2. However, in Supplementary Fig. S1B, the overgrowth due to PSc Su(z)2 loss is more prominent in the posterior midgut. How do the authors explain this? The authors should quantify the number of Psc Su(z)2 clones by region (R1-R5) or in the anterior vs posterior midgut.

We thank the reviewer for raising this interesting point. We do not have the answer either, but we speculate that the over-proliferation triggered by Psc/Su(z)2 knock down (esgGAL4> Psc/Su(z)2-RNAi) in Fig EV2B (updated) is concentrated in the posterior midgut because the baseline activity of ISCs is relatively high in this region. By contrast, the tumorous outgrowth observed in Psc/Su(z)2 mutant clones preferentially arises in the anterior and middle midgut, and this regional shift is probably due to regional differences in cell competition between the tumor cells and the neighboring wild type ISCs.

4. Do all of the Psc Su(z)2 mutant cells have the capacity to divide? This is not clear from the data. See Perdigoto et al., 2011.

The Psc Su(z)2 mutant cells seem to be homozygous based on the similar gene marker expression. Furthermore, under homeostatic conditions, only 2–3 PH3-positive cells are typically seen along the entire midgut, although the tissue contains far more ISCs. And we frequently observe 2-3 $PH3^+$ cells in each Psc Su(z)2 mutant tumor clones. These results indicate that the mutant population as a whole is hyper-proliferative, leading us to infer that virtually all Psc Su(z)2 mutant cells have the ability to divide.

5. To show that Psc Su(z)2 loss results in increased mitotic (PH3+) cells in the midgut, the authors analyzed the % of clones with PH3+ cells. The authors should plot the number of PH3+ cells per clone. This will help demonstrate whether most clones have only one PH3+ cell or do most clones contain multiple PH3+ cells. In Figure 1Q, please report the total PH3+ cell number per midgut rather than per view. This will help the reader compare the increased proliferation due to Psc Su(z)2 loss with other published work to assess its magnitude. As the authors have stated that the effects of Psc Su(z)2 loss are regional, the PH3+ cells should be quantified and reported in the anterior and posterior separately. This will clarify in which region increased proliferation is observed.

We have quantified the number of PH3$^+$ cells per individual clone. Whereas virtually all control clones contain only 0 or 1 PH3$^+$ cell, a subset of mutant clones harbor 2–3 PH3$^+$ cells (Fig EV1C). In addition, we updated the PH3$^+$ cell per gut analysis and included regional annotation (anterior, middle, and posterior midgut) to illustrate the spatial distribution of mitotic activity (Fig 1S).

6. On page 5, the authors state "transgenic expression of either Psc or Su(z)2 was sufficient to rescue the phenotype caused by Su(z)2XL26". It is not clear how this was determined. Do these cells differentiate properly? The authors should examine whether these rescued clones grow to a similar size (cell number) as control clones and show that they can properly differentiate (Pdm1+, Pros+).

We have now quantified clone size and differentiation in the rescue experiment. As anticipated, transgenic re-introduction of Psc or Su(z)2 into Su(z)2^{XL26} mutant clones yields slightly smaller clones than wild-type controls, consistent with a mild growth delay. Importantly, the rescued clones contain normal numbers of Pdm1$^+$ enterocytes and Pros$^+$ enteroendocrine cells (Fig EV1D-EV1H), demonstrating that restoring Psc or Su(z)2 function is sufficient to allow proper differentiation while only minimally affecting overall clone expansion.

7. Does Psc Su(z)2 RNAi expression cause a decrease in Pdm1+ or Pros+ cells? The rapid accumulation of GFP is not sufficient to examine the failure of differentiation as the GFP can be inherited by progeny due to rapid proliferation. See Figure 1P.

Knockdown of Psc-Su(z)2 indeed leads to a marked reduction of both Pdm1$^+$ and Pros$^+$ differentiated cells. We have added immunostaining and quantification for Pdm1 and Pros under Psc-Su(z)2 RNAi conditions, and the results are presented in the updated Fig EV2G-EV2J.

8. It is not clear why the authors used GAS-lacZ to examine STAT activity in the midgut epithelium but later use the 10X STAT-DGFP reporter. The latter is more often used in the field. Do the levels of GAS-lacZ represent STAT activity in the adult

midgut? The levels of ß-gal (GAS-lacZ) are very high in unchallenged midguts (Figure 3A) where Upd1-3 levels are normally expressed at low levels (Jiang et al. 2009; Buchon et al., 2009). Are these levels increased in the surrounding wild-type tissue due to the tumors? Since Upd1-3 levels increase with midgut damage, does GAS-LacZ increase with midgut damage? What does GAS-lacZ look like in tissue without tumors? Quantitation of the ß-gal (GAS-lacZ) signal is necessary to compare these levels. The authors should also repeat this experiment using 10X STAT-DGFP. A similar preprinted study about PSc Su(z)2 loss in progenitors found an increase in 10X STAT-DGFP within tumors. Moreover, this study reports that Stat92E is required for tumor growth.

We appreciate the reviewer's helpful suggestions. We did not use the 10×STAT-GFP reporter within mutant clones because its GFP signal overlaps with the GFP-based clone marker. To maintain consistency, we instead added representative images of GAS-LacZ staining in Chinmo-overexpressing tissue (Fig 6O-6P). For direct comparison of the levels of GAS-LacZ, we also included a control panel confirming that GAS-LacZ levels in cells adjacent to mutant clones are indistinguishable from those in control midguts (Fig 3A).

9. The data in Figure 2F suggesting that ISCs or ISC identity are/is lost is not convincing as the Delta staining is not evident in the surrounding wild-type tissue. Further, the authors could use more than one ISC or progenitor marker, e.g., esg or mira. There are reagents available to look at a variety of ISC and progenitor markers, many of which are shown to decrease in Figure 4.

We thank the reviewer for this suggestion. We have replaced the previous figures with more representative Dl-staining images (revised Fig 2E-2F). In addition, we have examined the expression of two additional ISC markers, Sox100B and Sox21a, within mutant clones. Compared with ISCs outside the clones, Sox100B and Sox21a levels were significantly reduced in Psc/Su(z)2 mutant clones (Fig EV2E-EV2F).

10. It's not clear why NRE-GFP was used to examine Notch signaling when the field mostly uses Su(H)-lacZ (Zeng et al., 2010).

To clarify, "NRE" is the abbreviation for Notch Responsive Element, and "NRE-lacZ" specifically denoting the Gbe-Su(H)m8-lacZ (or named as Su(H)-lacZ) reporter used in this study.

11. In Figure 6I-J, the authors claim "significant increase in both phospho-Histone 3 mitotic cells and GFP+ progenitor cells", but only one PH3+ cell is observed in Figure 6J. The total number of PH3+ cells in chinmo expressing midguts should be determined as well as the number of PH3+ cells in the anterior vs posterior midgut.

We have now quantified PH3-positive cells along the *chinmo*-overexpressing midguts with regional information (anterior, middle, and posterior midgut), in revised Fig 6L.

12. Figure 6E-H: Do Psc Su(z)2 mutant clones expressing chinmo RNAi differentiate? Please determine if these clones contain Pdm1+ or Pros+ cells.

No, they do not. We have now provided Pdm1 and Pros immunostaining in Psc/Su(z)2 mutant clones expressing *chinmo* RNAi. The majority of these small clones contain no detectable Pdm1$^+$ or Pros$^+$ cells (Fig EV3A-EV3B), suggesting that chinmo depletion in the mutant background fails to rescue the differentiation block.

13. The knockdown efficiency for the chinmo RNAis must be determined by qRT-PCR and provided.

To determine the efficiency for the knock-down of chinmo, we performed anti--Chinmo staining in *chinmo* RNAi lines. As shown here, Chinmo signal became undetectable in Psc/Su(z)2 mutant clones with either chinmo-RNAi-1 or chinmo-RNAi-2 expression, suggesting that both RNAi lines are highly effective.

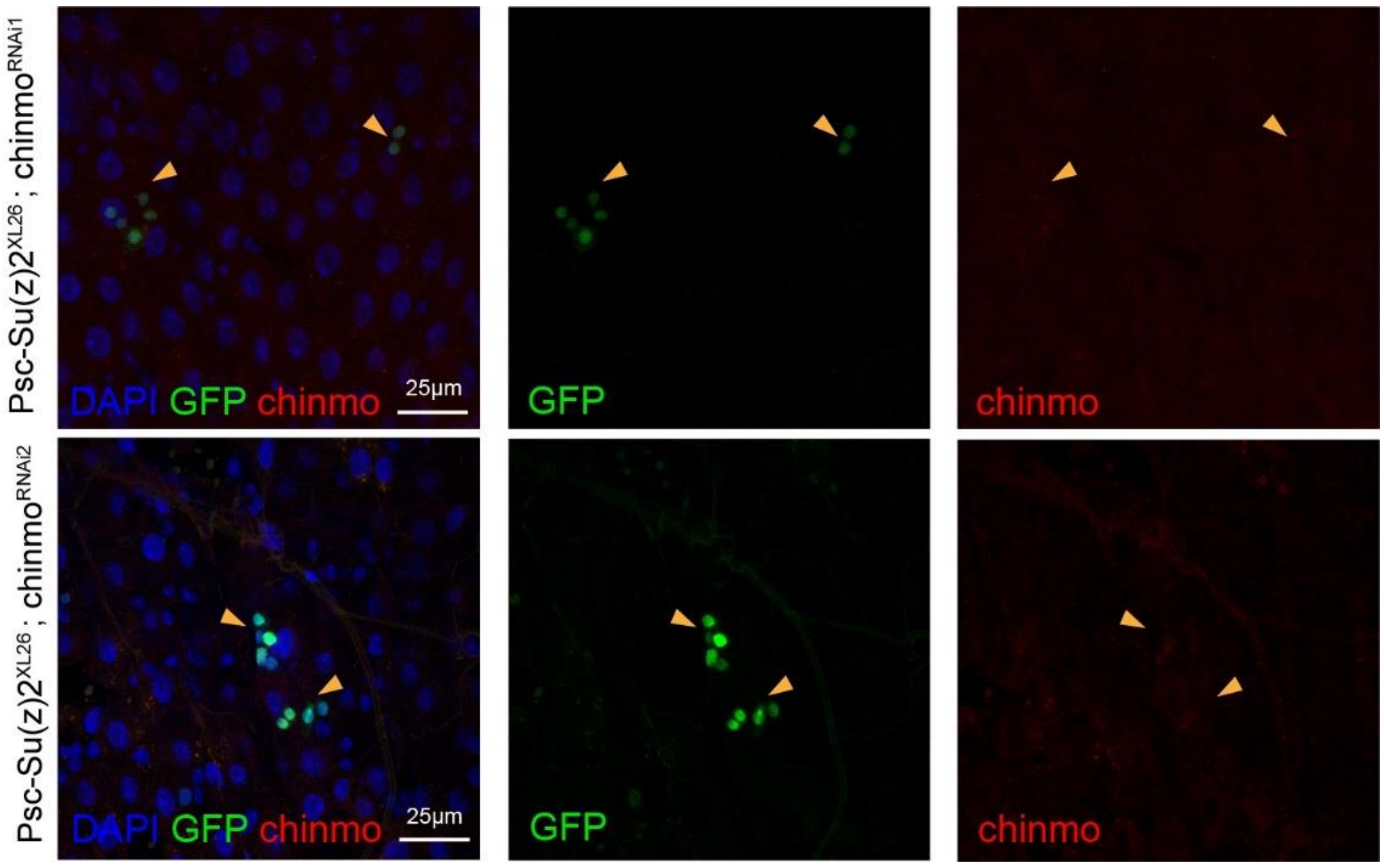

14. The preprint Joly et al (doi: https://doi.org/10.1101/2025.03.18.643963) should be cited.

We appreciate the reviewer's suggestion, and this preprint has been cited in the revised manuscript.

MINOR concerns:

1. Abstract: the authors state that JAK-STAT signaling promotes ISC proliferation during homeostasis. However, Stat92E mutant clones grow to a similar cell number as control clones. See Jiang et al., 2009 and Beebe et al., 2010.

There are differing views in the field regarding whether JAK/STAT signaling regulates ISC proliferation during normal homeostasis. To remain neutral on this unsettled question, we have revised the corresponding statement in the Abstract accordingly.

2. Abstract: the authors assume that all tumor cells are proliferative and diploid. There is no data provided that suggests this. See Perdigoto et al., 2011.

Good point. We have revised the description in the Abstract accordingly.

3. On Page 5, the authors state "loss of Notch signaling typically results in ISC proliferation and excess EE differentiation, forming clusters of both ISCs and EEs". The loss of Notch forms clusters of ISC- or progenitor-like cells that resemble EE precursors and Pros+ EEs (see Bardin et al, 2010; Patel et al., 2015).

Thank you for this point. We have revised the statement to clarify that the clusters consist of ISC-like progenitors and Pros^{+} EE precursors, rather than fully differentiated ISCs and EEs.

4. In Figure 4C, SC should be sc for scute

Thank you for noticing this typographical error. We have changed "SC" to the correct lowercase "sc" in Fig 4C and its legend.

5. On Page 8, "robustly activated" should say robustly expressed

Corrected.

6. While the reduction in Pdm1+ ECs is evident in Psc Su(z)2 double mutant clones, it is not clear why the control clones are not composed of nearly 70-90% Pdm1+ ECs? Does Pdm1 not label all ECs?

With the antibody batch used here, Pdm1 expression was most prominent in mature ECs. Its expression in early ECs or late EBs was relatively low and often ambiguous, potentially leading to an underestimation of the total EC number.

7. RNAis used in Figure 5C are not reported in the Materials and Methods.

Thank you for pointing out this omission. The complete genotypes of all RNAi lines used in Fig 5C (including stock numbers and sources) have now been added to the "*Drosophila* stocks" subsection of Materials and Methods.

1st Revision - Editorial Decision 20th Jan 2026

Dear Dr. Xi,

Thank you for the submission of your revised manuscript to our editorial offices. I have now received the reports from the three referees that I asked to re-evaluate the study, you will find below.

As you will see, all three referees have remaining concerns and suggestions to improve the manuscript. Upon cross-commenting, all three referees indicated that the findings are interesting and contain important data, and that a further revision should be invited. I thus ask you to address the remaining concerns and the editorial points below in a final revised manuscript. Please also provide a detailed final p-b-p-response regarding the remaining referee points and the editorial requests with your revised manuscript submission.

Please update the source data files if changes to the main figure panels are made during final revision!

Editorial requests:

- Please provide a final title with not more than 100 characters.

- Please remove the highlights from the title page. These can be used for the bullet points (see below).

- Please add up to 5 keywords to the manuscript and order the sections like this, using only these names:
Title page - Abstract - Keywords - Introduction - Results - Discussion - Methods - Data availability section - Acknowledgements (please include here all the funding information) - Disclosure and Competing Interests Statement - References - Figure legends - Expanded View Figure legends

- We now use CRediT to specify the contributions of each author in the journal submission system. CRediT replaces the author contribution section. Please use the free text box to provide more detailed descriptions and do NOT provide your final manuscript text file with an author contributions section. See also our guide to authors (section 'Author contributions'): https://link.springer.com/journal/44319/submission-guidelines#cms-Revised-submissions

- Please remove now the referee token from the Data Availability section and make sure that the datasets are public latest upon online publication of the manuscript.

- Please change the callouts to the movies to 'Movie EVx'.

- Please check again that the number "n" for how many independent experiments were performed, their nature (biological versus technical replicates), the bars and error bars (e.g. SEM, SD) and the test used to calculate p-values is indicated in the respective figure legends (main, EV and Appendix figures). Please also check that all the p-values are explained in the legend, and that these fit to those shown in the figure. Please provide statistical testing where applicable. Please avoid the phrase 'independent experiment' but clearly state if these were biological or technical replicates. Please also indicate (e.g. with n.s.) if testing was performed, but the differences are not significant. In case n=2, please show the data as separate datapoints without error bars and statistics. See also:

https://link.springer.com/journal/44319/submission-guidelines#cms-Figure-and-data-presentation

If n<5, please show single datapoints for diagrams. Presently, some diagrams seems to show incomplete statistics or miss the 'ns'. Please check. Moreover:
- Please note that the exact p values are not provided in the legends of figures 1F, S; 2C, D, G, J; 3C, F; 5C, 6I, L; EV1 C, EV2 I, J
- Please indicate the statistical test used for data analysis in the legends of figure 4B.

- There are callouts to supplementary figures (Figure Sx) in the manuscript text file (e.g. in the legends). Please update these (using Figure EVx).

- Please remove the Reagents and Tools Table from the manuscript text file and upload this as separate file. More information on how to adhere to this format as well as downloadable templates (.doc) for the Reagents and Tools Table can be found in our author guidelines (section 'Structured Methods'):

https://link.springer.com/journal/44319/submission-guidelines#cms-Manuscript-organisation-and-formatting

- Tables 1-3 are datasets and need to be named and uploaded as Dataset EV1-EV3. Please change the names in all places: source file names, titles in the system, and callouts in the manuscript. Please add a legend for each of these on the first TAB of the excel files.

In addition, I would need from you uploaded separately:
- a short, two-sentence summary of the manuscript (not more than 35 words).
- two to four short (!) bullet points highlighting the key findings of your study (two lines each).
- a schematic summary figure as separate file that provides a sketch of the major findings (not a data image) in jpeg or tiff format (with the exact width of 550 pixels and a height of not more than 400 pixels) that can be used as a visual synopsis on our website.

I look forward to seeing the further revised version of your manuscript when it is ready. Please let me know if you have questions regarding the revision.

Yours sincerely,

Achim Breiling
Senior Editor
EMBO Reports

Referee #1:

While the authors have addressed a number of important points (adding quantification), there are still points of mine to which they did not respond or said that something was modified, when it was not.

Major points that they did not fully address are regarding their statistics and quantification and their RNA/ATACseq data sets.

Statistics and Quantification:

In the first round, I noted that more detail was required to understand how many times an experiment was performed, how many intestines per replicate, the exact number of clones, etc.

The discussion is reproduced here:

Round 1
My original point 2 " In the "Statistics" section, it is mentioned that "Each experimental dataset included more than three biological replicates for analysis. All significance tests were analyzed by t tests, mean+/- SEM. Significance P values: nsp > 0.05, * p < 0.05,
* * p < 0.01, * * * p < 0.001, * * * * p < 0.0001." The authors need to give more detailed information per figure on what was done and what data is shown. For example, the n= for each quantification should be indicated on the graph. Many points are on top of each other and we cannot see simply from the graph. If we look at Figure 1F, the control has 10 data points and the mutant 11. Was this the pooled data of the 3 biological replicates? Is this just one replicate? If so, where are the other data?"

Authors "In the revised manuscript, the statistical plots now explicitly indicate the number of biological replicates, and all additional experimental details have been incorporated into the corresponding figure legends."

I am very confused. The statistics plots do NOT seem to indicate the number of biological replicates. For example, in 1F, they plot the number of guts, not the number of biological replicates.

Similarly, in point 4 from my original review:
4. What is being quantified exactly in Figure 1F? What does each point represent? A gut? I find it surprising that 20% of control clones have a Ph3+ cell.

In Fig 1F, each dot represents one gut, and the plotted value is the proportion of $PH3^+$ clones among all marked clones in that gut. In this experiment, the unexpectedly high baseline is most likely attributable to the stress condition of the flies. We have repeated the entire experiment under strictly controlled conditions and replaced the original quantification with the new, validated results (Fig 1F).

This inconsistent with their response to point 2 above.
I still do not understand how many times experiments were done, how many time it was quantified. This should be easy to find in a paper and not buried.

I also find it slightly concerning that the authors just replaced their original data with new data that have a lower baseline proliferation. There is indeed a lot of variability in experiments done in the adult fly gut in general and I feel that masking the

variability by just replacing it with "better" data is not helpful for the community at large. They should put the original replicate in a supplementary figure as there is no reason to exclude these data.

RNA/ATACseq data sets:

As I mentioned in the first round, I do not believe that they can compare the Psc-Su(z)2 deletion MARCM clones directly to the wild-type esg>GFP cells. See my point 6 on the original review, which they do NOT answer or address, just re-explain to me their rationale. I understand the rationale, but I am suggesting caution in the interpretation of the data. While I agree that this type of comparison could be used for gene discovery (to identify chinmo, that they later validated with immunofluorescence), it is a dangerous overinterpretation to go further in the comparison regarding number of peaks altered, etc. as they have done:

"Notably, following Psc-Su(z)2 deletion,
approximately 3,543 regions of open chromatin became inaccessible, while about
6,196 new regions became accessible, suggesting a global shift toward a more relaxed chromatin state in the mutant cells. To assess whether the observed transcriptomic changes were associated with altered chromatin accessibility, we performed an integrative analysis of RNA-seq and ATAC-seq datasets. Approximately 34.7% of genes upregulated in Psc-Su(z)2-/- cells exhibited significant promoter opening, while 33.1% of downregulated genes were associated with promoter region closure (Fig 4E, Table 2)."

I do not believe that this comparison should be made, or minimally it needs to be qualified.

Minor points:

-How neural-specific are these really? Many are also involved in germline decisions- did they consider a derepression of the germline program? Does GO terms or akin analysis suggest on or the other?

-There is a mis-reference figure here I believe:
Fluorescence-activated cell sorting (FACS)
analysis of gut epithelial cells revealed a significant increase in the proportion of GFP+ cells, from 0.83% in controls to 4.64% following double knockdown of Psc and Su(z)2 (Fig EV1C), indicating accumulation of undifferentiated progenitor cells. Notably, the mean GFP fluorescence intensity in these mutant cells was generally reduced to one-third of the control level-from 39963 to 12054 (Fig EV2C).

Referee #2:

Wei et al. made significant improvements to their revised manuscript with new experiments, improved images, and increased quantifications. However, there is still a significant amount of missing quantifications. Furthermore, the authors were unable to provide additional mechanistic insight into how Psc/Su(z)2 affects differentiation, which limits the impact of the study but could be beyond its scope. Comprehensively addressing the major points below is necessary before the study is suitable for publication.

Major:

1) While the total # of cells and # of PH3+ cells have now been quantified in control versus Su(z)2XL26 mutant clones, quantifications for cell (or nuclear) size and cell density (#cells/area) are missing. Notably, we requested cell size quantification previously. This is important as, throughout the study, the authors describe the phenotype as 'small [cell] clusters/tumors', 'small proliferating cells', and 'densely packed'. All the above quantifications need to be done for Su(z)21.b8, Psc, and Su(z)2 mutants as well.

2) I appreciate the clear differences between the authors' representative images, but all conditions for all experiments (i.e. representative images) must be quantified. These include Sox21a and Sox100b, Chinmo in Psc-Su(z)2 RNAi, #of cells per clone for Chinmo RNAi, Pros and Pdm1 for Chinmo RNAi, GFP+ cells for Fig. 6J-L, and cell fate and signaling assays for Chinmo overexpression. Quantification is critical for proper interpretation of the data, and the lack of quantifications was a major concern across all three reviewers.

3) A number of quantifications were done by calculating %clones with labeled cells per gut (i.e. NRE-LacZ, STAT, pERK, Wnt, and Chinmo). This is not ideal and should be quantified instead by %positively labeled cells/total cells per clone. Resolution is greatly lost with the former strategy as a clone with 50 positively labeled cells would be counted the same as a clone with only one labeled cell. The authors seem to realize this as PH3, Dl, pros, PDM1, tunnel, and H2A are quantified per clone (i.e. %Dl+ cells/total cells per clone). If the authors had quantified Dl, for example, as %of clones with Dl+ cells per gut, both control and Su(z)2XL26 mutant conditions would likely yield no significant difference instead of their observed increase in mutants. While I appreciate that many of the quantifications done in the former way had a drastic difference (i.e. 100% vs <10%), quantifications

should be added if not replaced with %positively labeled cells per clone for improved resolution.

Minor:

4) Stats are missing from several panels, including EV1A , EV2C, and EV2D.

Referee #3:

Overall, this is a much-improved manuscript. However, I have outlined some major and minor revisions that must be completed by the authors. In terms of major revisions, the authors must clarify with data in which cell type (ISC, its progeny or both) give rise to tumors upon the loss of PSc Su(z)2, modify their claims that chinmo drives proliferation in all tumor cells, and provide clearer data supporting their claim that chinmo does not block differentiation. In terms of minor revisions, claims in the Results and Discussion need to be modified for accuracy and the figures need editing. The figures are missing data and contain unclear labels, missing scale bars and labels. Please modify the manuscript as outlined below.

MAJOR:
1. The image of a PSc Su(z)2 mutant clone with multiple PH3-positive cells in Fig. 1F does not represent the average mutant clone. See quantification in EV1C. Although there is a significant difference between control and PSc Su(z)2 mutant clones, most of the mutant clones do not contain multiple PH3 positive cells. The occasional increase observed could arise from clone fusion. In support, the number of mitotic cells per midgut (Figure 1S) is low if all the mutant cells were proliferative. While the effect on differentiation is clear in mutant cells, the authors have not provided as requested definitive data showing that all mutant cells have the capacity to proliferate. Thus, the authors must modify this claim in the manuscript and discuss the possibility that increased chinmo levels may drive proliferation in only a subset of tumor cells and not all tumor cells. It is possible that increased chinmo has other functions than proliferation. The authors should also discuss that increased chinmo levels are not found in all tumor cells and that the levels vary between tumor cells (see Fig. 6B-B').

2. Whether the ISC or EB is affected by the loss of PSc Su(z)2 is not described in the text and these requested data are not included in the manuscript. These data must be included in the manuscript and discussed in the Results and Discussion. From the response, it seems like both the ISC and EB are affected and not only EBs. If only EBs were affected, then ISC clones with normal lineages would be present. The authors claim in the response that ISC lineages are not found. If ISCs were only affected, there would be more EC or EE clones present. On Page 9, the authors similarly assert that that loss of PSc Su(z)2 in ISCs leads to tumor formation. While it could partly be due to ISC loss, ISC progeny (EB, EE precursor) may also fail to differentiate. This should be edited to include ISC progeny as well.

3. Page 9. The authors claim that chinmo doesn't affect differentiation but only proliferation. Although this interpretation seems correct, the data provided to support this claim is not entirely convincing.
a. Fig. 6I. The size of PSc Su(z)2 clones depleted for chinmo are larger by quantitation than shown in Fig. 6G-H and EV Fig. 3A-B'. Please provide images of larger PSc Su(z)2 clones depleted for chinmo in EV Fig. 3A-B so that it is clear that Pdm1- and Pros-positive cells are not found in these clones. Further, the proper number of Pros+ EEs are not visible in the image in EV Fig. 3B'.
b. Please brighten the images in Fig. 6O-P', so the GFP and Gas-LacZ signal is clearly visible. The data clearly show that Notch and Jak-Stat are not affected by chinmo overexpression.
c. Please brighten the images in EV Fig. 41-J, so that Pdm1 and Pros can be observed in chinmo overexpressing GFP+ cells.

MINOR:

1. Revised title. The authors have exchanged the term "mediated" with "dependent", which doesn't change its meaning much. As chinmo promotes proliferation in PSc Su(z)2 mutant tumors and not differentiation, the title could convey this better.
2. Abstract. The authors state Jak-Stat signaling as a ISC proliferation pathway but do not mention its role in ISC differentiation. Please state that the tumors do not activate Jak-Stat and Notch signaling, which are essential for ISC differentiation.
3. Page 4. Please describe in the Results the decrease in PSc Su(z)2 mutant clone size in EV1A. Please indicate if the clone size decrease is significantly different.
4. Page 5. Please describe in the Results the effects of PSc and Su(z)2 expression in Figure 1M and 1N.
5. Page 5. The loss of GFP after knockdown of Psc and Su(z)2 with esg-GAL4 indicates a loss of "progenitor" identity and not ISC identity. Please edit "ISC" to "progenitor".
6. Page 5. If the authors would like to describe that the loss of Notch produces cells that resemble EE precursors, then they must cite Patel et al., 2015 (DOI: 10.1038/ncb3214), which first described that ISC- or progenitor-like Notch mutant cells resemble committed EE precursors. Otherwise, the sentence must be modified.
7. Page 6. The downregulation of factors such as esg, Sox21a, Sox100B, cic, sc and klu suggest a reduction in both ISC and EB (or progenitor) identity rather than solely ISC identity as stated. Please edit "ISC" to "progenitor".
8. Page 9. The clones in EV3C and EV3D are too small to conclude that these cells are not undergoing apoptosis. The authors should modify the sentence to state their data suggests these cells are not undergoing apoptosis.

9. Page 10. The effects of PSc Su(z)2 loss on differentiation, gene expression and chinmo expression compared to other PRC1 complex components is good evidence that both PSc and Su(z)2 partly function independently from the PRC1 complex to maintain ISC identity and suppress tumor formation. However, the H2AK118ub1 and H3K27me3 data do not support this argument. The PSc Su(z)2 mutant cells don't behave differently here from the other PRC1 complex components, except for Sce, which is required for H2AK118ub1. The data, however, do suggest that PSc Su(z)2 do function independently of H2AK118ub to suppress tumor formation. The authors must modify their concluding statement.
10. Page 10. The Sce data is missing from Figure 7R. Please include this data.
11. Discussion Page 10. The authors have investigated the role of PSc Su(z)2 in the "adult" Drosphila midgut. Please include "adult".
12. Discussion Page 10. The authors describe Deadpan as a stem cell factor, but it would be more accurate to describe it as a neural stem cell factor or neuroblast factor. Please edit.
13. Discussion Page 11. The authors state that Chinmo is normally expressed in intestinal epithelial cells, but RNAseq data from Flygut-seq (Dutta et al., 2015) suggests that Chinmo is expressed at low levels. This sentence should be modified to state that the authors could not detect chinmo protein in midgut epithelial cells.
14. Page 12 Discussion. The authors did not examine whether Ph mutant cells can differentiate but claim that they do not. This claim must be modified to state that their data suggest that Ph mutant cells do not differentiate properly.
15. Figure 1F, 2J, 3C, 3F, 3I, 6C the y-axis should state "clones" not clone. The authors should also state per "midgut" rather than per gut since this is the part of the gut they are analysing. This should be edited throughout the manuscript.
16. Please include the scale bar missing from Figure 1E.
17. Please include the DAPI label missing from Figure 1H
18. The Y-axis on Fig 6I, EV Fig 1F should read "clone area". Please edit.
19. Please include the labels DAPI Pros and GFP in Figure 6G and H.
20. Figures EV1D' and E', please brighten these images as it is very difficult to see the Pdm1 signal. Figures EV1D' and E' do not contain the proper number of Pros+ EEs that would normally be found in an area of this size of the midgut epithelium. Please replace with images where the proper number of Pros+ EEs are visible.
21. In EV Fig 1G and H, the y-axes should say "cells" instead of cell. Please edit.
22. EV Fig. 2G' and 4I',J'. The Pros signal is barely detectable, and the number of EEs detected is very low relative to the epithelial area provided in the image. Please replace with images showing the proper number of Pros+ EEs.

Dear Editor and Reviewers,

We sincerely thank you for your second round of careful evaluation of our manuscript and for the constructive and insightful comments. We have carefully considered all suggestions raised by the reviewers and have thoroughly revised the manuscript accordingly. Our point-by-point responses to the reviewers' comments are detailed below.

Point-by-point responses

Reviewers' comments:

Referee #1:

While the authors have addressed a number of important points (adding quantification), there are still points of mine to which they did not respond or said that something was modified, when it was not.

Major points that they did not fully address are regarding their statistics and quantification and their RNA/ATACseq data sets.

Statistics and Quantification:

In the first round, I noted that more detail was required to understand how many times an experiment was performed, how many intestines per replicate, the exact number of clones, etc.

The discussion is reproduced here:

Round 1
My original poin 2 " In the "Statistics" section, it is mentioned that "Each experimental dataset included more than three biological replicates for analysis. All significance tests were analyzed by t tests, mean+/- SEM. Significance P values: nsp > 0.05, *p < 0.05,
**p < 0.01, ***p < 0.001, ****p < 0.0001." The authors need to give more detailed information per figure on what was done and what data is shown. For example, the n= for each quantification should be indicated on the graph. Many points are on top of each other and we cannot see simply from the graph. If we look at Figure 1F, the control has 10 data points and the mutant 11. Was this the pooled data of the 3 biological replicates? Is this just one replicate? If so, where are the other data?"
Authors "In the revised manuscript, the statistical plots now explicitly indicate the number of biological replicates, and all additional experimental details have been

incorporated into the corresponding figure legends."
I am very confused. The statistics plots do NOT seem to indicate the number of biological replicates. For example, in 1F, they plot the number of midguts, not the number of biological replicates.

We apologize for the confusion made regarding the experimental replicates and statistical presentation.

The samples analyzed in this experiment were all obtained from three independent biological replicates, which were established from separate sets of flies.

Across these three biological replicates, 5 or more midguts have been collected and analyzed per group, and the data points shown in the figures represent total midguts combined.

We have labeled the total number of individual midguts (n) in each graph and have explicitly noted N = 3 biological replicates in the revised figure legends to avoid confusion.

Similarly, in point 4 from my original review:
4. What is being quantified exactly in Figure 1F? What does each point represent? A midgut? I find it surprising that 20% of control clones have a Ph3+ cell.
This inconsistent with their response to point 2 above.
I still do not understand how many times experiments were done, how many time it was quantified. This should be easy to find in a paper and not buried.
I also find it slightly concerning that the authors just replaced their original data with new data that have a lower baseline proliferation. There is indeed a lot of variability in experiments done in the adult fly midgut in general and I feel that masking the variability by just replacing it with "better" data is not helpful for the community at large. They should put the original replicate in a supplementary figure as there is no reason to exclude these data.

We appreciate the reviewer's thoughtful comments and concern regarding data transparency and experimental variability.

For the data points shown in Figure 1F, each represents an individual midgut, and we quantified the proportion of clones containing at least one pH3-positive cell. All quantifications were performed across three independent biological replicates. In the revised figure and corresponding legend, we have indicated

both the number of individual midguts (n) and the number of biological replicates (N = 3) to avoid confusion.

Regarding the original dataset with a higher baseline proliferation rate, we acknowledge that variability is common in the adult *Drosophila* midgut. Nevertheless, we found that differences between control and experimental groups were consistently apparent and statistically significant across different experimental sets, despite substantial variation in the baseline proliferation rate. The elevated baseline level observed in our initial experiment, conducted some time ago, likely arose from unexpected midgut stresses that occurred during the 14-day room-temperature incubation period following the heat shock treatment.

In response to the reviewer's suggestion, we have added the original dataset as a supplementary Fig EV1E, accompanied by a detailed figure legend explaining the likely cause of the baseline variability. This supplementary figure includes the original quantification data, ensuring full transparency without merging distinct datasets. We believe this presentation allows readers to appreciate both the physiological baseline and the system's sensitivity to the environmental conditions. Furthermore, it provides additional evidence supporting an increased proliferation rate in the experimental group, even the baseline activity of the control group is elevated.

RNA/ATACseq data sets:
As I mentioned in the first round, I do not believe that they can compare the Psc-Su(z)2 deletion MARCM clones directly to the wild-type esg>GFP cells. See my point 6 on the original review, which they do NOT answer or address, just re-explain to me their rationale. I understand the rationale, but I am suggesting caution in the interpretation of the data. While I agree that this type of comparison could be used for gene discovery (to identify chinmo, that they later validated with immunofluorescence), it is a dangerous overinterpretation to go further in the comparison regarding number of peaks altered, etc. as they have done:
"Notably, following Psc-Su(z)2 deletion,
approximately 3,543 regions of open chromatin became inaccessible, while about 6,196 new regions became accessible, suggesting a global shift toward a more relaxed chromatin state in the mutant cells. To assess whether the observed transcriptomic changes were associated with altered chromatin accessibility, we performed an integrative analysis of RNA-seq and ATAC-seq datasets. Approximately 34.7% of genes upregulated in Psc-Su(z)2-/- cells exhibited significant promoter opening, while 33.1% of downregulated genes were associated with promoter region closure (Fig 4E, Table 2)."

I do not believe that this comparison should be made, or minimally it needs to be qualified.

We agree that mutant MARCM clones and control esg>GFP cells were isolated using distinct genetic systems, which may introduce differences beyond the specific mutation of interest.

As the reviewer pointed out, these datasets were primarily employed for candidate gene discovery, which successfully identified chinmo as a key target, followed by experimental validation via immunofluorescence.

In the revised manuscript, we have only subjectively presented the genomic profiles observed in our ATAC-seq and RNA-seq analyses. We acknowledge that these observations should be interpreted as descriptive trends rather than rigorous quantitative comparisons or definitive conclusions. We have carefully revised the relevant descriptions to soften the tone and avoid overinterpretation, in accordance with the reviewer's concern.

Minor points:

-How neural-specific are these really? Many are also involved in germline decisions- did they consider a derepression of the germline program? Does GO terms or akin analysis suggest on or the other?

It has been previously reported that certain fly tumor models, such as *l(3)mbt* mutant *Drosophila* brain tumors, undergo a soma-to-germline transformation and ectopically activate a broad suite of germline-specific genes, including *piwi*, *vasa*, *nanos*, and among others (Janic et al., 2010; PMID: 21205669). In our study, we did not detect significant upregulation of these germline-specific genes in our tumor model, nor did our GO analyses indicate widespread derepression of the germline transcriptional program.

-There is a mis-reference figure here I believe:
Fluorescence-activated cell sorting (FACS)
analysis of midgut epithelial cells revealed a significant increase in the proportion of GFP+ cells, from 0.83% in controls to 4.64% following double knockdown of Psc and Su(z)2 (Fig EV1C), indicating accumulation of undifferentiated progenitor cells. Notably, the mean GFP fluorescence intensity in these mutant cells was generally reduced to one-third of the control level-from 39963 to 12054 (Fig EV2C).

Corrected, and thank you for catching this error (citation changed from Fig EV1C to Fig EV2C.

Referee #2:

Wei et al. made significant improvements to their revised manuscript with new experiments, improved images, and increased quantifications. However, there is still a significant amount of missing quantifications. Furthermore, the authors were unable to provide additional mechanistic insight into how Psc/Su(z)2 affects differentiation, which limits the impact of the study but could be beyond its scope. Comprehensively addressing the major points below is necessary before the study is suitable for publication.

Major:

1) While the total # of cells and # of PH3+ cells have now been quantified in control versus Su(z)2XL26 mutant clones, quantifications for cell (or nuclear) size and cell density (#cells/area) are missing. Notably, we requested cell size quantification previously. This is important as, throughout the study, the authors describe the phenotype as 'small [cell] clusters/tumors', 'small proliferating cells', and 'densely packed'. All the above quantifications need to be done for Su(z)21.b8, Psc, and Su(z)2 mutants as well.

We thank the reviewer for this valuable comment.

As requested, we have performed quantitative analysis of cell density (number of cells per clone area) for *Su(z)2^{XL26}*, *Su(z)2$^{1.b8}$*, *Psc*, and *Su(z)2* mutant clones and have included these data in the revised manuscript.

Regarding nuclear size quantification, we observed that nuclear size in wild-type clones is highly heterogeneous, and nuclear sizes differ significantly among ISCs, EBs and ECs, which would complicate quantitative interpretation and limit the biological significance of such an analysis. We therefore measured nuclear sizes only for wild-type ISCs and for tumor cells in mutant clones. The nuclear size of mutant cells is approximately 20 μm², similar to that of ISCs, consistent with their appearance as small progenitor-like cells.

2) I appreciate the clear differences between the authors' representative images, but all conditions for all experiments (i.e. representative images) must be quantified. These include Sox21a and Sox100b, Chinmo in Psc-Su(z)2 RNAi, #of cells per clone for *Chinmo RNAi,* Pros and Pdm1 for *Chinmo RNAi*, GFP+ cells for Fig. 6J-L, and cell fate and signaling assays for Chinmo overexpression. Quantification is critical for

proper interpretation of the data, and the lack of quantifications was a major concern across all three reviewers.

We have now added quantitative data for all conditions across all experiments, including Sox21a, Sox100b in *Psc-Su(z)2* mutant clones, Chinmo in *Psc-Su(z)2* RNAi clones, cell number per clone, and Pros and Pdm1 in *Chinmo* RNAi clones, as well as cell fate and signaling readouts for the relevant genotypes.

For the GFP^{+} cells shown in Figures 6J–L and the reporter assays under Chinmo overexpression conditions, the extensive cellular proliferation led to excessive clustering and fusion of GFP signals, making individual cell counting unreliable. For these specific readouts, we instead quantified total fluorescent signal area within a standardized, matched region of the same midgut segment (R4a) across all samples to ensure consistent and reproducible quantification.

All quantification data, including these supplementary analyses, are presented in the revised manuscript as new panels in the main and/or supplementary figures to fully support our conclusions.

3) A number of quantifications were done by calculating %clones with labeled cells per midgut (i.e. NRE-LacZ, STAT, pERK, Wnt, and Chinmo). This is not ideal and should be quantified instead by %positively labeled cells/total cells per clone. Resolution is greatly lost with the former strategy as a clone with 50 positively labeled cells would be counted the same as a clone with only one labeled cell. The authors seem to realize this as PH3, DI, pros, PDM1, tunnel, and H2A are quantified per clone (i.e. %DI+ cells/total cells per clone). If the authors had quantified DI, for example, as %of clones with DI+ cells per midgut, both control and Su(z)2XL26 mutant conditions would likely yield no significant difference instead of their observed increase in mutants. While I appreciate that many of the quantifications done in the former way had a drastic difference (i.e. 100% vs <10%), quantifications should be added if not replaced with %positively labeled cells per clone for improved resolution.

We agree with the reviewer that quantification at the clone level (% positive cells/total cells per clone) would provide a much higher resolution. We have updated the analyses for NRE-LacZ, STAT, pERK, Wnt, and Chinmo with this approach, similar to the analyses performed for other markers, and included these quantitative data in the revised figures.

Minor:
4) Stats are missing from several panels, including EV1A, EV2C, and EV2D.
Statistics have been added for EV2C and EV2D. EV1A shows the flow sorting result, which was performed only once.

Referee #3:

Overall, this is a much-improved manuscript. However, I have outlined some major and minor revisions that must be completed by the authors. In terms of major revisions, the authors must clarify with data in which cell type (ISC, its progeny or both) give rise to tumors upon the loss of PSc Su(z)2, modify their claims that chinmo drives proliferation in all tumor cells, and provide clearer data supporting their claim that chinmo does not block differentiation. In terms of minor revisions, claims in the Results and Discussion need to be modified for accuracy and the figures need editing. The figures are missing data and contain unclear labels, missing scale bars and labels. Please modify the manuscript as outlined below.

MAJOR:

1. The image of a PSc Su(z)2 mutant clone with multiple PH3-positive cells in Fig. 1F does not represent the average mutant clone. See quantification in EV1C. Although there is a significant difference between control and PSc Su(z)2 mutant clones, most of the mutant clones do not contain multiple PH3 positive cells. The occasional increase observed could arise from clone fusion. In support, the number of mitotic cells per midmidgut (Figure 1S) is low if all the mutant cells were proliferative. While the effect on differentiation is clear in mutant cells, the authors have not provided as requested definitive data showing that all mutant cells have the capacity to proliferate. Thus, the authors must modify this claim in the manuscript and discuss the possibility that increased chinmo levels may drive proliferation in only a subset of tumor cells and not all tumor cells. It is possible that increased chinmo has other functions than proliferation. The authors should also discuss that increased chinmo levels are not found in all tumor cells and that the levels vary between tumor cells (see Fig. 6B-B').

We appreciate the reviewer's careful analysis of our data and their thoughtful suggestions.

PH3 labeling only reveals mitotic cells within a narrow window of the cell cycle and therefore has inherent limitations for assessing the full proliferative potential of a cell population. Based on the newly added data on cell number per clone (Fig EV1D), it is clear that the tumor cells are highly proliferative at the population level compared to wild-type ISCs. The reviewer raises a good point: we do not know whether all tumor cells can proliferate or only a subset of them. Indeed, although most tumor cells express Chinmo, the expression level is heterogeneous among tumor cells within a clone (Figures 6B–B'), raising the possibility that only a subset of tumor cells drives proliferation. We have revised the manuscript to acknowledge these points.

2. Whether the ISC or EB is affected by the loss of PSc Su(z)2 is not described in the text and these requested data are not included in the manuscript. These data must be

included in the manuscript and discussed in the Results and Discussion. From the response, it seems like both the ISC and EB are affected and not only EBs. If only EBs were affected, then ISC clones with normal lineages would be present. The authors claim in the response that ISC lineages are not found. If ISCs were only affected, there would be more EC or EE clones present. On Page 9, the authors similarly assert that that loss of PSc Su(z)2 in ISCs leads to tumor formation. While it could partly be due to ISC loss, ISC progeny (EB, EE precursor) may also fail to differentiate. This should be edited to include ISC progeny as well.

We thank the reviewer for this important comment regarding the cell-type specificity of the tumor-suppressive function of Psc-Su(z)2.

To address whether defects arise in ISCs, EBs, or both, we performed cell-type-specific knockdown using ISC-specific Dl-Gal4 and EB-specific Su(H)-Gal4 drivers. These data, included in Fig EV5, demonstrate that loss of Psc-Su(z)2 in EBs is sufficient to cause cells to re-enter the cell cycle, and prevent differentiation. Therefore, in addition to ISCs, EBs also contribute to tumorigenesis following the loss of Psc-Su(Z)2.

Based on these new results, we have revised the main text and discussion to clarify that loss of *Psc-Su(z)2* in EBs can also prevent differentiation and promote cell cycle re-entry, thereby contributing to tumorigenesis.

3. Page 9. The authors claim that chinmo doesn't affect differentiation but only proliferation. Although this interpretation seems correct, the data provided to support this claim is not entirely convincing.
a. Fig. 6I. The size of PSc Su(z)2 clones depleted for chinmo are larger by quantitation than shown in Fig. 6G-H and EV Fig. 3A-B'. Please provide images of larger PSc Su(z)2 clones depleted for chinmo in EV Fig. 3A-B so that it is clear that Pdm1- and Pros-positive cells are not found in these clones. Further, the proper number of Pros+ EEs are not visible in the image in EV Fig. 3B'.

We thank the reviewer for their careful evaluation of our data supporting the role of Chinmo in proliferation rather than differentiation.

Regarding the quantification showing that Psc-Su(z)2 mutant clones depleted for *chinmo* are larger than the representative images previously displayed, we have replaced the original Fig. 6H with a new panel showing representative *Psc-Su(z)2* mutant clones with *chinmo* knockdown of varying sizes (ranging from ~90 to ~463 μm²). The corresponding clone areas are also described in the updated figure legend.

Of note, even in these larger clones, Pdm1⁺ or Pros⁺ cells remain absent, supporting our hypothesis that ectopic activation of Chinmo promotes tumor cell proliferation but is not responsible for the differentiation blockage. We have also adjusted the presentation of Pros⁺ cells to improve their visibility.

We noticed that the larger clones are usually associated with incomplete knockdown efficiency of *chinmo RNAi* (incomplete elimination of anti-Chinmo signal in the clone), and we have therefore interpreted these as rare outliers. Overall, our quantification confirms that *chinmo knockdown* significantly reduces clone size, but fails to rescue defects in EE or EC differentiation.

b. Please brighten the images in Fig. 6O-P', so the GFP and Gas-LacZ signal is clearly visible. The data clearly show that Notch and Jak-Stat are not affected by chinmo overexpression.

Done as suggested

c. Please brighten the images in EV Fig. 41-J, so that Pdm1 and Pros can be observed in chinmo overexpressing GFP+ cells.

Done as suggested.

MINOR:

1. Revised title. The authors have exchanged the term "mediated" with "dependent", which doesn't change its meaning much. As chinmo promotes proliferation in PSc Su(z)2 mutant tumors and not differentiation, the title could convey this better.

We appreciate the reviewer's comment. However, we were unable to identify a more suitable alternative to "mediated" than "dependent." Chinmo is required but not sufficient for tumorigenesis. In our view, "mediated" can imply a mechanism that is both necessary and sufficient, whereas "dependent" more accurately reflects only a requirement for Chinmo. We would be grateful for any further suggestions the reviewer may have to improve this wording.

2. Abstract. The authors state Jak-Stat signaling as a ISC proliferation pathway but do not mention its role in ISC differentiation. Please state that the tumors do not activate Jak-Stat and Notch signaling, which are essential for ISC differentiation.

Done as suggested.

3. Page 4. Please describe in the Results the decrease in PSc Su(z)2 mutant clone size in EV1A. Please indicate if the clone size decrease is significantly different.

Done as suggested.

4. Page 5. Please describe in the Results the effects of PSc and Su(z)2 expression in Figure 1M and 1N.

Done as suggested.

5. Page 5. The loss of GFP after knockdown of Psc and Su(z)2 with esg-GAL4 indicates a loss of "progenitor" identity and not ISC identity. Please edit "ISC" to "progenitor".

Done as suggested.

6. Page 5. If the authors would like to describe that the loss of Notch produces cells that resemble EE precursors, then they must cite Patel et al., 2015 (DOI: 10.1038/ncb3214), which first described that ISC- or progenitor-like Notch mutant cells resemble committed EE precursors. Otherwise, the sentence must be modified.

Done as suggested.

7. Page 6. The downregulation of factors such as esg, Sox21a, Sox100B, cic, sc and klu suggest a reduction in both ISC and EB (or progenitor) identity rather than solely ISC identity as stated. Please edit "ISC" to "progenitor".

Done as suggested.

8. Page 9. The clones in EV3C and EV3D are too small to conclude that these cells are not undergoing apoptosis. The authors should modify the sentence to state their data **suggests** these cells are not undergoing apoptosis.

Done as suggested.

9. Page 10. The effects of PSc Su(z)2 loss on differentiation, gene expression and chinmo expression compared to other PRC1 complex components is good evidence that both PSc and Su(z)2 partly function independently from the PRC1 complex to maintain ISC identity and suppress tumor formation. However, the H2AK118ub1 and H3K27me3 data do not support this argument. The PSc Su(z)2 mutant cells don't behave differently here from the other PRC1 complex components, except for Sce, which is required for H2AK118ub1. The data, however, do suggest that PSc Su(z)2 do function independently of H2AK118ub to suppress tumor formation. The authors must modify their concluding statement.

Done as suggested.

10. Page 10. The Sce data is missing from Figure 7R. Please include this data.

The data presented in Figure 7R were adopted from the previously published study by Tauc et al. Since quantitative data for Sce are not available in the original published dataset, we are unable to include or supplement these values in the figure.

11. Discussion Page 10. The authors have investigated the role of PSc Su(z)2 in the "adult" Drosphila midmidgut. Please include "adult".

Done as suggested.

12. Discussion Page 10. The authors describe Deadpan as a stem cell factor, but it would be more accurate to describe it as a neural stem cell factor or neuroblast factor. Please edit.

Done as suggested.

13. Discussion Page 11. The authors state that Chinmo is normally expressed in intestinal epithelial cells, but RNAseq data from Flymidgut-seq (Dutta et al., 2015) suggests that Chinmo is expressed at low levels. This sentence should be modified to state that the authors could not detect chinmo protein in midmidgut epithelial cells.

Done as suggested.

14. Page 12 Discussion. The authors did not examine whether Ph mutant cells can differentiate but claim that they do not. This claim must be modified to state that their data suggest that Ph mutant cells do not differentiate properly.

Done as suggested.

15. Figure 1F, 2J, 3C, 3F, 3I, 6C the y-axis should state "clones" not clone. The authors should also state per "midmidgut" rather than per midgut since this is the part of the midgut they are analysing. This should be edited throughout the manuscript.

Done as suggested.

16. Please include the scale bar missing from Figure 1E.

Done as suggested.

17. Please include the DAPI label missing from Figure 1H

Done as suggested.

18. The Y-axis on Fig 6I, EV Fig 1F should read "clone area". Please edit.

Done as suggested.

19. Please include the labels DAPI Pros and GFP in Figure 6G and H.

Done as suggested.

20. Figures EV1D' and E', please brighten these images as it is very difficult to see the Pdm1 signal. Figures EV1D' and E' do not contain the proper number of Pros+ EEs that would normally be found in an area of this size of the midmidgut epithelium. Please replace with images where the proper number of Pros+ EEs are visible.

Done as suggested.

21. In EV Fig 1G and H, the y-axes should say "cells" instead of cell. Please edit.

Done as suggested.

22. EV Fig. 2G' and 4I',J'. The Pros signal is barely detectable, and the number of EEs detected is very low relative to the epithelial area provided in the image. Please replace with images showing the proper number of Pros+ EEs.

Done as suggested.

2nd Revision - Editorial Decision 20th May 2026

Dear Dr. Xi,

Thank you for the submission of your further revised manuscript to our editorial offices. I have now received the report from the three referees that were asked to re-evaluate the study, you will find below. As you will see, the referees now support the publication of the study in EMBO reports. However, the referees #1 and #3 have remaining concerns and suggestions to improve the manuscript or requests for clarification. Please address these in a final revised manuscript and a final p-b-p-response.

Moreover, I have some further editorial requests:

- Please provide a final title with not more than 100 characters (including spaces).

- Please provide complete statistics (n.s.) for panel 5C.

- The writing in the synopsis image when sized to the requested dimension (exact width of 550 pixels and the exact height of 300 pixels) - see attached - is too small. Part of it in the central panel can't be read as it overlaps with the design. Please improve this, stick to the requested dimension.

I look forward to seeing the final revised version of your manuscript when it is ready. Please let me know if you have questions regarding the revision.

Best,

Achim Breiling
Senior Editor
EMBO Reports

Referee #1:

The authors have more or less fixed my main points. There are still issues that I do not understand- they state : "Across these three biological replicates, 5 or more midguts have been collected and analyzed per group, and the data points shown in the figures represent total midguts combined. We have labeled the total number of individual midguts (n) in each graph and have explicitly noted N = 3 biological replicates in the revised figure legends to avoid confusion"; however they have n=6. Does this mean that they only analyzed 2 guts per biological repeat? To me, this is quite low, but fine.

Secondly, they claim that they: "n response to the reviewer's suggestion, we have added the original dataset as a supplementary Fig EV1E, accompanied by a detailed figure legend explaining the likely cause of the baseline variability. This supplementary figure includes
the original quantification data, ensuring full transparency without merging distinct datasets. We believe this presentation allows readers to appreciate both the physiological baseline and the system's sensitivity to the environmental conditions. Furthermore, it provides additional evidence supporting an increased proliferation rate in the experimental group, even the baseline activity of the control group is elevated."

However, I do not see this "detailed figure legend" explaining the potential causes of variability. It seems to me that one author wrote the responses and another one was supposed to implement the changes and that these did not get done. Nevertheless, there are useful data here and it should be published.

Referee #2:

The authors did a good job improving the manuscript. I am satisfied with their revisions and I believe the manuscript is suitable for publication.

Referee #3:

The authors have revised their manuscript to address most of my concerns. Nevertheless, I have few outstanding major and minor concerns with the manuscript. The authors may already have the data to address these concerns, and thus it should in

principle not require more experimentation.

Major:

1. The authors have examined the role of Psc Su(z)2 in ISCs and EBs by expressing RNAis towards Psc Su(z)2 with Dl-GAL4 and Su(H)-GAL4, respectively. The authors claim (page 10) that ISC-specific knockdown of Psc Su(z)2 caused ISC proliferation and blocked differentiation into either ECs or EEs. However, the data presented in Fig. EV5B-B' do not demonstrate increased ISC proliferation since they only present a field with two PH3-positive (mitotic) cells. The authors should quantify the number of PH3-positive cells in the whole midgut or at least within the posterior midgut where the RNAi shows strongest effects. Expression of RNAi towards Psc Su(z)2 with Dl-GAL4 does seem to sometimes produce small cell clusters (Fig. EV5B); however, it's not clear whether these cells fail to differentiate (Fig. EV5D-D') or upregulate Chinmo (Fig. EV5J-J") like Psc Su(z)2 mutants. In Fig. EV5D-D', most of Dl>GFP+ cells don't form clusters (tumours), and it seems like some of these cells are Pdm1+. In Fig. 5V5J-J", many of the Dl>GFP+ cells are not Chinmo-positive, whilst others are positive but Dl>GFP-. The authors should provide a field where the Dl+ cells form clusters of small cells that are mostly Chinmo-positive and Pdm1-negative.

The authors claim that Psc Su(z)2 knockdown in Su(H)+ EBs caused them to reenter the cell cycle, remain undifferentiated and accumulate in the epithelium. They also claim that Chinmo is derepressed in EBs upon Psc Su(z)2 depletion. However, the accumulating GFP-positive cells are not small cells, and they are not Pdm1+, suggesting that these Su(H)+ EBs accumulate and do not differentiate into ECs. Furthermore, these Su(H)+ EBs are not Chinmo-positive but the surrounding cells are Chinmo-positive. The PH3 observed in Fig. EV5F-F' is not within Su(H)>GFP+ cells, thus the increased PH3 and Chinmo could be from a non-autonomous effect from the accumulation of these cells. The authors need to modify their claims.

Alternatively, the authors could also argue whether Psc Su(z)2 is required in ISCs or their progeny or both by quantifying the types of clones produced after Psc Su(z)2 loss. They could describe how many clones are tumour-like or ISC clones or progeny clones. If only ISC progeny were affected, then ISC clones with normal lineages would be present amongst tumours. If ISCs were only affected, then about half the clones would be single cell EC or EE clones. If only (or mostly) tumour-like clones are found, then Psc Su(z)2 loss affects both the ISC and its progeny.

The authors also have the option to conclude that Psc Su(z)2 is required within esg+ midgut progenitors to suppress tumour formation. If so, the title, abstract and text should reflect the effects of Psc Su(z)2 loss on ISC progeny too.

2. Fig. EV4Q-R: if Chinmo overexpression doesn't affect differentiation, then why do Pdm1+ and Pros+ cells decrease in the epithelium? In EV4P, the Pdm1+ and Pros+ signal is outside of the GFP+ area. Thus, the data provided do not prove that differentiation is not blocked in these cells. Quantification of fields with a high percentage of GFP+ cells should be able to address this better. Currently, the data does not support the conclusion that Chinmo does not affect differentiation. Thus, the authors have the option to modify this claim or to provide more convincing data.

Minor:

3. Abstract: "ISC identity genes" should be modified to ISC and progenitor identity genes since many of these genes are found in ISC progeny as well.
4. DAPI labels in fluorescent panels are difficult to see. Please adjust colour to make them more visible.
5. Figure 4C: esg should start with a lowercase e.
6. Fig. EV4M: label on x-axis is floating between panels M and N.
7. dpERK seems to decrease in Psc Su(z)2 clones (Fig. 2F) but increases after Chino overexpression (Fig. EV4F). The authors could discuss this discrepancy.
8. Title on page 6: "profiles" is a vague term. Perhaps "cells with neural-like gene expression"?
9. Page 7: Dl is considered an ISC-specific marker, thus the downregulated genes are "key intestinal ISC- or progenitors-specific markers".
10. Page 8: The title starting with "Comparative transcriptome analysis..." could describe the conclusion of this section. Perhaps a title that describes how Chinmo is required for Psc Su(z)2 tumour growth?
11. Page 8: Chinmo is expressed rather than activated. Please modify.
12. Page 10: the authors must modify their statement about H3K27me3 in PRC1 subunit clones. The authors state that "H3K27me3 levels were unaffected by Psc Su(z)2 deletion, nor by mutations in other PRC1 subunits" (Page 10). However, H3K27me3 does seem to be affected in sce or pc mutant clones.
13. The figure legends should include the number of clones, midguts etc., the number of experiments but also how many experiments are presented. The legend for Fig. 1F says that this experiment was performed 3 times, but data from only 6 midguts are shown. It seems like this data is from one of three experiments. Please clarify for all experiments.

Point-by-point responses:

Referee #1:

The authors have more or less fixed my main points. There are still issues that I do not understand- they state : "Across these three biological replicates, 5 or more midguts have been collected and analyzed per group, and the data points shown in the figures represent total midguts combined. We have labeled the total number of individual midguts (n) in each graph and have explicitly noted N = 3 biological replicates in the revised figure legends to avoid confusion"; however they have n=6. Does this mean that they only analyzed 2 guts per biological repeat? To me, this is quite low, but fine.

Yes. Two midguts were examined per biological replicate, with a total of n = 6 midguts from N = 3 independent experiments. The phenotypes were robust and highly reproducible, so the sample size was sufficient for statistical significance.

Secondly, they claim that they: "n response to the reviewer's suggestion, we have added the original dataset as a supplementary Fig EV1E, accompanied by a detailed figure legend explaining the likely cause of the baseline variability. This supplementary figure includes the original quantification data, ensuring full transparency without merging distinct datasets. We believe this presentation allows readers to appreciate both the physiological baseline and the system's sensitivity to the environmental conditions. Furthermore, it provides additional evidence supporting an increased proliferation rate in the experimental group, even the baseline activity of the control group is elevated."

However, I do not see this "detailed figure legend" explaining the potential causes of variability. It seems to me that one author wrote the responses and another one was supposed to implement the changes and that these did not get done.

We sincerely apologize for this confusion and oversight. As noted in our previous response, we have now revised and expanded both figure legends to include detailed explanations for the observed baseline variability.

Nevertheless, there are useful data here and it should be published.

We thank the reviewer for these helpful suggestions, which have greatly improved the clarity of the manuscript.

Referee #2:

The authors did a good job improving the manuscript. I am satisfied with their revisions and I believe the manuscript is suitable for publication.

We greatly appreciate the reviewer's positive feedback and kind approval of our manuscript. Thank you for your time and insightful comments.

Referee #3:

The authors have revised their manuscript to address most of my concerns. Nevertheless, I have few outstanding major and minor concerns with the manuscript. The authors may already have the data to address these concerns, and thus it should in principle not require more experimentation.

Major:

1. The authors have examined the role of Psc Su(z)2 in ISCs and EBs by expressing RNAis towards Psc Su(z)2 with Dl-GAL4 and Su(H)-GAL4, respectively. The authors claim (page 10) that ISC-specific knockdown of Psc Su(z)2 caused ISC proliferation and blocked differentiation into either ECs or EEs. However, the data presented in Fig. EV5B-B' do not demonstrate increased ISC proliferation since they only present a field with two PH3-positive (mitotic) cells. The authors should quantify the number of PH3-positive cells in the whole midgut or at least within the posterior midgut where the RNAi shows strongest effects. Expression of RNAi towards Psc Su(z)2 with Dl-GAL4 does seem to sometimes produce small cell clusters (Fig. EV5B); however, it's not clear whether these cells fail to differentiate (Fig. EV5D-D') or upregulate Chinmo (Fig. EV5J-J'') like Psc Su(z)2 mutants. In Fig. EV5D-D', most of Dl>GFP+ cells don't form clusters (tumours), and it seems like some of these cells are Pdm1+. In Fig. 5V5J-J'', many of the Dl>GFP+ cells are not Chinmo-positive, whilst others are positive but Dl>GFP-. The authors should provide a field where the Dl+ cells form clusters of small cells that are mostly Chinmo-positive and Pdm1-negative.

We sincerely appreciate the reviewer's careful and critical evaluation of our cell-type-specific knockdown results.

Regarding the proliferation and differentiation phenotypes induced by Dl-GAL4-driven Psc-Su(z)2 knockdown in ISCs, we have now quantified PH3-positive mitotic cells across the entire midgut (see Figure EV5C and EV5H). We

acknowledge that Dl-GAL4 has relatively weaker transcriptional activity than esg-GAL4, which leads to a milder tumor phenotype. This attenuated phenotype is also evidenced by the sporadic presence of Dl$^+$Pdm1$^+$ double-positive cells, suggesting partial progression toward cell differentiation. Notably, accumulation of undifferentiated cells remains the dominant phenotype.

We have replaced the images for Figure EV5J–J'' (now Figure EV5L) to better visualize the small cell cluster phenotype. The new panels display tight clusters of small Dl>GFP$^+$ cells predominantly expressing Chinmo. These data confirm that ISC-specific Psc-Su(z)2 knockdown induces ectopic Chinmo expression and promotes small tumor-like clusters, consistent with the phenotype of Psc-Su(z)2 mutant clones.

The authors claim that Psc Su(z)2 knockdown in Su(H)+ EBs caused them to reenter the cell cycle, remain undifferentiated and accumulate in the epithelium. They also claim that Chinmo is derepressed in EBs upon Psc Su(z)2 depletion. However, the accumulating GFP-positive cells are not small cells, and they are not Pdm1+, suggesting that these Su(H)+ EBs accumulate and do not differentiate into ECs. Furthermore, these Su(H)+ EBs are not Chinmo-positive but the surrounding cells are Chinmo-positive. The PH3 observed in Fig. EV5F-F' is not within Su(H)>GFP+ cells, thus the increased PH3 and Chinmo could be from a non-autonomous effect from the accumulation of these cells. The authors need to modify their claims.

Please note that Psc and Su(z)2 are essential for maintaining ISC/EB identity and sustaining Notch pathway activity. Upon Su(H)-GAL4-mediated knockdown of Psc-Su(z)2 in EBs, cells with initial Notch activity rapidly lose their identity and Notch signaling, resulting in decreased GFP expression. This likely accounts for the apparent lack of colocalization between Su(H)-GFP and Chinmo signals. We have added relevant explanations in the main text to clarify this imperfect colocalization.

Alternatively, the authors could also argue whether Psc Su(z)2 is required in ISCs or their progeny or both by quantifying the types of clones produced after Psc Su(z)2 loss. They could describe how many clones are tumour-like or ISC clones or progeny clones. If only ISC progeny were affected, then ISC clones with normal lineages would be present amongst tumours. If ISCs were only affected, then about half the clones would be single cell EC or EE clones. If only (or mostly) tumour-like clones

are found, then Psc Su(z)2 loss affects both the ISC and its progeny.

Since we exclusively observed a single type of small cell cluster in Psc Su(z)2 mutant clones, it is reasonable to conclude that loss of Psc and Su(z)2 function in either ISCs or EBs can equally drive the formation of small cell tumors. This conclusion is further supported by results from cell-type-specific knockdown assays using Dl-GAL4 and Su(H)-GAL4.

The authors also have the option to conclude that Psc Su(z)2 is required within esg+ midgut progenitors to suppress tumour formation. If so, the title, abstract and text should reflect the effects of Psc Su(z)2 loss on ISC progeny too.

We have integrated this information into the revised abstract. We kept the original title to preserve the core conclusions of our study and stay within the word count restrictions.

2. Fig. EV4Q-R: if Chinmo overexpression doesn't affect differentiation, then why do Pdm1+ and Pros+ cells decrease in the epithelium? In EV4P, the Pdm1+ and Pros+ signal is outside of the GFP+ area. Thus, the data provided do not prove that differentiation is not blocked in these cells. Quantification of fields with a high percentage of GFP+ cells should be able to address this better. Currently, the data does not support the conclusion that Chinmo does not affect differentiation. Thus, the authors have the option to modify this claim or to provide more convincing data.

We thank the reviewer for carefully evaluating the differentiation phenotype caused by Chinmo overexpression. The decline in Pdm1^{+} ECs and Pros^{+} EEs is likely due to the extensive ISC proliferation induced by Chinmo overexpression. The expanded progenitor population occupies tissue volume and reduces the relative fraction of terminally differentiated cells.

Supporting this idea, we consistently observed weakly stained GFP^{+} Pdm1^{+} double-positive cells (marked by white arrows in Fig EV4P, see below the cropped image), these cells are likely newly differentiated ECs with lingering GFP expression.

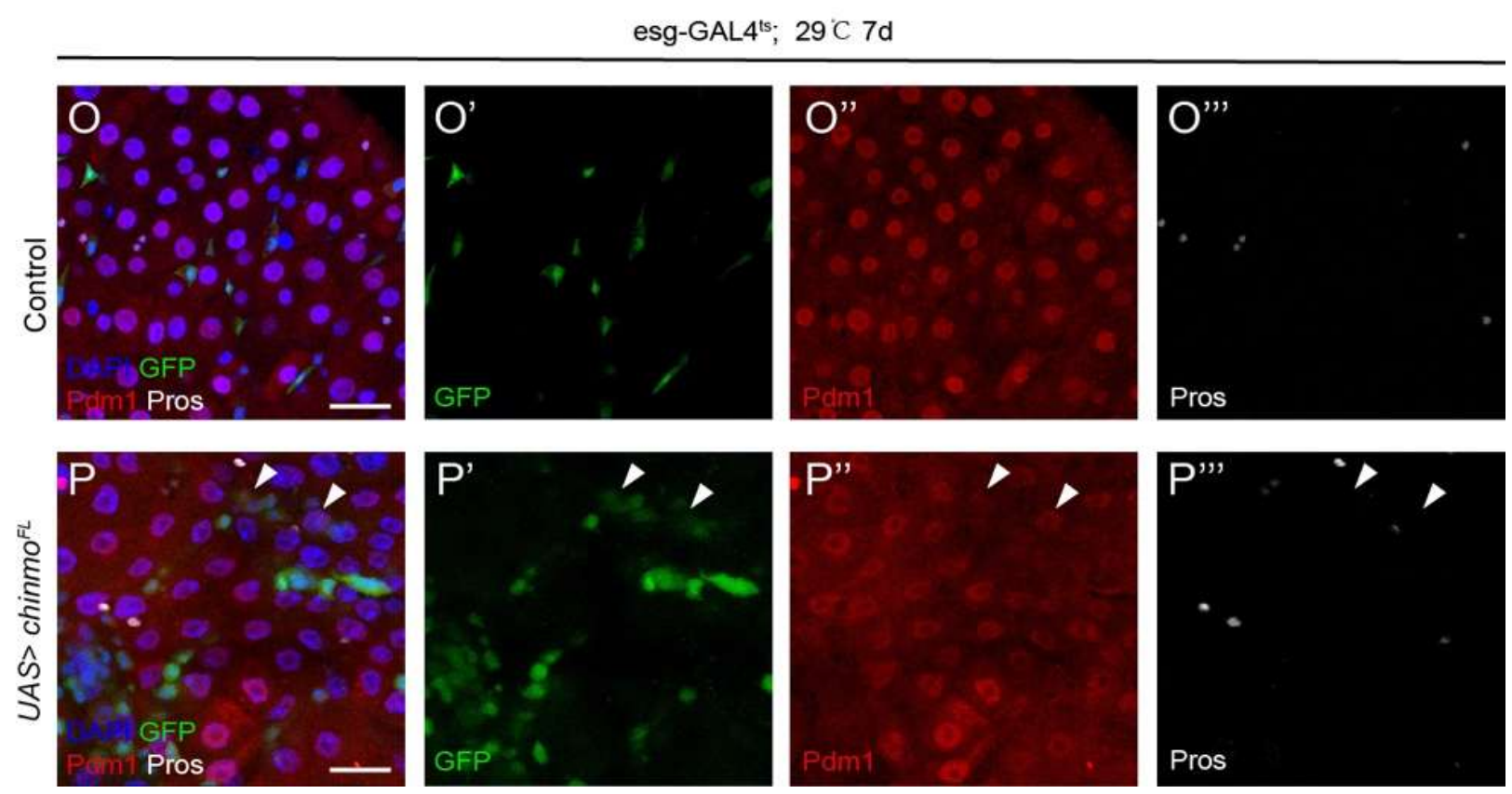

Minor:

3. Abstract: "ISC identity genes" should be modified to ISC and progenitor identity genes since many of these genes are found in ISC progeny as well.

Done as suggested.

4. DAPI labels in fluorescent panels are difficult to see. Please adjust colour to make them more visible.

We have adjusted the hue in all images to increase its visibility.

5. Figure 4C: esg should start with a lowercase e.

Corrected.

6. Fig. EV4M: label on x-axis is floating between panels M and N.

Corrected.

7. dpERK seems to decrease in Psc Su(z)2 clones (Fig. 2F) but increases after Chino overexpression (Fig. EV4F). The authors could discuss this discrepancy.

The increased dpERK signal following Chinmo overexpression is likely caused by mild temperature stress, since flies were raised at 29 °C in this experiment. We have added relevant discussion regarding this phenotype in the revised manuscript.

8. Title on page 6: "profiles" is a vague term. Perhaps "cells with neural-like gene expression"?

Done as suggested.

9. Page 7: Dl is considered an ISC-specific marker, thus the downregulated genes are "key intestinal ISC- or progenitors-specific markers".

Done as suggested.

10. Page 8: The title starting with "Comparative transcriptome analysis..." could describe the conclusion of this section. Perhaps a title that describes how Chinmo is required for Psc Su(z)2 tumour growth?

Done as suggested.

11. Page 8: Chinmo is expressed rather than activated. Please modify.

Corrected.

12. Page 10: the authors must modify their statement about H3K27me3 in PRC1 subunit clones. The authors state that "H3K27me3 levels were unaffected by Psc Su(z)2 deletion, nor by mutations in other PRC1 subunits" (Page 10). However, H3K27me3 does seem to be affected in sce or pc mutant clones.

We would like to clarify that global H3K27me3 levels are not markedly altered in Psc-Su(z)2 mutants or mutants of other PRC1 subunits. Although we observed cell-to-cell variation in H3K27me3 signal intensity, this heterogeneity is also present in midgut cells from wild-type flies. We have adjusted the fluorescence brightness to better illustrate that H3K27me3 levels remain largely unchanged within mutant clones.

13. The figure legends should include the number of clones, midguts etc., the number of experiments but also how many experiments are presented. The legend for Fig. 1F says that this experiment was performed 3 times, but data from only 6 midguts are shown. It seems like this data is from one of three experiments. Please clarify for all experiments.

As mentioned earlier, owing to the strong and stable phenotypes, a small number of midguts were analyzed per group in each replicate. Detailed sample information has been indicated in figure legends.

Dr. Rongwen Xi
National Institute of Biological Sciences
National Institute of Biological Sciences
7 Science Park Road.
Beijing 102206
China

Dear Dr. Xi,

Thank you for the submission of your final revised manuscript to our editorial offices. I have now received the report from the referee that was asked to re-evaluate the study, you will find below. As you will see, the referees now fully supports the publication of the study in EMBO reports. I also consider the remaining editorial requests as adequately addressed.

I am thus very pleased to accept your manuscript for publication in the next available issue of EMBO reports. Thank you for your contribution to our journal.

Your manuscript will be processed for publication by EMBO Press. It will be copy edited and you will receive page proofs prior to publication. Please note that you will be contacted by Springer Nature Author Services to complete licensing and payment information.

You may qualify for financial assistance for your publication charges - either via a Springer Nature fully open access agreement or an EMBO initiative. Check your eligibility: https://link.springer.com/journal/44319/how-to-publish-with-us

Should you be planning a Press Release on your article, please notify the production team via this form in order to coordinate publication and release dates: https://forms.cloud.microsoft/e/eH4A8JVP6g

If you have any questions, please do not hesitate to contact the Editorial Office. Thank you for your contribution to EMBO Reports.

Yours sincerely,

Achim Breiling
Senior Editor
EMBO Reports

>>> Please note that it is EMBO Reports policy for the transcript of the editorial process (containing referee reports and your response letter) to be published as an online supplement to each paper. If you do NOT want this, you will need to inform the Editorial Office via email immediately. More information is available here: https://link.springer.com/partners/embo-press/editorial-policies#Peer%20review

----

Referee #2:

The manuscript is suitable for publication in EMBO reports without further revision.

EMBO Press Author Checklist

Corresponding Author Name: Rongwen Xi	
Journal Submitted to: The EMBO Journal	
Manuscript Number: EMBOR-2025-62092V2	

USEFUL LINKS FOR COMPLETING THIS FORM
The EMBO Journal - Author Guidelines
EMBO Reports - Author Guidelines
Molecular Systems Biology - Author Guidelines
EMBO Molecular Medicine - Author Guidelines

Reporting Checklist for Life Science Articles (updated January
This checklist is adapted from Materials Design Analysis Reporting (MDAR) Checklist for Authors. MDAR establishes a minimum set of requirements in transparent reporting in the life sciences (see Statement of Task: 10.31222/osf.io/9sm4x). Please follow the journal's guidelines in preparing your
Please note that a copy of this checklist will be published alongside your article.

Abridged guidelines for figures
1. Data
The data shown in figures should satisfy the following conditions:
- the data were obtained and processed according to the field's best practice and are presented to reflect the results of the experiments in an accurate and unbiased manner.
- ideally, figure panels should include only measurements that are directly comparable to each other and obtained with the same assay.
- plots include clearly labeled error bars for independent experiments and sample sizes. Unless justified, error bars should not be shown for technical
- if n<5, the individual data points from each experiment should be plotted. Any statistical test employed should be justified.
- Source Data should be included to report the data underlying figures according to the guidelines set out in the authorship guidelines on Data

2. Captions
Each figure caption should contain the following information, for each panel where they are relevant:
- a specification of the experimental system investigated (eg cell line, species name).
- the assay(s) and method(s) used to carry out the reported observations and measurements.
- an explicit mention of the biological and chemical entity(ies) that are being measured.
- an explicit mention of the biological and chemical entity(ies) that are altered/varied/perturbed in a controlled manner.
- the exact sample size (n) for each experimental group/condition, given as a number, not a range;
- a description of the sample collection allowing the reader to understand whether the samples represent technical or biological replicates (including how many animals, litters, cultures, etc.).
- a statement of how many times the experiment shown was independently replicated in the laboratory.
- definitions of statistical methods and measures:
  - common tests, such as t-test (please specify whether paired vs. unpaired), simple χ2 tests, Wilcoxon and Mann-Whitney tests, can be unambiguously identified by name only, but more complex techniques should be described in the methods section;
  - are tests one-sided or two-sided?
  - are there adjustments for multiple comparisons?
  - exact statistical test results, e.g., P values = x but not P values < x;
  - definition of 'center values' as median or average;
  - definition of error bars as s.d. or s.e.m.

Please complete ALL of the questions below.
Select "Not Applicable" only when the requested information is not relevant for your study.

Materials

Newly Created Materials	Information included in the manuscript?	In which section is the information available? (Reagents and Tools Table, Materials and Methods, Figures, Data Availability Section)
New materials and reagents need to be available; do any restrictions apply?	Yes	

Antibodies	Information included in the manuscript?	In which section is the information available? (Reagents and Tools Table, Materials and Methods, Figures, Data Availability Section)
For **antibodies** provide the following information:<br>- Commercial antibodies: RRID (if possible) or supplier name, catalogue number and or/clone number<br>- Non-commercial: RRID or citation	Yes	

DNA and RNA sequences	Information included in the manuscript?	In which section is the information available? (Reagents and Tools Table, Materials and Methods, Figures, Data Availability Section)
**Short novel DNA or RNA including primers, probes**: provide the sequences.	Not Applicable	

Cell materials	Information included in the manuscript?	In which section is the information available? (Reagents and Tools Table, Materials and Methods, Figures, Data Availability Section)
**Cell lines:** Provide species information, strain. Provide accession number in repository **OR** supplier name, catalog number, clone number, and/**OR** RRID.	Not Applicable	
**Primary cultures:** Provide species, strain, sex of origin, genetic modification status.	Not Applicable	
Report if the cell lines were recently **authenticated** (e.g., by STR profiling) and tested for mycoplasma contamination.	Not Applicable	

Experimental animals	Information included in the manuscript?	In which section is the information available? (Reagents and Tools Table, Materials and Methods, Figures, Data Availability Section)
**Laboratory animals or Model organisms:** Provide species, strain, sex, age, genetic modification status. Provide accession number in repository **OR** supplier name, catalog number, clone number, **OR** RRID.	Yes	
**Animal observed in or captured from the field:** Provide species, sex, and age where possible.	Yes	
Please detail **housing and husbandry conditions**.	Yes	

Plants and microbes	Information included in the manuscript?	In which section is the information available? (Reagents and Tools Table, Materials and Methods, Figures, Data Availability Section)
**Plants:** provide species and strain, ecotype and cultivar where relevant, unique accession number if available, and source (including location for collected wild specimens).	Not Applicable	
**Microbes:** provide species and strain, unique accession number if available, and source.	Not Applicable	

Human research participants	Information included in the manuscript?	In which section is the information available? (Reagents and Tools Table, Materials and Methods, Figures, Data Availability Section)
If collected and within the bounds of privacy constraints report on age, sex and gender or ethnicity for all study participants.	Not Applicable	

Core facilities	Information included in the manuscript?	In which section is the information available? (Reagents and Tools Table, Materials and Methods, Figures, Data Availability Section)
If your work benefited from core facilities, was their service mentioned in the acknowledgments section?	Not Applicable	

Design

Study protocol	Information included in the manuscript?	In which section is the information available? (Reagents and Tools Table, Materials and Methods, Figures, Data Availability Section)
If study protocol has been **pre-registered, provide DOI in the manuscript**. For clinical trials, provide the trial registration number **OR** cite DOI.	Not Applicable	
Report the **clinical trial registration number** (at ClinicalTrials.gov or equivalent), where applicable.	Not Applicable	

Laboratory protocol	Information included in the manuscript?	In which section is the information available? (Reagents and Tools Table, Materials and Methods, Figures, Data Availability Section)
Provide DOI OR other citation details if **external detailed step-by-step protocols** are available.	Yes	

Experimental study design and statistics	Information included in the manuscript?	In which section is the information available? (Reagents and Tools Table, Materials and Methods, Figures, Data Availability Section)
Include a statement about **sample size** estimate even if no statistical methods were used.	Yes	
Were any steps taken to minimize the effects of subjective bias when allocating animals/samples to treatment (e.g. **randomization procedure**)? If yes, have they been described?	Yes	
Include a statement about **blinding** even if no blinding was done.	Not Applicable	
Describe **inclusion/exclusion criteria** if samples or animals were excluded from the analysis. Were the criteria pre-established?<br><br>If sample or data points were omitted from analysis, report if this was due to attrition or intentional exclusion and provide justification.	Not Applicable	
For every figure, are **statistical tests** justified as appropriate? Do the data meet the assumptions of the tests (e.g., normal distribution)? Describe any methods used to assess it. Is there an estimate of variation within each group of data? Is the variance similar between the groups that are being statistically compared?	Yes	

Sample definition and in-laboratory replication	Information included in the manuscript?	In which section is the information available? (Reagents and Tools Table, Materials and Methods, Figures, Data Availability Section)
In the figure legends: state number of times the experiment was **replicated** in laboratory.	Yes	
In the figure legends: define whether data describe **technical or biological replicates**.	Yes	

Ethics

Ethics	Information included in the manuscript?	In which section is the information available? (Reagents and Tools Table, Materials and Methods, Figures, Data Availability Section)
Studies involving **human participants**: State details of **authority granting ethics approval** (IRB or equivalent committee(s), provide reference number for approval.	Not Applicable	
Studies involving **human participants**: Include a statement confirming that **informed consent** was obtained from all subjects and that the experiments conformed to the principles set out in the WMA Declaration of Helsinki and the Department of Health and Human Services Belmont Report.	Not Applicable	
Studies involving **human participants:** For publication of **patient photos**, include a statement confirming that consent to publish was obtained.	Not Applicable	
Studies involving experimental **animals**: State details of **authority granting ethics approval** (IRB or equivalent committee(s), provide reference number for approval. Include a statement of compliance with ethical regulations.	Not Applicable	
Studies involving **specimen and field samples:** State if relevant **permits** obtained, provide details of authority approving study; if none were required, explain why.	Not Applicable	

Dual Use Research of Concern (DURC)	Information included in the manuscript?	In which section is the information available? (Reagents and Tools Table, Materials and Methods, Figures, Data Availability Section)
Could your study fall under dual use research restrictions? Please check biosecurity documents and list of **select agents and toxins** (CDC): https://www.selectagents.gov/sat/list.htm	Not Applicable	
If you used a select agent, is the security level of the lab appropriate and reported in the manuscript?	Not Applicable	
If a study is subject to dual use research of concern regulations, is the name of the **authority granting approval and reference number** for the regulatory approval provided in the manuscript?	Not Applicable	

Reporting

The MDAR framework recommends adoption of discipline-specific guidelines, established and endorsed through community initiatives. Journals have their own policy about requiring specific guidelines and recommendations to complement MDAR.

Adherence to community standards	Information included in the manuscript?	In which section is the information available? (Reagents and Tools Table, Materials and Methods, Figures, Data Availability Section)
State if relevant guidelines or checklists (e.g., **ICMJE, MIBBI, ARRIVE, PRISMA**) have been followed or provided.	Yes	
For **tumor marker prognostic studies,** we recommend that you follow the **REMARK** reporting guidelines (see link list at top right). See author guidelines, under 'Reporting Guidelines'. Please confirm you have followed these guidelines.	Not Applicable	
For **phase II and III randomized controlled trials**, please refer to the **CONSORT** flow diagram (see link list at top right) and submit the CONSORT checklist (see link list at top right) with your submission. See author guidelines, under 'Reporting Guidelines'. Please confirm you have submitted this list.	Not Applicable	

Data Availability

Data availability	Information included in the manuscript?	In which section is the information available? (Reagents and Tools Table, Materials and Methods, Figures, Data Availability Section)
Have **primary datasets** been deposited according to the journal's guidelines (see 'Data Deposition' section) and the respective accession numbers provided in the Data Availability Section?	Yes	
Were **human clinical and genomic datasets** deposited in a public access-controlled repository in accordance to ethical obligations to the patients and to the applicable consent agreement?	Not Applicable	
Are **computational models** that are central and integral to a study available without restrictions in a machine-readable form? Were the relevant accession numbers or links provided?	Not Applicable	
If publicly available data were reused, provide the respective **data citations in the reference list**.	Not Applicable	
